# Instrument validity: HIV and other sexually transmitted infections in homeless people

**DOI:** 10.1590/0034-7167-2021-0863

**Published:** 2022-10-03

**Authors:** Anna Cláudia Freire de Araújo Patrício, Richardson Augusto Rosendo da Silva, Ivoneide Lucena Pereira, Luipa Michele Silva, Maria Alzete de Lima, Maria Amanda Pereira Leite, Maria Hellena Ferreira Brasil, Suzanna Valéria Oliveira Souza

**Affiliations:** I Universidade Federal da Paraíba. João Pessoa, Paraíba, Brazil; II Universidade Federal do Rio Grande do Norte. Natal, Rio Grande do Norte, Brazil; III Universidade Federal de Catalão. Catalão, Goiás, Brazil

**Keywords:** Homeless Persons, HIV, Validation Study, Health Vulnerability, Sexually Transmitted Diseases., Personas Sin Hogar, VIH, Estudio de Validación, Vulnerabilidad en Salud, Enfermedades de Transmisión Sexual., Pessoas em Situação de Rua, HIV, Estudo de Validação, Vulnerabilidade em Saúde, Infecções Sexualmente Transmissíveis.

## Abstract

**Objectives:**

to validate, through the Item Response Theory, an instrument on vulnerability to HIV and other sexually transmitted infections in homeless people.

**Methods:**

a cross-sectional study carried out between February and May 2018 with 100 homeless people in a municipality in northeastern Brazil. A sociodemographic questionnaire was applied, and another with items referring to behaviors vulnerable to HIV and sexually transmitted infections (STIs). Subsequently, it was assessed through the Item Response Theory.

**Results:**

the items previous diagnosis of STIs (F=0.473), partner with STI symptoms (F=0.518), drug use (F=0.509), sex for money (F=0.552), STI symptoms (F=0.448), number of sexual partners (F=0.616), sharps sharing (F=0.398) and being a victim of sexual violence (F=0.347) were validated.

**Conclusions:**

the instrument proved to be validated, being able to identify vulnerability to HIV and other sexually transmitted infections in homeless people.

## RESUMO

**Objetivos:**

validar, por meio da Teoria da Resposta ao Item, instrumento sobre vulnerabilidade ao vírus da imunodeficiência humana e outras infecções sexualmente transmissíveis em pessoas em situação de rua.

**Métodos:**

estudo transversal, realizado entre fevereiro e maio de 2018 com 100 pessoas em situação de rua. Aplicou-se um questionário sociodemográfico, outro com itens referentes a comportamentos vulneráveis ao vírus da imunodeficiência humana e infecções sexualmente transmissíveis. Posteriormente, avaliou-se pela Teoria da Resposta ao Item.

**Resultados:**

validaram-se os itens diagnóstico prévio de infecções sexualmente transmissíveis (F=0,473), parceiro com sintoma de infecções sexualmente transmissíveis (F=0,518), uso de droga (F=0,509), sexo em troca de dinheiro (F=0,552), sintomas de infecções sexualmente transmissíveis (F=0,448), quantidade de parceiro sexual (F=0,616), compartilhamento de perfurocortante (F=0,398) e ser vítima de violência sexual (F=0,347).

**Conclusões:**

o instrumento demonstrou-se validado, capaz de identificar vulnerabilidade ao vírus da imunodeficiência humana e outras infecções sexualmente transmissíveis em pessoas em situação de rua.

## RESUMEN

**Objetivos:**

validar, a través de la Teoría de Respuesta al Ítem, un instrumento sobre vulnerabilidad al VIH y otras infecciones de transmisión sexual en personas en situación de calle.

**Métodos:**

estudio transversal realizado entre febrero y mayo de 2018 con 100 personas sin hogar en un municipio del Nordeste de Brasil. Se aplicó un cuestionario sociodemográfico y otro con ítems referentes a comportamientos vulnerables al VIH e infecciones de transmisión sexual (ITS). Posteriormente, se evaluó a través de la Teoría de Respuesta al Ítem.

**Resultados:**

los ítems diagnóstico previo de ITS (F=0,473), pareja con síntomas de ITS (F=0,518), consumo de drogas (F=0,509), sexo a cambio de dinero (F=0,552), síntomas de ITS (F=0,448), número de parejas sexuales (F=0,616), compartir objetos punzocortantes (F=0,398) y ser víctima de violencia sexual (F=0,347) han sido validados.

**Conclusiones:**

el instrumento demostró ser validado, pudiendo identificar la vulnerabilidad al VIH y otras infecciones de transmisión sexual en personas sin hogar.

## INTRODUCTION

In Brazil, the Institute of Applied Economic Research states that there are 134,374 homeless people (HP) registered in the *Cadastro Único* (an instrument that identifies and characterizes low-income families, allowing the government to better understand the socioeconomic reality of this population), but estimates that, in the general context of that country, there were 221,870 HP in March 2020. The largest number distributed was in the Southeast region, with 124,698, later the Northeast, with 38,237, in third place, the South with 33,591, the Midwest, with 15,718, and the North, with 9,626^(1)^.

HP live in an unhealthy environment, constituting a determinant in fragile health that is directly associated with the disease and low accessibility to health services. A study carried out in Tbilisi/Georgia showed that 98% of HP had heard about sexually transmitted infections (STIs), with the Human Immunodeficiency Virus/Acquired Immunodeficiency Syndrome (HIV/AIDS) having the highest prevalence, followed by hepatitis B, hepatitis C and syphilis. Most respondents knew the symptoms of STIs and understood that condom use reduces the risk of infection. However, more than 20% disagreed with the need to use condoms during anal intercourse, and 68.3% stated that the number of partners does not interfere with the chances of having STIs. Although they have heard about it, they do not really know what the disease is and have not shown good awareness^(2)^.

The determinants that contribute to increased vulnerability to HIV/AIDS infection and other STIs in HP include fragile knowledge, wrong beliefs and attitudes about HIV, lack of access to support networks, rejection and discrimination, low frequency of drug use and condoms^(3)^.

Therefore, given the precarious living conditions of HP, the scarcity of studies in this area, the fragility of public policies, needy social support, discrimination, stigma, greater exposure to vulnerable behaviors and fragility of training of health professionals involving this theme, it is necessary to investigate factors related to HP’s health and disease, justifying the performance of this study.

From this perspective, nursing can advance to contribute to the creation of public policies, work strategies and change of care models that consider issues related to vulnerability in HP’s health care, demonstrating the relevance of this study.

Thus, the following question emerged: is it possible to validate an instrument on HIV and other STIs capable of assessing the vulnerability of these diseases in HP?

## OBJECTIVES

To validate, through the Item Response Theory, an instrument on vulnerability to HIV and other STIs in HP.

## METHODS

### Ethical aspects

The research followed the ethical principles established in Resolution 466/2012 of the Brazilian National Health Council, which concerns the Regulatory Guidelines and Norms for Research Involving Human Beings^(4)^. It was approved by the Research Ethics Committee. Moreover, each participant received two copies of the Informed Consent Form (ICF), one of which was signed and returned to the researcher.

### Study design, period, and site

This is a part of a doctoral thesis that consisted of a cross-sectional quantitative approach research, which took place from February to May 2018, based on the Strengthening the Reporting of Observational Studies in Epidemiology (STROBE), recommended by the Equator network^(5)^. The research was carried out at *Casa da Acolhida para Pessoas em Situação de Rua* (a center that welcomes people on the streets), in João Pessoa, Paraíba, Brazil and at the Specialized Reference Center for Homeless Population (CENTRO POP - *Centro de Referência Especializado para População em Situação de Rua*), João Pessoa, Paraíba, Brazil.

CENTRO POP is part of the services offered by the Unified Social Assistance System (SUAS - *Sistema Único de Assistência Social*), through shared management at three levels (municipal, state and federal). It is regulated by the Brazilian National Council for Social Assistance (CNAS - *Conselho Nacional de Assistência Social*), through the approval of a Special Social Protection Service of Medium Complexity established by the Brazilian National Policy for Homeless Population, through Decree 7053/2009^(6)^.

It is noteworthy that CENTRO POP is a space that works from Monday to Friday, in the morning and afternoon shifts, serving an average of 50 HP daily, who must be over 18 years old. They offer three meals a day, a place to rest and to perform personal hygiene. Furthermore, they have a support team composed of an educator, a social worker and a psychologist. They carry out activities to encourage harm reduction, make the necessary referrals to reference health services, promote the improvement of interpersonal relationships, respect, autonomy and solidarity. In this place, HP cannot enter if they are using drugs, weapons, sharps and alcoholic beverages, in addition to not allowing physical and verbal violence. Access can occur by spontaneous demand, referral by the service network or by a Specialized Service of Social Approach.

*Casa da Acolhida* shelters HP over 18 years old for an average period of 60 days. When they try to re-socialize, they help with the insertion into the job market, removal of documents and referrals to other services. It works 24 hours, seven days a week. In this place, people sleep, eat, do their personal hygiene, in addition to developing recreational activities (games, capoeira circles and arts) throughout the period of residence.

In this way, it is noteworthy that the aforementioned places have a clean, calm and individual environment, allowing ethics, commitment and responsibility at all times of data collection, reducing the possibility of risks to research participants.

It should be noted that these data collection sites were chosen because it would not be possible to carry out this research with HP who were not linked to them, since rapid tests were carried out, which require important care for the safety of participants and researchers, such as: preservation of participants’ identity; pre- and post-test reception; clean environment; use of disposable materials and disposal in an appropriate place; adequate lighting; and referral to a reference hospital for infectious diseases, in case of positivity in the aforementioned rapid tests.

### Population or sample; inclusion and exclusion criteria

The population comprised 110 HP, and the sample consisted of 100 individuals, 15 from *Casa da Acolhida* and 85 from CENTRO POP. To calculate the sample, 95% confidence and 3% margin of error were used, using the formula for sampling, as proposed^(7)^: n = N.p.q.(Z)^2^ divided by p.q(z)^2^ + (N-1). E^2^, where N= population (110), n= sample, p.q= population proportion (0.25), confidence = 95%, E= error (3% = 0.03), Z= critical value (1.96). Thus, n =110 . 0.25 . (1.96)^2^, divided by 0.25 . (1.96)^2^ + (110-1) . (0.03)^2^. A sample of n=99.801 was totaled, rounded to 100.

Individuals who were 18 years of age or older, with verbal communication skills and who were able to contribute to the research at the time of data collection were included. Those HP who were aggressive or were using drugs or alcohol at the time of data collection were excluded.

### Study protocol

The data collection protocol was carried out in eight steps, as described below.

Step one comprised the request for authorization to carry out the research. This procedure was requested from the person responsible for the data collection site at the Department of Social Assistance of João Pessoa, subsequently, communication to the employee of the respective location. Submission to the Research Ethics Committee was also carried out. Still at this step, the data collection team, composed of a doctoral student nurse and six undergraduate nursing students, underwent a six-hour training, in which there was a description of the instruments used in the research and simulation of their application, standardizing language and collection data form.

In step two, the researcher introduced herself to the data collection sites, explained all the research steps and, with the help of employees at the data collection site, invited some HP to participate in the survey, scheduling data collection seven days in advance.

In step three, on the date scheduled for data collection, the explanation of all phases of data collection and the research objectives was carried out individually. At this point, step four took place, with the delivery of two copies of the ICF, returning a signed copy or with a fingerprint to the researcher.

After this moment, step five took place, with prior reception, individually, in which concepts were explained and any doubts were clarified, as well as the rapid tests that were applied for HIV, hepatitis B, hepatitis C and syphilis. To perform the rapid tests for hepatitis B and C, syphilis and HIV, recommendations were followed^(8-10)^, using the immunochromatography or lateral flow test. To start the collection of the rapid tests, the sample was identified with patients’ initials, then antisepsis was performed on patients’ fingerprint and puncture with a disposable lancet. The larger end of the pipette was lightly pressed until the blood was sucked, then two drops of blood were dispensed into the round hole of the test and one drop of the reagent. It is noteworthy that the rapid test for HIV was from *Bioclin*, the rapid test for syphilis, from *Alere*, the rapid test for hepatitis B, from *Biomérieux* brand, and the rapid test for hepatitis C, from *Alere*. The HIV, syphilis and hepatitis tests were considered positive when two lines appeared, one in the control space (C) and the other in the test space (T), being considered negative when only one line appeared in the ‘C’.

In step six, the sociodemographic questionnaire was first applied, as it is necessary to identify the rapid test and the instruments with the research volunteer’ initials. Subsequently, the rapid test was applied in a private room, since its result is not obtained immediately; in this way, time was optimised. The other instruments were collected soon after.

After applying all the instruments, the research volunteer was invited to enter a room individually, to receive the exam result (step seven). After explaining the result, further counseling was carried out, regardless of being positive or not, with the presence of a social worker and a psychologist from the unit where the data collection was being carried out.

In step eight, those with positive results were referred to the referral health service for infectious diseases in the municipality of João Pessoa, Paraíba, Brazil.

### Analysis of results, and statistics

Data were processed using the Statistical Package for the Social Sciences (SPSS) - version 19.0 and the free R package 3.3.1 program, in the public domain. Also, the MPLUS software was used. The statistics followed steps, starting in a descriptive way with absolute and relative frequency, mean, standard deviation, maximum and minimum of the variables. Subsequently, the questionnaire items were validated, consisting of 16 questions regarding vulnerability to HIV and other STIs, through the Item Response Theory.

The 16 items that made up the questionnaire on vulnerability to HIV and other STIs, with their respective score scores, were: age of first sexual intercourse (< 18 years old = 0; ≥ 18 years old = 01); previous diagnosis of any STI (yes = 0; no =01); use condom in every sexual relation (yes = 01; no = 0); reason for not using condoms (not applicable = 01; any time = 0); type of sexual partner (casual and/or sex worker = 0; boyfriend(girlfriend) and/or husband(wife)/fixed = 1); use drugs (yes = 0; no = 01); sex with drug user (yes = 0; no = 01); sex for money (yes = 0; no = 01); already presented any STI symptom (yes = 0; no = 01); number of sexual partners (1 = 01; >1 = 0); pain during sex (yes = 0; no = 01); sex with a partner with an injury to the genital area (yes = 0; no = 01); partner with STI symptom (yes = 0; no = 01); sharps sharing (yes = 0; no = 01); needle reuse (yes = 0; no = 01); already suffered violence (yes = 0; no = 01).

Thus, one point was assigned for non-vulnerable behaviors and zero for vulnerability. The Item Response Theory (IRT) was used for two parameters, being discrimination(a) and difficulty(b). The parameter hit by chance (c) was discarded because, in this type of questionnaire, there is no right or wrong answer, not being influenced by ‘guesses’ to find a correct alternative, since individuals responded based on their individual behaviors. Discrimination values were not accepted when negative, and a not very high degree of difficulty was expected.

For difficulty(b), it was considered acceptable between -2 and + 2; for discrimination, values between 0 and +2 were considered. Factor loading was considered adequate when it obtained a value of at least 0.30, so that the item remained in the instrument.

The relationship between responses that indicate absence of vulnerability (vertical axis) and individuals’ respective ability (horizontal axis) was described through the Item Characteristic Curve (ICC), which was represented by a sigmoid curve (S-shape).

Additionally, we emphasize the factor analysis with p-value that must be >0.05, Root Mean Square Error of Approximation (RMSEA), at most 0.08, Root Mean Square Residual (RMSR), with a desirable value of 0.07, Tucker-Lewis Index (TLI) and Comparative Fit Index (CFI), with a minimum value of 0.90. The item factor loading was also used, being acceptable when >0.30^(11)^.

In addition to this, the diagnostic test was carried out using the Receive Operation Characteristics (ROC) curve and the cut-off point, in order to find the instrument’s score on vulnerability to HIV, which it represents as a risk, as well as its respective comparison with the gold standard test (for HIV, a rapid test was used). The closer the ROC curve reaches number one, the better the test.

In general, the upper leftmost point on the ROC curve is chosen, noting that the greater the area under this curve, the more accurate the test. Considering that the incidence of this disease (HIV) is very low, in the studied sample, in this research, more attention will be given to sensitivity. In this study, for the cut-off point, the parameters sensitivity, specificity, accuracy, positive and negative predictive value were listed. Sensitivity is the probability that a test will be positive when the disease is present. Specificity is a test’s ability to be negative when there is no diagnosis. Accuracy refers to how well a test correctly discriminates between health and disease. Positive predictive value refers to whether the individual actually has the disease. The negative predictive value is the probability that the person with a negative test is not sick. A high sensitivity will generate a high negative predictive value, a high specificity will generate a high positive predictive value. High cut-off points refer to low sensitivity and high specificity, and low cutoff points are very sensitive and unspecific^(12)^.

## RESULTS

Of the 100 HP who participated in the research, 84% identified themselves as men, aged between 18 and 62 years, living on the streets for a minimum of two months and a maximum of 55 years. The main reason for becoming a homeless person, corresponding to 45%, was family confrontation, followed by drug use, 35%. As for the rapid tests for HIV, 5% were positive, 29% with a positive rapid test for syphilis, 1% for hepatitis b and none for hepatitis c, as shown in Table 1.

**Table 1:** Rapid tests and behaviors vulnerable to infection with the Human Immunodeficiency Virus and sexually transmitted infections of homeless people, João Pessoa, Paraíba, Brazil, 2018

Variables	n	%
RAPID TESTS		
Syphilis		
Negative	70	70
Positive	29	29
Did not do it	1	1
HIV^*^		
Negative	94	94
Positive	5	5
Did not do it	1	1
Hepatitis B		
Negative	98	98
Positive	1	1
Did not do it	1	1
Hepatitis C		
Negative	99	99
Did not do it	1	1
Positive	-	-
BEHAVIORS VULNERABLE TO HIV AND OTHER STIS^*^		
Age of first sexual intercourse?		
Less than 18 years old	91	91
13 years	21	21
12 years	19	19
14 years	14	14
15 years	14	14
17 years	8	8
16 years	6	6
11 years	5	5
10 years	3	3
9 years	1	1
Older than or equal to 18 years	9	9
18 years	6	6
20 years	2	2
19 years	1	1
Have you had previous diagnosis of any STI?		
No	75	75
Yes	25	25
Which STI have you ever had?		
Syphilis	12	12
Gonorrhea	7	7
Candidiasis	5	5
Trichomoniasis	1	1
Do you use condom in every sexual relation?		
No	75	75
Yes	25	25
Do you use condoms in anal sex?		
No	35	35
Yes	28	28
Sometimes	27	27
Did not do it	10	10
Do you use condoms during vaginal sex?		
No	37	37
Yes	32	32
Sometimes	31	31
Do you use condoms during oral sex?		
No	45	45
Sometimes	25	25
Yes	24	24
Did not do it	6	6
What are the reasons for not using condoms?		
Trust	39	39
Prefer skin to skin	23	23
No time to put	21	21
Not applicable	11	11
They might think I am HIV positive	4	4
Condom is not available	1	1
Reduces pleasure	1	1
Have you ever presented any STI symptom (discharge, burning, itching)?		
No	57	57
Yes	43	43
Number of sexual partners?		
One	74	74
More than one	26	26
Needle reuse?		
No	95	95
Yes	5	5

* *HIV - Human Immunodeficiency Virus; STIs - sexually transmitted infections.*

After analysis through the IRT, the questionnaire on behaviors vulnerable to HIV and other STIs, previously composed of 16 items, validated nine items as capable of measuring this variable, as shown in Table 2. The seven items that were not validated were age at first sexual intercourse, condom use in all relationships, reason for not using condoms, pain during sex, sex with a partner with an injury to the genital area, partner with STI symptom and needle reuse.

**Table 2:** Item Response Theory parameters, factor loading and adjustment indices of factor analysis for instrument referring to behaviors vulnerable to Human Immunodeficiency Virus infection and sexually transmitted infections of homeless people, João Pessoa, Paraíba, Brazil, 2018

Items	Discrimination (a)	Difficulty (b)	Factor loading (F)
Previous diagnosis of STIs^*^	0.917	1.402	0.473
Partner with STI symptom	1.032	-0.390	0.518
Illicit drug use	1.007	-1.251	0.509
Customer/partner uses drugs	1.358	-0.172	0.624
Sex for money	1.128	1.519	0.552
STI symptoms	0.854	0.376	0.448
Number of sexual partners	1.330	1.940	0.616
Sharps sharing	0.739	4.316	0.398
Victim of sexual violence	0.630	2.968	0.347
% variation explained	-	-	25.6
p-value	-	-	0.054
RMSEA	-	-	0.0691
RMSR	-	-	0.0926
TLI	-	-	0.7074
CFI	-	-	0.7805

* *STIs - sexually transmitted infections.*

It can be verified that all the items that were accepted present difficulties between -2 and +2, except for sharps sharing and having been a victim of sexual violence, which can be justified by the fact that they are not items that are judged right or wrong, but address individuals’ behavior, presenting good discrimination and reaching the desired value between 0 and +2, in addition to factorial load greater than 0.30; therefore, these items were considered valid. It is also noteworthy the significance value of the instrument by p>0.05, RMSEA of up to 0.08. This analysis confirms these items as essential in measuring vulnerability to HIV and other STIs in HP.

**Figure 1:**
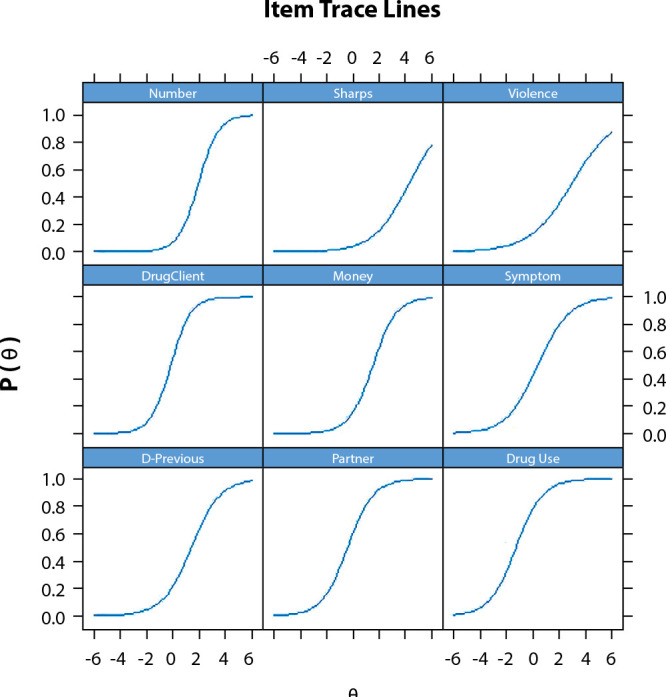
Characteristic Curve of the instrument’s item referring to behaviors vulnerable to infection with the Human Immunodeficiency Virus and sexually transmitted infections of homeless people, João Pessoa, Paraíba, Brazil, 2018

**Figure 2:**
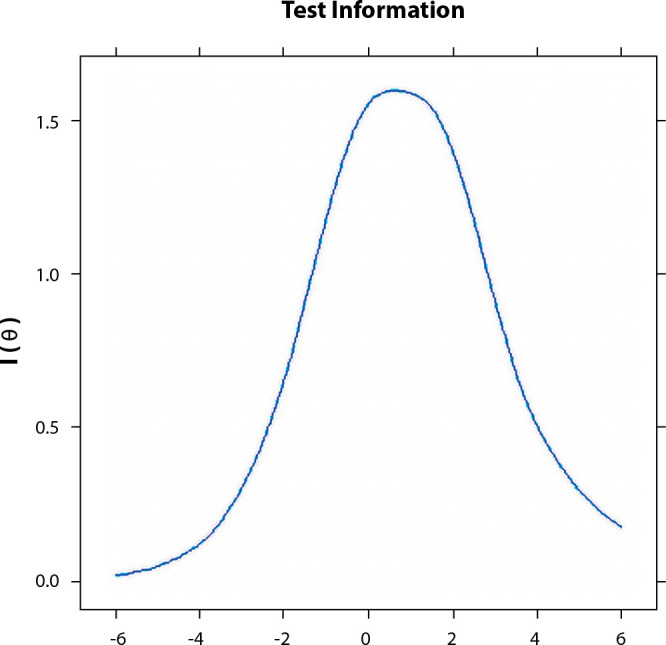
Curve referring to all items of the instrument of vulnerable behaviors when falling ill related to infection with the Human Immunodeficiency Virus and other sexually transmitted infections of homeless people, João Pessoa, Paraíba, Brazil, 2018

Figures 1 and 2 demonstrate the explanatory capacity of the phenomenon, as they present an approximate S-shape, also revealing the aptitude of the study subjects. The Item Characteristic Curve, represented in Figures 1 and 2 of the instrument of vulnerable behaviors to HIV and other STIs, reveals that the nine items are able to explain the phenomenon, as they present a sigmoid curve.

*Number - number of sexual partners; Sharps - sharps sharing; Violence - already suffered violence; DrugClient - sex with a partner who uses drugs; Money - sex for money; Symptom - previous STI symptoms; D-Previous - previous diagnosis of sexually transmitted infections; Partner - partner with STI symptom; Drug Use - illicit drug use by people themselves.*

The HIV test presented a ROC curve with an area of 0.680 and a 95% confidence interval between 0.55 and 0.808. For the cut-off point, two points were candidates for the best cut-off point: (0.200; 0.234) and (0.60; 0.319). The first was ruled out for its low sensitivity. Table 3 presents the cut-off point for this test and the diagnostic properties for this point of the ROC curve.

**Table 3:** Human Immunodeficiency Virus Test, prepared with a cutoff score equal to 4, compared to the gold standard (ELISA Test) and properties of the Human Immunodeficiency Virus test with a cut-off equal to 4 for a positive diagnosis of homeless people, João Pessoa, Paraíba, Brazil, 2018

HIV test score ^*^	Standard test (ELISA)	
	Positive	Negative
Positive ≥ 4	3	30
Negative < 4	2	64
**Parameter**	**%**	**95% CI**
Sensitivity	60.00	14.66 a 94.72
Specificity	68.08	57.67 a 77.32
Accuracy	67.68	57.53 a 76.73
Positive predictive value (PPV)	9.09	1.92 a 24.33
Negative predictive value (NPV)	96.97	89.48 a 99.63

* *HIV - Human Immunodeficiency Virus.*

## DISCUSSION

It is important to highlight the behaviors that are vulnerable to HIV infection and other STIs present in this study and that corroborate other studies with HP, such as early initiation of sexual intercourse, previous diagnosis of STI, weakness in condom use, drug use and sex for money. Moreover, in Brazil, the prevalence of syphilis in this population is related to low education, sex under the influence of drugs, previous genital ulcer and other STIs, in addition to the number of sexual partners^(13)^.

Another item found in this research is sex for money, which is often characterized as a habit in HP, and may be associated with drug use, making this practice extremely vulnerable to STIs. It is also noteworthy that money enters this process for reasons of survival, in exchange for food, hygiene and basic physiological needs^(14-15)^.

As for the validated items referring to sharing sharps and sex for money, a study carried out with HP in Ibadan highlighted this variable as a behavior that predisposes to HIV and other STIs, which can be justified by the lack of physical, social, emotional and financial empowerment, in addition to being considered easy victims to be coerced/forced^(16)^.

Thus, behaviors vulnerable to HIV infection and other STIs, practiced by HP, were also evidenced in other research, including multiple sexual partners, sharing of personal objects, sexual harassment, in addition to the fragility in knowledge about STIs^(16)^.

In the research in question, there was a predominance of illicit drug use, and it is known that the sex with drug user contributes to the increase in HIV/AIDS infection, as it directly influences the use of condoms, in addition to being related to physical and/or sexual violence^(17)^.

Sex for money, drug use and disuse of condoms, carried out by the sample of this research, deserve to be highlighted, because people living with HIV who perform these practices have a detectable viral load, increasing the possibility of transmission^(18)^. It is also noteworthy that the use of injectable drugs increased HIV transmission among HP, even in cities that have prevention programs^(19)^.

The use of illicit drugs and having sex with a client/partner who uses drugs, items validated in the present study, are factors intertwined with vulnerability to HIV and other STIs, since it compromises individuals’ alertness, making them more susceptible not using condoms and any other safer sex practices^(20)^.

Still referring to validity of questionnaires on HIV and other STIs, it is noteworthy that the ROC curve detected cutoff points and their diagnostic parameters that are capable of tracking the probability of actually contracting the disease, when compared to the gold standard test. This becomes relevant as it can be used as a method to detect or rule out the probability of having the disease or not.

As for strategies to minimize STIs in the homeless population, research reveals three priorities cited by this public: organization of support groups; counseling and assistance groups (employment); education for behavior change. All these priorities were carried out in partnership with the government^(16)^.

The fact of being on the streets is directly associated with the high rate of HIV infection and other STIs, being 1.55 times higher when compared to the general population, justified by the fragility of access to harm reduction services, in addition to injecting and engaging in unsafe sexual behaviors, which in turn are intertwined with unemployment, hunger, mental health disorders, inequality, and drug use^(21)^.

As a validated item to detect vulnerability to HIV and other STIs, the previous diagnosis of STIs, having symptoms of STIs and having sexual intercourse with a partner with STI symptom characterize an important problem and a challenge for health professionals. A study with HP revealed that 39.5% presented symptoms of STIs, with 13.8% genital discharge of bad smell, 11.2% genital ulcers and 14.5% burning sensation during urination. However, the demand for care and treatment follow-up was low, also demonstrating the need for health professionals to train themselves to address and understand the context of HP^(22)^.

As for the number of sexual partners, validated in the present study as effective in verifying vulnerability to HIV and other STIs in HP, it is noteworthy that, in another research with HP, 30.1% of the sample had multiple partners and 68% of the sexual relations were unprotected. This item was shown to be essential to verify vulnerability to HIV and other STIs, as well as the need to enable greater and better accessibility to health services, leading to the care of professionals trained to work within reference centers or shelters for HP, minimizing these risks^(23)^.

Also, it is emphasized to be a victim of sexual violence, a validated item that also characterizes vulnerability to HIV and other STIs, constituting an important factor to be investigated, and, many times, the approach to patients is left aside in the anamnesis. HP become exposed to various types of violence and, sometimes, submit to not denounce the aggressor, due to a threat to life, valuing survival^(24)^.

Finally, the need to structure and implement intersectoral public policies to reduce HP’s vulnerability to HIV and other STIs is highlighted. For this, nursing plays a fundamental role and can contribute to minimize unfavorable outcomes, such as hospitalizations due to these diseases.

### Study limitations

The limitations are related to the fact that the research was carried out in a single city, as well as the fact that the participants attended shelters, which could influence the look at the aspects analyzed in the study. It is noteworthy that 84% of the sample were men, a characteristic that may have impacted the responses of some items.

### Contributions to nursing, health, and public policies

With regard to communicable diseases, this research enabled a mapping, listing factors that contribute to their vulnerability, contributing to the visibility of this population that needs health care, with a view to minimizing the transmission of diseases, as well as treatment and restoration, in addition to complying with one of the pillars of health promotion, which consists of promoting citizens’ well-being, in this case, HP.

Nursing is one of the members of the multidisciplinary team in the outreach street office and in primary care that assists these people. Therefore, the results of this research demonstrate relevant and easy factors to be applied in clinical practice of disease prevention and health promotion. By incorporating this data into the anamnesis, nurses can predict situations of potential vulnerability to HIV and other STIs, leading to rapid, effective and assertive care.

## CONCLUSIONS

The research achieved the proposed objective, validating nine important items capable of detecting vulnerability to HIV and other STIs in HP: previous diagnosis of STIs; having sexual intercourse with a partner with STI symptom; illicit drug use; having sex with a client/partner who uses drugs; sex for money; having symptoms of STIs; number of sexual partners; sharps sharing; victim of sexual violence.

HP have health conditions that can cause illness, requiring changes in public health systems, in order to insert them into comprehensive care through qualified care.

In this way, this research is relevant in the social and public health context, as it can support the conduct of professionals who assist HP, in the sense of increasing disease prevention actions and early detection of factors that influence the illness by HIV and other STIs.

It is suggested the creation of more support houses and training of health professionals, in order to understand the demand and the reality experienced by HP, providing access to health care and support groups that promote dignified training for reintegrating HP.

## SUPPLEMENTARY MATERIAL

This article is an excerpt of a doctoral thesis entitled ‘*Condições clínicas associadas às pessoas em situação de rua*’ [Internet]. 2019. *Programa de Pós-Graduação em Enfermagem, Universidade Federal do Rio Grande do Norte*. Available from: https://repositorio.ufrn.br/handle/123456789/28481

0034-7167-reben-75-06-e20210863-sup01
