# Supplementary material for: Instrument validity: HIV and other sexually transmitted infections in homeless people
Source: Rev Bras Enferm. 2022 Oct 3;75(6):e20210863. doi: 10.1590/0034-7167-2021-0863 (PMC9749765; doi:10.1590/0034-7167-2021-0863)
Supplement: 0034-7167-reben-75-06-e20210863-sup01 [file 0034-7167-reben-75-06-e20210863-sup01.pdf]

UNIVERSIDADE FEDERAL DO RIO GRANDE DO NORTE
CENTRO DE CIÊNCIAS DA SAÚDE
DEPARTAMENTO DE ENFERMAGEM
PROGRAMA DE PÓS-GRADUAÇÃO EM ENFERMAGEM
NÍVEL DOUTORADO

ANNA CLÁUDIA FREIRE DE ARAÚJO PATRÍCIO

CONDIÇÕES CLÍNICAS ASSOCIADAS ÀS PESSOAS EM SITUAÇÃO DE RUA

NATAL - RN
2019

ANNA CLÁUDIA FREIRE DE ARAÚJO PATRÍCIO

CONDIÇÕES CLÍNICAS ASSOCIADAS ÀS PESSOAS EM SITUAÇÃO DE RUA

Tese apresentada ao Programa de Pós-Graduação em Enfermagem da Universidade Federal do Rio Grande do Norte, como requisito para obtenção do título de doutora em Enfermagem.
Área de Concentração: Enfermagem na atenção à saúde.
Linha de Pesquisa: Enfermagem na Vigilância à Saúde.
Orientador: Profº Drº Richardson Augusto Rosendo da Silva.

NATAL – RN

2019

Universidade Federal do Rio Grande do Norte - UFRN

Sistema de Bibliotecas - SISBI

Catalogação de Publicação na Fonte. UFRN - Biblioteca Central Zila Mamede

Patricio, Anna Claudia Freire de Araujo.
Condições clínicas associadas às pessoas em situação de rua / Anna Claudia Freire de Araujo Patricio. - 2019.
141 f.: il.

Tese (doutorado) - Universidade Federal do Rio Grande do Norte, Centro de Ciências da Saúde, Programa de Pós-graduação em Enfermagem, Natal, RN, 2019.
Orientador: Prof. Dr. Richardson Augusto Rosendo da Silva.

1. Pessoas em situação de rua - Tese. 2. Saúde mental - Tese. 3. HIV - Tese. 4. Sífilis - Tese. 5. Tuberculose - Tese. I. Silva, Richardson Augusto Rosendo da. II. Título.

RN/UF/BCZM CDU 364.6-058.51:616-083

Elaborado por Fernanda de Medeiros Ferreira Aquino - CRB-15/301

FOLHA DE APROVAÇÃO

PATRÍCIO, A. C. F. A. **Condições clínicas associadas às pessoas em situação de rua**. Natal, 2019. Tese, 141 folhas. Tese (Doutorado em Enfermagem). Programa de Pós-Graduação em Enfermagem, Universidade Federal do Rio Grande do Norte (RN), 2019.

CONDIÇÕES CLÍNICAS ASSOCIADAS ÀS PESSOAS EM SITUAÇÃO DE RUA

Tese apresentada ao Programa de Pós-Graduação em Enfermagem da Universidade Federal do Rio Grande do Norte, como requisito para obtenção do título de doutora em Enfermagem.

Aprovado em: 13/11/2019.

BANCA EXAMINADORA

____
Prof° Dr° Richardson Augusto Rosendo da Silva
(Orientador-Presidente)
Universidade Federal do Rio Grande do Norte

____
Prof. Drª Maria Alzete Lima
(Membro Titular da Banca - Interno)
Universidade Federal do Rio Grande do Norte

____
Prof. Drª Mirella Alves da Cunha
(Membro Titular da Banca - Interno)
Universidade Federal do Rio Grande do Norte

____
Prof. Drª Luipa Michele Silva Cabral
(Membro Titular da Banca - Externo)
Universidade Federal do Goiás – Regional Catalão

____
Prof. Drª Khivia Kiss da Silva Barbosa
(Membro Titular da Banca - Externo)
Universidade Federal de Campina Grande

“A água do mar está gelada demais e ninguém se arriscou a entrar. Já reparou que as pessoas fogem do que pode tirá-las da temperatura ambiente? Eu não sou desse jeito. Gosto da adrenalina do que tiver que ser será. Sempre é, sempre será. Tenho pavor ao raso, quando metade do corpo fica dentro e metade não. Eu sou dos fundos, entende?”

(Ryane Leão, 2017)

À Laura (filha) e Rosanjala (mãe),

Pelos ensinamentos, renúncias e amor sem medida.

AGRADECIMENTOS

A Deus e Nossa Senhora por conduzir cada passo da minha existência;

A minha família pelo direcionamento, ensinamento, amparo e amor;

A Profº Dº Richardson Augusto Rosendo da Silva (orientador) pela confiança, ensinamentos, paciência, fortalecimento, conforto;

A todas as pessoas em situação de rua que participaram da pesquisa e para além disso, ensinaram-me valores essenciais;

Aos funcionários da Casa da Acolhida de Pessoas em Situação de Rua e do Centro de Referência Especializado para População em Situação de Rua pela receptividade, por disponibilizar ambiente propício para coleta de dados, bem como, pela participação e colaboração no acolhimento pré e pós teste rápido;

Aos meus amigos companheiros de pesquisa e coleta de dados: Brenda; Deysianne, Géssica; Ingrid; Iana; Lidiane, Milenna, Maria Hellena, Maria Amanda, Marina; Rôseane; Thayná; Thays;

A Jiovana Santos, Gracimary, Karolina, Manuella e Marta pela escuta ativa em todos os momentos;

A Valentim e Sofia por proporcionarem alegria aos meus dias;

Aos membros da banca que colaboraram efetivamente para construção e aprimoramento desta pesquisa;

A Secretaria de Saúde do Estado da Paraíba, na pessoa de Ivoneide Lucena, pela disponibilidade de Testes Rápidos que propiciaram a realização desta pesquisa;

Aos professores do Programa de Pós Graduação em Enfermagem da UFRN pelos conhecimentos compartilhados;

A Profª Dª Clélia Simpson por compartilhar momentos importantes e por muitas vezes percorrer a estrada em sua companhia;

Aos funcionários do Programa de Pós Graduação em Enfermagem da UFRN;

A todos os meus alunos que acreditam em mim e mantém acesa a chama do encantado mundo do ensinar e aprender, dar e receber, ser e existir;

Aos colegas de trabalho da Universidade Federal de Uberlândia/MG.

PATRÍCIO, A. C. F. A. **Condições clínicas associadas às pessoas em situação de rua**. Natal, 2019. Tese, 141 folhas. Tese (Doutorado em Enfermagem). Programa de Pós-Graduação em Enfermagem, Universidade Federal do Rio Grande do Norte (RN), 2019.

RESUMO

Trata-se de um estudo transversal, realizado com 100 pessoas em situação de rua no município de João Pessoa, Paraíba, com o objetivo de analisar condições clínicas associadas às pessoas em situação de rua.. Os dados foram coletados no período entre fevereiro e maio de 2018. Para tanto utilizou-se: um roteiro de entrevista estruturado, Escala Self-Reporting Questionnaire, Escala de Ansiedade de Beck, Escala de Depressão de Beck, Escala de Desesperança de Beck, Escala de Resiliência, Escala de Depressão de Hamilton e Questionário de Vulnerabilidade ao HIV/aids. Além disso, foram realizados Testes rápidos para HIV, sífilis, hepatite B e C. A pesquisa foi aprovada pelo Comitê de Ética em Pesquisa com Seres Humanos da Universidade Federal da Paraíba, conforme parecer número 2456847, CAAE 79486517000005176. Inicialmente validou-se o questionário sobre vulnerabilidade ao HIV, Tuberculose e outras Infecções Sexualmente Transmissíveis por meio da Teoria de Resposta ao Item. Para verificar a influência de variáveis sobre o diagnóstico do HIV e de Tuberculose realizou-se o Weight of Evidence por meio da medida de Information Value. Também utilizou-se o Modelo de classe latente e análise fatorial confirmatória no intuito de verificar a associação das variáveis com os fatores psíquicos, sociodemográficos e comportamentais. Estes procedimentos foram realizados com auxílio dos Softwares SPSS 19.0, Software R e MPLUS. Identificou-se que 79% dos entrevistados apresentavam transtornos mentais comuns, 37% ansiedade leve, 29% desesperança leve, 48% resiliência baixa, 69% depressão; 74% consumiam drogas, 29% positivaram para sífilis, 5% HIV, 1% hepatite B, 75% não utilizam preservativo em todas as relações sexuais, 52% realizam sexo com parceiros casuais, 54% realizam atividade sexual com usuário de droga, 71% sofreu violência física. A análise por meio da Teoria de Resposta ao Item validou como fidedignos para mensurar a vulnerabilidade ao HIV, os seguintes itens: diagnóstico prévio de IST (F=0,473), parceiro com sintoma de IST (F=0,518), uso de droga (F=0,509), sexo em troca de dinheiro (F=0,552), sintomas de IST (F=0,448), quantidade de parceiro sexual (F=0,616), compartilhamento de perfurocortante (F=0,398) e ser vítima se violência sexual (F=0,347). Os fatores identificados pelo Weight of Evidence que influenciam o HIV foram: desesperança,

ansiedade, tempo de moradia na rua, idade, prática de sexo oral. Para a tuberculose encontrou-se: desesperança, resiliência, tempo de moradia na rua, sexo anal e oral. Destacou-se o Modelo de classe latente para três classes com Critério de Informação Akaike de 1828,24, Critério de Informação Bayesiano de 1989,76, entropia relativa de 95,89%, sendo interpretado como aqueles $\leq$30 anos, homens, solteiros, baixa resiliência, depressão, 85% transtorno mental, praticam sexo oral, anal e vaginal, sintomas de ansiedade e depressão leve. A análise fatorial confirmatória encontrou a presença de fatores que influenciam o adoecimento de pessoas em situação de rua, tais como: sociodemográficos, psíquicos e comportamentais, com Índice de Ajuste Comparativo de 0,986; Índice de Tucker-Lewis de 0,982; Raíz Quadrada Média dos Quadrados dos Erros de Aproximação de 0,022; Índice Quadrático Médio 0,075. O estudo permitiu identificar fatores que predispõe ao adoecimento e que podem ser inseridos no atendimento realizado as pessoas em situação de rua de modo a encontrar precocemente fatores que favorecem ao adoecimento, minimizando-os e fornecendo a assistência necessária.

Palavras-chave: Pessoas em situação de rua, Saúde Mental, HIV, Sífilis, Tuberculose.

PATRÍCIO, A. C. F. A. **Clinical conditions associated with homeless people.** Natal, 2019. Thesis, 141 sheets. Thesis (Doctorate in Nursing). Nursing Graduate Program, Federal University of Rio Grande do Norte (RN), 2019.

ABSTRACT

This is a cross-sectional study conducted with 100 homeless people in the city of João Pessoa, Paraíba, with the objective of analyzing clinical conditions associated with homeless people. Data were collected between February and May 2018. For this purpose, a structured interview script, Self-Reporting Questionnaire Scale, Beck Anxiety Scale, Beck Depression Scale, Beck Desperation Scale, Resilience, Hamilton Depression Scale and HIV / AIDS Vulnerability Questionnaire. In addition, rapid tests for HIV, virus, hepatitis B and C were performed. One research was approved by the Ethics Committee on Research on Human Beings of the Federal University of Paraíba, according to opinion number 2456847, CAAE 79486517000005176. Initially, the questionnaire on vulnerability to HIV was validated. HIV, Tuberculosis, and other Sexually Transmitted Infections through Item Response Theory. To verify the influence of variables on the diagnosis of HIV and tuberculosis, perform the Weight of Evidence by measuring the Information Value. The latent class model and confirmatory factor analysis are also used to verify an association of variables with psychological, sociodemographic and behavioral factors. These procedures were performed with the aid of the software SPSS 19.0, Software R and MPLUS. It was found that 79% of respondents have common mental disorders, 37% mild, 29% desperate, 48% low resilience, 69% depression; 74% use drugs, 29% are HIV positive, 5% HIV, 1% hepatitis B, 75% do not use condoms in all sexual relations, 52% have sex with casual partners, 54% have sex with drug users, 71% suffered physical violence. An analysis using the Item Response Theory validated as reliable for measuring vulnerability to HIV, the following items: early diagnosis of STI ($F = 0.473$), partner with STI symptom ($F = 0.518$), drug use ($F = 0.509$), sex for money ($F = 0.552$), STI symptoms ($F = 0.448$), amount of sexual partner ($F = 0.616$), sharpening ($F = 0.398$) and being a victim of sexual violence ($F = 0.347$). Factors considered by the Weight of Evidence that influence HIV were: hopelessness, anxiety, time spent living on the street, age, oral sex. For tuberculosis found: hopelessness, resilience, dwelling time on the street, anal and oral sex. We highlight the Latent Class Model for three classes with Akaike Information Criterion of 1828.24, Bayesian Information Criterion of 1989.76, relative entropy of 95.89%, being interpreted as ≤ 30 years, men, single, low resilience, depression, 85% mental disorder, oral, anal and vaginal sex, anxiety symptoms and mild depression. A confirmatory factor analysis found a presence of factors that influence or harm homeless people, such as: sociodemographic, psychic and behavioral, with a Comparative Adjustment Index of 0.986; Tucker-Lewis Index of 0.982; Mean Square Root of Approximation Error Squares 0.022; Mean Square Index 0.075. The study allowed identifying factors that predispose to postponement and that can be inserted in the care provided as homeless people in order to find early factors that favor the postponement, minimizing them and providing the necessary assistance.

Keywords: Homeless Persons, Mental Health, HIV, Syphilis, Tuberculosis.

LISTA DE ILUSTRAÇÕES

Figura 1. Necessidades humanas básicas comprometidas em pessoas em situação de rua. 32
Quadro 1. Escore dos itens relacionados à vulnerabilidade ao HIV/aids, sífilis e outras IST’s. 38
Quadro 2. Escore referente à vulnerabilidade à tuberculose. 39
Figura 2. Pontos de ausculta respiratória realizada nos voluntários da pesquisa. 40
Figura 3. Leitura dos testes rápidos utilizados nesta pesquisa. 41
Figura 4. Passo a Passo do teste rápido para HIV realizado nos voluntários desta pesquisa. 41
Figura 5. Passo a Passo do teste rápido para sífilis realizado nos voluntários da pesquisa. 42
Figura 6: Etapas para realização do teste de Hepatite B. 43
Figura 7. Passo a Passo do teste rápido de Hepatite C realizado nos voluntários da pesquisa. 43
Figura 8. Etapas da coleta de dados desta pesquisa. 46
Quadro 3. Itens do questionário validado sobre à vulnerabilidade ao HIV/aids, sífilis e outras IST’s 49
Quadro 4. Itens do questionário validado referente à vulnerabilidade à tuberculose 50
Figura 9. Curva Característica do Item do instrumento referente aos comportamentos vulneráveis ao adoecer relacionados à infecção ao HIV, sífilis e outras IST’s de pessoas em situação de rua. João Pessoa, Paraíba, Brasil, 2018. N=100. 63
Figura 10. Curva referente a todos os itens do instrumento de comportamentos vulneráveis ao adoecer relacionados à infecção ao HIV, sífilis e outras IST’s de pessoas em situação de rua. João Pessoa, Paraíba, Brasil, 2018. N=100. 63
Figura 11. Curva Característica do Item do instrumento de vulnerabilidade a tuberculose de pessoas em situação de rua. João Pessoa, Paraíba, Brasil, 2018. N=100. 65
Figura 12. Curva referente a todos os itens do instrumento de vulnerabilidade a tuberculose de pessoas em situação de rua. João Pessoa, Paraíba, Brasil, 2018. N=100. 65
Figura 13. Influência dos fatores psíquicos sobre o HIV de pessoas em situação de rua. João Pessoa, Paraíba, Brasil, 2018. N=100. 67

Figura 14. Influência dos fatores sociodemográficos sobre o HIV de pessoas em situação de rua. João Pessoa, Paraíba, Brasil, 2018. N=100. 68

Figura 15. Influência dos fatores comportamentais sobre o HIV de pessoas em situação de rua. João Pessoa, Paraíba, Brasil, 2018. N=100. 68

Figura 16. Influência dos fatores (psíquicos, sociodemográficos, comportamentais) sobre o HIV de pessoas em situação de rua. João Pessoa, Paraíba, Brasil, 2018. N=100. 69

Figura 17. Influência dos fatores psíquicos sobre a tuberculose de pessoas em situação de rua. João Pessoa, Paraíba, Brasil, 2018. N=100. 69

Figura 18. Influência dos fatores sociodemográficos sobre a tuberculose de pessoas em situação de rua. João Pessoa, Paraíba, Brasil, 2018. N=100. 70

Figura 19. Influência dos fatores comportamentais sobre a tuberculose de pessoas em situação de rua. João Pessoa, Paraíba, Brasil, 2018. N=100. 70

Figura 20. Influência dos fatores (psíquicos, sociodemográficos, comportamentais) sobre a tuberculose de pessoas em situação de rua. João Pessoa, Paraíba, Brasil, 2018. N=100. 71

Figura 21. Dendograma que comprova o agrupamento de fatores psíquicos, sociodemográficos e comportamentais. João Pessoa, Paraíba, Brasil, 2018. N=100. 72

Figura 22. Confirmação da associação das variáveis para suas respectivas categorias através da análise fatorial confirmatória. 74

LISTA DE TABELAS

Tabela 1. Caracterização de pessoas em situação de rua. João Pessoa, Paraíba, Brasil,2018. N=100. ..... **56**

Tabela 2. Distribuição das características relacionadas aos dados clínicos gerais de pessoas em situação de rua. João Pessoa, Paraíba, Brasil, 2018. N=100. ..... **57**

Tabela 3. Testes rápidos e comportamentos vulneráveis ao adoecer relacionados à infecção ao HIV, sífilis e outras IST's de pessoas em situação de rua. João Pessoa, Paraíba, Brasil, 2018. N=100. ..... **58**

Tabela 4. Comportamentos vulneráveis a tuberculose de pessoas em situação de rua. João Pessoa, Paraíba, Brasil, 2018. N=100. ..... **59**

Tabela 5. Resiliência, transtornos mentais comuns, ansiedade, desesperança, depressão e vulnerabilidade ao HIV/aids de pessoas em situação de rua. João Pessoa, Paraíba, Brasil, 2018. N=100. ..... **61**

Tabela 6. Parâmetros da TRI, Carga Fatorial e índices de ajuste da Análise Fatorial para instrumento referente aos comportamentos vulneráveis ao adoecer relacionados à infecção ao HIV, sífilis e outras IST's de pessoas em situação de rua. João Pessoa, Paraíba, Brasil, 2018. N=100. ..... **61**

Tabela 7. Parâmetros da TRI, Carga Fatorial e índices de ajuste da Análise Fatorial para instrumento referente à vulnerabilidade a tuberculose de pessoas em situação de rua. João Pessoa, Paraíba, Brasil, 2018. N=100. ..... **62**

Tabela 8. Teste HIV elaborado com ponto de corte no escore igual a 4 confrontado com o padrão ouro (Teste ELISA) em pessoas em situação de rua. João Pessoa, Paraíba, Brasil, 2018. N=100. ..... **64**

Tabela 9. Propriedades do teste HIV com *cuttoff* igual a 4 para diagnóstico positivo de pessoas em situação de rua. João Pessoa, Paraíba, Brasil, 2018. N=100. ..... **66**

Tabela 10. Teste de Tuberculose elaborado com ponto de corte no escore igual a 3 confrontado com o padrão ouro de pessoas em situação de rua. João Pessoa, Paraíba, Brasil, 2018. N=100. ..... **66**

Tabela 11. Propriedades do teste de Tuberculose com *cuttoff* igual a 3 para diagnóstico positivo de pessoas em situação de rua. João Pessoa, Paraíba, Brasil, 2018. N=100. ..... **67**

Tabela 12. Modelo de classes latentes de pessoas em situação de rua. João Pessoa, Paraíba, Brasil, 2018. N=100. ..... **72**

Tabela 13. Interpretação das classes latentes de pessoas em situação de rua. João Pessoa, Paraíba, Brasil, 2018. N=100. **.. 73**
Tabela 14. Análise fatorial confirmatória referente a categorias de risco à saúde de pessoas em situação de rua. João Pessoa, Paraíba, Brasil, 2018. N=100. **............................... 73**

LISTA DE ABREVIAÇÕES

Pessoa (as) em Situação de Rua (PSR)

Infecções Sexualmente Transmissíveis (IST)

Vírus da Imunodeficiência Humana/Síndrome da Imunodeficiência Adquirida (HIV/aids)

Teoria de Resposta ao Item (TRI)

Centro de Referência Especializado para População em Situação de Rua (CENTRO POP)

Escala Self-Reporting Questionnaire (SRQ 20)

Escala de Desesperança de Beck – BHS

Center of Cognitive Therapy (CCT)

Termo de Consentimento Livre e Esclarecido (TCLE)

Certificado de Apresentação para Apreciação Ética (CAAE)

Raíz Quadrada Média dos Quadrados dos Erros de Aproximação (RMSEA)

Índice Quadrático Médio (RMSR)

Índice de Tucker-Lewis (TLI)

Índice de Ajuste Comparativo (CFI)

Weight of Evidence (WoE)

Information Value (IV)

Odds Ratio (OR)

Modelo de classe latente (Latent Class model = LCA)

Critério de Informação Akaike (AIC)

Critério de Informação Bayesiano (BIC)

Razão de verossimilhança (G^2)

Tuberculose (TB)

Unidades Básicas de Saúde (UBS)

Sistema Único de Saúde (SUS)

SUMÁRIO

1 INTRODUÇÃO **18**
2 OBJETIVOS **22**
2.1 OBJETIVO GERAL 22
2.2 OBJETIVOS ESPECÍFICOS 22
3 HIPÓTESES **23**
4 REVISÃO DE LITERATURA **24**
4.1 COMPORTAMENTOS E CONDIÇÕES VULNERÁVEIS AO HIV/AIDS E OUTRAS INFECÇÕES SEXUALMENTE TRANSMISSÍVEIS (IST) DE PESSOAS EM SITUAÇÃO DE RUA 24
4.2 VULNERABILIDADE À TUBERCULOSE EM PESSOAS EM SITUAÇÃO DE RUA 27
4.3 RESILIÊNCIA DE PESSOAS EM SITUAÇÃO DE RUA 28
4.4 TRANSTORNO MENTAL COMUM EM PESSOAS EM SITUAÇÃO DE RUA 28
4.5 ANSIEDADE, DEPRESSÃO E DESESPERANÇA DE PESSOAS EM SITUAÇÃO DE RUA 29
4.6 MASLOW E A TEORIA DAS NECESSIDADES HUMANAS BÁSICAS APLICADA A POPULAÇÃO DE RUA: REFERENCIAL FILOSÓFICO 31
4.7 TEORIA DE RESPOSTA AO ITEM 33
5 MÉTODO 34
5.1 TIPO DE ESTUDO E LOCAL DE COLETA DE DADOS 34
5.2 LOCAL DE COLETA DE DADOS 34
5.3 POPULAÇÃO E AMOSTRA 35
5.4 CRITÉRIOS DE INCLUSÃO E EXCLUSÃO 36
5.5 INSTRUMENTOS DE COLETA DE DADOS 36
5.5.1 Instrumento sociodemográfico 36
5.5.1.1 Dados Clínicos Gerais 37
5.5.1.2 Vulnerabilidade ao adoecer: HIV/aids, sífilis, IST’s 37
5.5.1.3 Questionamentos/comportamentos quanto a vulnerabilidade à Tuberculose 39
5.5.1.4 Testes rápidos para HIV, sífilis, hepatite B, hepatite C 40
5.5.2 INSTRUMENTO: ESCALA SRQ 20 44

5.5.3 INSTRUMENTO: ESCALA DE ANSIEDADE DE BECK - BAI 44
5.5.4 INSTRUMENTO: ESCALA DE DEPRESSÃO DE BECK –BDI 44
5.5.5 INSTRUMENTO: ESCALA DE DESESPERANÇA DE BECK - BHS 45
5.5.6 INSTRUMENTO: ESCALA DE RESILIÊNCIA..45
5.5.7 INSTRUMENTO: ESCALA DE DEPRESSÃO DE HAMILTON..45
5.5.8 INSTRUMENTO: QUESTIONÁRIO DE VULNERABILIDADE AO HIV/AIDS 46
5.6 PROCEDIMENTOS DE COLETA DOS DADOS...46
5.6.1 EQUIPE DE COLETA DOS DADOS 47
5.6.2 EQUIPAMENTOS DE COLETA DOS DADOS 48
5.7 ÉTICA DA PESQUISA 48
5.8 PROCEDIMENTOS DE ANÁLISE DOS DADOS 49
5.9 RISCOS 54
6 RESULTADOS 56
6 .1 Características sociodemográficas e clínicas...56
6 .2 Saúde mental/fatores pesíquicos...58
6 .3 Testes rápidos, comportamentos e fatores que predispõem ao HIV, IST’s e tuberculose...59
7 DISCUSSÃO 75
7 .1 Características sociodemográficas e clínicas...75
7 .2 Saúde mental/fatores pesíquicos...77
7 .3 Testes rápidos, comportamentos e fatores que predispõem ao HIV, IST’s e tuberculose...79
8 **CONCLUSÃO**...**85**
REFERÊNCIAS 87
APÊNDICE E ANEXOS...105

1 INTRODUÇÃO

As pessoas em Situação de Rua (PSR) são compreendidas como um grupo populacional heterogêneo, caracterizado por condição de pobreza extrema, pela interrupção ou fragilidade de vínculos familiares e falta de moradia convencional regular (BRASIL,2008; ROY *et al*., 2016).

No mundo, estima-se que 100 milhões de pessoas estejam desabrigadas (ONU, 2018) No Brasil, não há estimativas concretas do quantitativo de pessoas em situação de rua, no entanto, o Instituto de Pesquisa Econômica Aplicada (2016) através do modelo analítico determinou um total de 122.890 pessoas.

A mortalidade de pessoas em situação de rua é maior quando comparados a população geral, sendo oito vezes maior em homens e 12 vezes maior do que mulheres (LUCHENSKI, 2018).

A população de rua encontra-se excluída das estruturas convencionais da sociedade, resultando no comprometimento de sua saúde física e psíquica. A PSR busca neste cenário o acolhimento, entretanto, deparam-se com situações e comportamentos de risco, entre estes a exposição a chuva, doenças parasitárias e infectocontagiosas, práticas sexuais desprotegidas, uso de drogas, obesidade, alimentação precária, alterações bucais, dentre outros (SILVA *et al*., 2014).

Desta forma, verifica-se que as PSR se encontram vulneráveis a diversas doenças. Tratando-se de vulnerabilidade cabe destacar que esta compreende um sentido multidimensional que envolve fragilidades, seja ela social, ambiental, política, civil, educacional ou relacionada à saúde. Sendo assim, Ayres (2009) afirma que a vulnerabilidade é composta por três modalidades: individual, social e programática. O primeiro refere-se a questões cognitivas e comportamentais, a segunda contempla o acesso a informação e barreiras culturais, o terceiro abrange questões políticas. Ressalta-se que quanto mais estas dimensões estão afetando o indivíduo, maior será a sua vulnerabilidade ao adoecer.

As condições de saúde de PSR são precárias, apresentam maior prevalência de doenças quando comparados a população geral, como transtornos mentais (GEDDS; FAZEL, 2011) e doenças infecciosas. Apresentam ainda, prática sexual precoce e muitas vezes desprotegida, aumentando o risco de Infecções Sexualmente Transmissíveis (IST), além de possuírem acesso à saúde fragilizado (VERULAVA *et al*., 2018).

Estudo realizado em Tbilisi/Geórgia apontou que 98% das PSR ouviu falar sobre IST's, sendo o Vírus da Imunodeficiência Humana/Síndrome da Imunodeficiência Adquirida

(HIV/aids) com maior prevalência, seguido de hepatite B, hepatite C e sífilis. A maior parte dos entrevistados conheciam os sintomas de IST's e entendiam que o uso do preservativo pode reduzir o risco de infecção. Entretanto, mais de 20% discordaram da necessidade do uso do preservativo durante a relação sexual anal e 68,3% afirmaram que o quantitativo de parceiros não interfere nas chances de ter IST's. Embora, tenham ouvido falar, não sabem de fato o que é a doença e não apresentaram boa conscientização (VERULAVA *et al*., 2018).

Pesquisa de meta-análise demonstrou que houve maior prevalência de Hepatite C e menor índice de tuberculose em pessoas em situação de rua. Além disso, houve associação estatisticamente significativa do aumento da idade com a prevalência de HIV/aids (BEIJER, 2012).

Segundo o Boletim Epidemiológico (2018) entre 2007 e junho de 2018 foram identificados 117.415 casos de HIV no Brasil e as PSR são uma população chave caracterizada por comportamentos vulneráveis a infecção.

Os determinantes que contribuem para o aumento da vulnerabilidade a infecção ao HIV/aids em PSR incluem: conhecimento, crenças e atitudes erradas sobre o HIV, redes de apoio, rejeição e discriminação, a frequência de uso de drogas e de preservativos (FERNANDEZ *et al*., 2017).

A presença de HIV e hepatite é significativamente maior entre PSR que são usuárias de crack. Este fato pode ser correlacionado a redução da imunidade, menor acesso a ações preventivas, maior possibilidade de contato com outros enfermos que vivem em locais inapropriados aumentando também a chance de contaminação por outras doenças, como a Tuberculose (HALPERN *et al*., 2017).

A sífilis está relacionada ao sexo desprotegido e potencializa a transmissão do HIV, apresenta prevalência elevada em PSR. Este fato envolve número de parceiro sexual, sexo anal sem preservativo (PINTO *et al*., 2014).

Considera-se que as PSR apresentam dificuldade de prevenção de certos agravos, como Infecções Sexualmente Transmissíveis (IST's), dengue, leptospirose, tuberculose, dermatoses, além de agravos relacionados a saúde bucal e mental, ruptura de vínculos familiares (BRASIL,2012a).

As PSR encontram-se mais vulneráveis a vários fatores de risco, representam uma preocupação por parte da gestão afim de garantir direitos básicos quanto indivíduo como estabelece a Política Nacional para População em situação de Rua instituída no Decreto Lei nº 7.053 de 2009 e pela Declaração Nacional dos Direitos Humanos (BRASIL, 2009).

A tuberculose apresenta-se em maior risco em indivíduos desabrigados, sendo influenciado pelo uso de drogas como o álcool e tabaco, precariedade social e histórico de penitenciária, características de PSR (ZENNER *et al*., 2013).

Estudo realizado no Canadá com 61 mulheres em situação de rua destaca que a procura dos serviços de saúde pode ser potencializada na presença de entendimento, compaixão e ausência de julgamentos por parte dos profissionais, pois faz com que esta população sinta-se confortável para voltar quando necessário. Além disso, a violência sexual, verbal, financeira, emocional e física está presente no cotidiano destas mulheres que submetem-se a relacionamentos não saudáveis e ao uso de substâncias ilícitas para suportar o estresse. Ressalta-se problemas relacionados a saúde mental, depressão, ansiedade, insônia, raiva, sofrimento, desespero, solidão e agorafobia que consiste na perturbação da ansiedade em situações inseguras (SCHMIDT *et al*., 2015).

As PSR jovem apresentam maior uso de drogas injetáveis, influenciado pelos colegas e parceiros sexuais, exposição/facilidade de acesso as substâncias, dificuldade de acesso ao tratamento para drogas, colocando-os no quadro de vulnerabilidade a infecções transmissíveis, como o HIV, sífilis, hepatite e tuberculose (DELL *et al*., 2013).

Dentre as drogas utilizadas em pesquisa realizada com 47 pessoas em situação de rua em Vancouver/Canadá, predominou o tabaco, álcool, maconha, êxtase, cocaína e crack. Alerta-se para os fatores que podem proporcionar tardiamento no uso de drogas como: medo (de agulhas, vício), autoestima, estigma, suporte/inclusão (apoio de familiares), uso de drogas por familiares (uso familiar de drogas), amigos e responsabilidades por outras pessoas, espaços seguros/envolventes (TIOZER *et al*., 2015).

As pessoas que apresentam resistência a comportamentos vulneráveis podem ser caracterizadas como resilientes que pode ser entendida como a capacidade da pessoa equilibrar, reconhecer, negociar, enfrentar, resistir, superar situações negativas de forma eficaz com estratégias e recursos de enfrentamento que possam proporcionar bem estar e esperança de dias melhores (RUIZ-BARQUÍN *et al*., 2015).

O indivíduo resiliente é aquele que reconhece a dor, percebe seu sentido e tolera-a até resolver os conflitos envolvidos, podendo ser influenciado pelo meio ambiente uma vez que ao sentir-se amparado e acolhido aumentará as chances de superação, além dos fatores individuais e acontecimentos ao longo da vida (CRUZ *et al.*, 2018).

Além disso, as PSR enfrentam diversas dificuldades que contribuem para ansiedade e estresse, potencializando comprometimento na saúde mental, como demonstrado em pesquisa com 16.912 indivíduos sem abrigo (SZYMKOWIAK *et al*., 2017). O sofrimento mental

constitui um motivo de abandono da casa, encontrando estas pessoas a rua como um local acolhedor, tranquilo e sem julgamentos (VILLA *et al*., 2017).

Sendo assim, os determinantes sociais de saúde, que consistem naqueles que afetam a saúde dos indivíduos, no contexto das pessoas em situação de rua apresentam-se comprometidos, seja no fator ambiental (condições de habitação, transporte, poluição do ar, entre outros), econômico (emprego, ocupação, entre outros) ou social (estilo de vida, inclusão social, comportamentos relacionados à saúde, educação, entre outros) (CARRAPATO *et al*., 2017).

Destaca-se que esse estudo justifica-se pela precariedade de assistência a saúde direcionada a pessoas em situação de rua, fragilidade de políticas públicas, estimativa incerta do quantitativo de PSR, apoio social carente, discriminação, estigma, maior exposição a comportamentos vulneráveis. Sendo assim, a enfermagem como profissão ética e atuante na equipe multiprofissional dos Centros de Atendimento, Acolhimento e Casas de Apoio de PSR pode investigar fatores relacionados a saúde e doença destas pessoas, especificamente sobre saúde mental, HIV, sífilis, tuberculose, contribuindo de forma social para redução de agravos relacionados a fragilidades oriundas deste grupo populacional.

Considerando que as condições de vulnerabilidade vivenciadas pela população em situação de rua envolvem questões psicossociais geradoras de sofrimentos físicos e emocionais, possibilitam vulnerabilidades à saúde, representa-se como estratégia ímpar fornecer assistência e cuidado a saúde, na perspectiva da detecção precoce de agravos e prevenção de doenças.

Soma-se a isso a preocupação, experiências anteriores com pesquisas envolvendo populações mais carentes e menos favorecidas, além da curiosidade em descobrir fatores que contribuem para a vulnerabilidade em PSR, na perspectiva de traçar estratégias de enfrentamento.

Nesta perspectiva, emergiu o seguinte questionamento: Quais são as condições clínicas associadas às pessoas em situação de rua?

2 OBJETIVOS

2.1 OBJETIVO GERAL

Analisar condições clínicas associadas às pessoas em situação de rua.

2.2 OBJETIVOS ESPECÍFICOS

- Caracterizar pessoas em situação de rua quanto as variáveis: gênero, idade, estado civil/presença de companheiro, filhos, religião, tempo e motivo de moradia na rua, procura periódica por serviço de saúde, doenças prévias, manifestações clínicas, uso de medicamentos, drogas ilícitas;
- Identificar a presença de: depressão, ansiedade, transtornos mentais comuns, desesperança, resiliência, HIV, sífilis, hepatite b e c de pessoas em situação de rua;
- Validar por meio da Teoria de Resposta ao Item os questionários sobre vulnerabilidade ao HIV, sífilis e outras Infecções Sexualmente Transmissíveis, bem como Tuberculose de pessoas em situação de rua;
- Averiguar comportamentos vulneráveis a infecção ao HIV, sífilis e outras Infecções Sexualmente Transmissíveis, bem como Tuberculose de pessoas em situação de rua;
- Verificar a influência de fatores psíquicos, sociodemográficos e comportamentais sobre o HIV e a tuberculose.

3 HIPÓTESES

- As pessoas em situação de rua apresentam comportamentos vulneráveis a: HIV, IST’s, sífilis, tuberculose, hepatite b e c;
- Existe associação dos fatores psíquicos, sociodemográficos e comportamentais sobre o HIV e a tuberculose;
- A depressão, ansiedade, transtornos mentais, resiliência e desesperança estão comprometidas em pessoas em situação de rua;
- Os itens construídos que contemplam o questionário de vulnerabilidade ao HIV e a tuberculose de pessoas em situação de rua são válidos por meio da Teoria de Resposta ao Item.

4 REVISÃO DE LITERATURA

Esta pesquisa irá enfatizar as condições clínicas vulneráveis ao adoecer de pessoas em situação de rua. Desta forma, abordou-se na revisão da literatura os seguintes temas: comportamentos e condições vulneráveis ao HIV/aids e outras Infecções Sexualmente Transmissíveis (IST); comportamentos e condições clínicas vulneráveis à tuberculose; resiliência; transtorno mental comum; ansiedade; desesperança e depressão; Maslow e a teoria das necessidades humanas básicas.

4.1 COMPORTAMENTOS E CONDIÇÕES VULNERÁVEIS AO HIV/AIDS E OUTRAS INFECÇÕES SEXUALMENTE TRANSMISSÍVEIS (IST'S) DE PESSOAS EM SITUAÇÃO DE RUA

Vulnerabilidade pode ser entendida como algo susceptível a desencadear perigo ou risco, pode ser dividida em três dimensões individual, social e programática. Ao ferir ou fragilizar estas subdivisões o indivíduo pode apresentar maior possibilidade de adquirir ou transmitir determinada doença (AYRES *et al.*, 2008; AYRES *et al.*, 2009).

A vulnerabilidade envolve dimensões interligadas que abrangem aspectos individuais, sociais e programáticos. A dimensão individual depende do grau e da qualidade da informação de que os indivíduos dispõem sobre o problema; a dimensão social pode ser entendida como um espelho das condições de bem-estar social e a programática pode ser ponderada a partir do compromisso das autoridades com o enfrentamento do problema. Essas dimensões são interdependentes e entrelaçam o indivíduo e a coletividade. O sentido do termo vulnerabilidade engloba a chance de exposição ao adoecimento (AYRES,2012).

A vulnerabilidade social é nítida no contexto das PSR, pois possuem menos suporte social e pouco acesso a serviços de saúde, ações de prevenção e apoio social. Desta forma, podem não ter suas necessidades atendidas, sendo necessário incluí-los nas ações prioritárias do Ministério da Saúde (ARGINTARU *et al.*, 2013; BRASIL, 2014).

A percepção de que a PSR é um fracasso, transgressão, apresentam comportamentos violentos, roubos, sujeiras e doenças permeia o contidiano da população em geral. Entretanto, a rua foi o caminho encontrado para o alívio de sentimentos de dor, exclusão e angústia, além da pobreza extrema, falta de acesso a escolaridade e saúde. Sendo assim, percebe-se a vulnerabilidade social imperando no dia dia da PSR, gerando mais possibilidades de

comportamentos e práticas inseguras que podem desencadear ou transmitir doenças, como HIV, hepatite, sífilis, tuberculose (RUI, 2014; MEDEIROS, 2015).

Destaca-se que o uso de drogas apresenta associação com o HIV, a tuberculose e a hepatite, além de transtornos psicológicos como a depressão e ideação suicida (STRAATEN et. al., 2014). Estudo realizado com 244 PSR detectou prevalência de HIV em 6,56% estando atrelado ao maior uso de cocaína e crack, entretanto estes indivíduos relataram procurar serviços de saúde de atenção primária. (PARKER; DYKEMA, 2014).

Grande parte das pessoas em situação de rua praticam comportamentos que as tornam mais vulneráveis ao adoecer, sabe-se que as condições que vivem não são as ideais e quando associadas a atitudes vulneráveis a possibilidade de adoecimento aumenta consideravelmente. Comportamentos como: uso de drogas, compartilhamento de seringas, venda do sexo/sobrevivência, sexo desprotegido, multiplicidade de parceiros, início da vida sexual precoce (SKEVINGTON *et al*., 2013; AMIRI, 2014).

As pessoas em situação de rua iniciam o uso de drogas injetáveis na adolescência, colocando-os na dependência e aumentando o risco de Infecções Transmissíveis. O consumo de drogas injetáveis pelos jovens em situação de rua atinge entre 20% e 50%, possuem 11 vezes mais chances de morrerem de overdose e suicídio quando comparados a população geral (DELL *et al*., 2013; WERB *et al*., 2013).

Estudo realizado com 593 pessoas em situação de rua demonstrou que 33,19% usam preservativo, 52,46% compartilham agulhas, 17,5% das mulheres vendem o sexo (AMIRI *et al*., 2018).

O início precoce da atividade sexual também consiste em um fator de vulnerabilidade ao HIV, assim como destacado em pesquisa com pessoas em situação de rua em que 71% dos voluntários iniciaram a vida sexual entre os 14 e 18 anos. Detalhe importante é que nesta mesma pesquisa 53% dos participantes não utilizam preservativo, mas 89% das PSR que participaram não percebem que realizam comportamentos de risco para o HIV (MARIA *et al*., 2018).

Existem estratégias para reduzir a negligência do uso do preservativo devido ao consumo de drogas, como: a injeção supervisionada e programas de trocas de seringas, além de testagens periódicas para HIV e tratamento ampliado (MARSALL *et al*, 2009).

O uso de drogas torna-se comum entre pessoas que vivem em situação de rua, sendo justificado pelo auxílio no esquecimento de estar em um ambiente que envolve solidão, risco de doenças, depressão, estresse (APTEKAR; STOECKLIN, 2014).

O crack embora não seja a droga ilícita mais utilizada, constitui um problema de saúde pública, principalmente entre as PSR, sendo responsável por diversas internações em hospitais psiquiátricos, gerando custos financeiros para o Sistema Público de Saúde (HALPERN *et al*., 2017).

Pesquisa realizada na Espanha verificou os atendimentos hospitalares destinados a PSR, revelando 27% das internações por doenças mentais, 19,6% infecciosos e 18,4% respiratórias (PATRICIO *et al*., 2016).

Quanto a sífilis, dentre os tipos adquirida, em gestante e congênita há um total de 193.479 casos notificados no ano 2017, sendo 31.255 no Nordeste. Ao analisar a taxa de detecção de sífilis entre os anos 2010 e 2017 encontra-se um aumento consideravel na taxa do tipo de sífilis adquirida de 2% para 58,1% (BRASILb, 2018).

A sífilis é uma doença infecciosa que pode ser transmitida por contato sexual e sua propagação está relacionada a fatores sociais, biológicos, comportamentais (KENT; ROMANELLI, 2008). Estudo com pessoas sem-teto, de São Paulo,Brasil, revelou que dentre os 1389 indivíduos que foram submetidos ao teste rápido, 13% reveleram positividade para sífilis (PINTO *et al*., 2014).

Ressalta-se que as hepatites virais no Brasil, no período de 1999 a 2018, atingiu mais de 600 mil pessoas, sendo o Nordeste com maior prevalência de hepatite A, no Sudestes hepeatites B e C e no Norte do país a hepatite D (BRASILa, 2019).

Destaca-se ainda, que, no geral, as hepatites podem ser transmitidas de diversas formas, como contágio fecal-oral, sexo desprotegido, contato com sangue de pessoas contaminadas, vertical de mãe para filho. No Brasil há testes rápidos para hepatites B e C onde facilitam o diagnóstico, sendo no Brasil a Hepatite C responsável por mais de 70% dos óbitos relacionados a todas as formas desta doença (BRASILb, 2019).

Desta forma, as PSR estão propensas ao adoecimento, uma vez que, grande parte destas pessoas, apresentam prática sexual insegura e compartilham objetos perfurocortantes para uso de drogas (BEECH *et al*., 2003; BRITO *et al*., 2007).

Nesta perspectiva, dentre os comportamentos vulneráveis ao HIV/aids e outras IST's incluem prática sexual insegura sem o uso do preservativo, múltiplos parceiros sexuais, uso de drogas injetáveis ou não, sexo anal apresenta maior chance de transmissibilidade, sexo em período menstrual, compartilhamento de aparelhos perfurocortantes, conhecimento frágil sobre a temática, histórico prévio de outras IST's, condição socioeconômica, política pública insuficiente, acesso prejudicado ao serviços de saúde (SANTOS *et al*., 2017).

4.2 VULNERABILIDADE À TUBERCULOSE EM PESSOAS EM SITUAÇÃO DE RUA

A notificação de tuberculose no Brasil, no ano 2017 atingiu 69.569 casos novos com coeficiente de incidência de 33,5casos/100mil habitantes, com redução da incidência se comparado com o ano 2008 que atingia 39/100mil habitantes (BRASILc, 2018). O Brasil faz parte dos 22 países que priorizam ações e estratégias para a tuberculose, estando em 16ª posição (BRASIL, 2015). No Brasil esta doença apresenta prevalência 37 a 60 vezes maior em pessoas em situaçao de rua quando comparados a população geral (BRASIL, 2014).

A tuberculose concentra-se em populações mais vulneráveis como os sem-teto, consumidores de drogas e álcool, presidiários, pessoas vivendo com HIV/aids (ECDC, 2016; TOMS *et al*., 2015). A Organização Mundial de Saúde revela aumento considerável de casos de tuberculose nos usuários de drogas injetáveis e no consumo excessivo de álcool (FIGUEROA-MUNOZ; RAMON-PARDO, 2008).

Estudo realizado em Teerã/Irã com 593 PSR identificou a presença de tuberculose latente em 50% da amostra, correndo risco de desenvolver a forma clínica da Tuberculose, devido ao aglomerado de pessoas, má condição de habitação e alimentação, abuso de drogas. Nota-se a importância de monitorar estes fatores de risco, além de prevenir e tratar a tuberculose, pois também há o risco de transmissibilidade em outras camadas da sociedade (AMARI *et al*., 2014).

É importante destacar que a presença da infecção pelo HIV/aids aumenta as chances de tuberculose, mas sabe-se que o diagnóstico de tuberculose pode ser um indicador da infecção pelo HIV. Nas pessoas em situação de rua a tuberculose é quatro vezes mais letal quando comparado a população geral (AMIRI *et al*., 2014).

Para a tuberculose destacam-se ainda as condutas vulneráveis: virose recorrente, sintomáticos respiratórios (tosse, febre, sudorese), tabagismo, alcoolismo, contato com indivíduo infectado (ERIKSEN, 2015; WHO, 2014; NIKOVSKA; TOZIJA, 2014).

O tratamento para em pessoas em situação de rua com tuberculose apresenta diversas dificuldades, dentre elas o uso de drogas, sendo fundamental que no plano de cuidados estabeleça-se estratégias para conscientizar a importância do esquema completo, isto não significa impor a abstinência, mas sim, trabalhar com a redução de danos (ZUIM; TRAJMAN, 2018).

4.3 RESILIÊNCIA DE PESSOAS EM SITUAÇÃO DE RUA

A resiliência está relacionada a capacidade de resistir a situações estressoras, superar adversidades sem que ocorra danos psicológicos ou físicos, além de desencadear bem-estar pessoal (HILLS *et al.*, 2016).

Diante da exposição a adversidades de origem psicológica e ambiental, a capacidade de resistir e inclinar-se à saúde, buscando o bem-estar, sendo a família, sociedade e cultura recursos importantes para superação (UNGAR, 2008; UNGAR, 2011).

As relações sociais estabelecidas entre pessoas em situação de rua e amigos encorajam e contribuem para a resiliência (HILLS *et al.*, 2018). O dia a dia nas ruas é permeado por grandes obstáculos, entretanto, muitas pessoas em situação de rua conseguem adaptar-se e encontrar meios de superação, demonstrando resiliência (ROOK, 2015).

Contudo, pesquisa revela que estar em condição de pessoa em situação de rua contribui para redução da resiliência (MABHALA *et al.*, 2017). Ressalta-se que os principais fatores que contribuem para resiliência em pessoas em situação de rua, são: serviços de saúde, recursos psicológicos, relações sociais, perseverança, autoestima, autonomia, fé, otimismo, atividade física. E, aqueles que fragilizam a resiliência, são: traumas, pobreza, exclusão social, violência, falta de moradia, consumo de drogas (ANTUNES *et al.*, 2016; MATOS, 2016; NASCIMENTO, 2018; CAMPOS; CUNHA, 2016; RESENDE, 2015; HALPERN *et al.*, 2017; MORERA; PADILHA, 2015).

Entretanto, há aqueles que a medida que o tempo se prolonga nas ruas, encontram alternativas de enfrentamento através da troca de experiências e dos vínculos construídos com outras pessoas em situação de rua que encontram-se na mesma condição. Desta forma, descobrem formas de sobrevivência, estabelecem vínculos afetivos e passam a ter a rua como referência (BRASIL, 2011).

4.4 TRANSTORNO MENTAL COMUM EM PESSOAS EM SITUAÇÃO DE RUA

As PSR estão mais susceptíveis a apresentarem transtornos mentais devido as condições em que vivem, sendo que pesquisa realizada em Paris, França, com esta população demonstrou variação dos tipos de transtornos mentais relacionados ao gênero, sendo a ansiedade e depressão mais frequente nas mulheres. Os vícios, como consumo de álcool e drogas apresentavam maior predomínio de homens (LAPORTE *et al.*, 2018).

Os transtornos psiquiátricos são 10 vezes mais prevalentes e o transtorno do humor quatro vezes nas pessoas em situação de rua quando comparado a população geral (LAPORTE *et al*., 2018).

Desta forma, não é novidade que as pessoas em situação de rua apresentam mais chances de desenvolverem doenças mentais, influenciadas pelas condições de vulnerabilidade em que vivem. Estudo realizado com esta população evidenciou a presença de diagnósticos de saúde mental com 13,4% para transtorno de ajuste (caracterizado por sintomas emocionais ou comportamentais em resposta a um estressor ou estressores identificáveis ocorrendo dentro de três meses do início do estressor ou estressores), hiperatividade em 10,4%, transtorno de ansiedade 22,4%, transtorno do humor 17,9% e transtorno por uso de substância em 19,4% das pessoas (SAPERSTEIN *et al*., 2014).

Dentre as alternativas para minimização do sofrimento mental as pessoas em situação de rua adotam animais de estimação na perspectiva de adquirir um companheiro para as atividades do dia dia, bem como, receber afeto. No entanto, isto pode afetar o acesso aos serviços sociais que abrigam estas pessoas, pois em sua grande maioria é proibida a entrada de animais de estimação. As pessoas em situação de rua preferem dormir nas ruas do que ter que abandonar seus animais de estimação para ser aceito em algum abrigo. A PSR encontra nos seus animais de estimação apoio emocional, aceitação, conforto, solidão reduzida, isolamento social diminuído, sendo muitas vezes a única fonte de companhia, amor, motivação e proteção (RHOADES *et al*., 2015).

Tratando-se de transtorno mental cabe destacar que embora seja mais prevalente nas pessoas em situação de rua, estas possuem acesso fragilizado aos sistemas de saúde e apoio psicosocial, seja por ausência de documentos, preparo da equipe profissional, prioridades devido a necessidade de sobrevivência, dificuldade de encaminhamento, indisponibilidade de vagas ou por desconhecimento dos serviços oferecidos pelas políticas públicas de saúde (RHOADES *et al*., 2014).

4.5 ANSIEDADE, DEPRESSÃO E DESESPERANÇA DE PESSOAS EM SITUAÇÃO DE RUA

A doença mental em pessoas em situação de rua torna-se cada vez mais prevalente, necessitando muitas vezes de intervenção hospitalar para internação durante a crise. Estas internações apresentam como características: longo período e ônus maior quando comparado a população geral, sendo justificado pelas condições/comprometimento de saúde geral que

estas pessoas apresentam que por sua vez pode ser atrelado a fragilidade no acesso aos serviços de saúde na atenção primária (FAZEL *et al*., 2014).

Ressalta-se que em grande parte dos casos a doença mental ocorre anteriormente a situação de falta de moradia, mas seu acontecimento potencializa-a (LAPAROTE *et al*., 2018).

Dentre os vários comprometimentos relacionados à saúde de pessoas em situação de rua, encontra-se a ansiedade que pode desencadear comportamentos vulneráveis como o consumo de drogas e depressão, reduzindo a esperança de uma vida melhor (BONUGLI, 2013).

Estudo de metanálise realizada no ano 2017 encontrou dentre diversos tipos de transtornos mentais, prevalência de 17,6% de transtorno de ansiedade em pessoas em situação de rua (SCHREITER *et al*., 2017). Pesquisa revelou que a presença de ansiedade em pessoas em situação de rua encontra-se mais prevalente nas mulheres (LAPORTE *et al*., 2018).

No que concerne a depressão em pessoas em situação de rua estudo aponta que esta é quatro vezes maior quando comparada a população geral. Ressalta-se que o apoio social reduz pensamentos suicidas e sofrimentos, demonstrando que serviços de assistência social é essencial no combate a depressão (YIM *et al*., 2015).

As doenças mentais ansiedade e depressão são mais prevalentes nas PSR quando comparados com a população geral (DUNNER *et al*., 2012). Fatores biológicos, psicológicos, sociais, ansiedade e de desenvolvimento explicam a alta prevalência de sofrimento mental e uso de drogas nesta população (STRAATEN *et al*., 2014).

As doenças psicóticas e a depressão estão prevalentes entre 2,8% e 42,3% nas PSR, no entanto, torna-se preocupante que apenas 13% recebem tratamento para transtornos mentais, expondo as barreiras na assistência à saúde, englobando aspectos situacionais, financeiros, falta de eficácia percebida, experiência negativa com o serviço de saúde mental (YIM *et al*., 2015).

Apesar de tantos acometimentos, há pessoas em situação de rua que acreditam em dias melhores e possuem esperança de encontrar os familiares e reerguer-se na vida conquistando emprego e moradia. Entretanto, a desesperança também permeia este contexto, gerando sentimento de revolta, desconfiança dos colegas, déficit de autocuidado, suicídio e doença mental (BISCOTTO *et al*., 2016).

4.6 MASLOW E A TEORIA DAS NECESSIDADES HUMANAS BÁSICAS APLICADA A POPULAÇÃO DE RUA: REFERENCIAL FILOSÓFICO

A Teoria das Necessidades Humanas Básicas, instituída pelo psicólogo Abraham Maslow (1908-1970), afirma que a necessidade fisiológica (alimentação, água, entre outros) é a mais essencial e encontra-se na base, mas defende que a medida que um nível de necessidade é atendido, o próximo torna-se dominante (MASLOW,1943; HENRICK, 2010).

As pessoas em situação de rua estão expostas constantemente a violência, discriminação, privação do sono, fome, pobreza extrema. Além de serem estigmatizados como bandidos, vagabundos, viciados e perigosos (SOUSA, 2009). Apresentam suas necessidades humanas básicas fragilizadas desde realização pessoal, estima, amor/relacionamento, segurança às questões fisiológicas. Quando as necessidades humanas básicas estão satisfeitas ou ao menos equilibradas, há uma forte tendência à superação/redução da incidência de transtornos mentais.

A realização pessoal engloba itens relacionados a moralidade, criatividade, ausência de preconceito, aceitação dos fatos. Sendo assim, as pessoas em situação de rua apresentam fragilidades neste aspecto, uma vez que sofrem julgamentos e preconceitos diariamente (MASLOW,1943).

A estima relaciona-se a autoestima, confiança, respeito, sendo todos estes itens afetados na pessoa em situação de rua, pois a grande maioria sente-se desrespeitado pela população geral, órgãos públicos e profissionais, pois não possuem acesso adequado aos meios de transporte, local apropriado para realizar as necessidades fisiológicas, emprego fixo, dificuldade de acesso aos serviços de saúde, sendo este uma violação dos direitos humanos fundamentais (MASLOW,1943).

No aspecto referente ao amor/relacionamento também há comprometimento, uma vez que ter amigos torna-se difícil no ambiente das ruas e há privação/redução da convivência com a família (MASLOW,1943). Além disso, a intimidade sexual é limitada, trazendo esta prática para os espaços públicos, muitas vezes ocorrendo o sexo inseguro. O contexto das pessoas em situação de rua é marcado por rupturas familiares, muitas vezes decorrente do uso de substâncias psicoativas.

A segurança envolve os aspectos a respeito da segurança do corpo, emprego, saúde, moradia (MASLOW,1943). Todos estes fatores encontram-se comprometidos nas pessoas em situação de rua que dormem e vivem em qualquer lugar, sem a mínima segurança, sem saber se acordarão vivos, se serão vítimas da violência. Permeiam ambientes insalubres e estão

expostos a maior vulnerabilidade, potencializando as chances de infecções, parasitoses, entre outros.

As necessidades fisiológicas de alimento, sono, repouso apresentam-se afetadas neste grupo populacional pois não possuem privacidade para realizá-las, alguns procuram albergues ou banheiros públicos, mas a maior parte utiliza as ruas (MASLOW,1943).

Desta forma, as pessoas em situação de rua possuem privação de seus direitos básicos: moradia, saúde, educação, lazer, trabalho e segurança.

Pesquisa realizada em Minas Gerais descreveu relatos de pessoas em situação de rua explicitando sofrerem violência e o constante medo de morrer. Ainda, destacam que passam fome e sentem dificuldade de serem atendidos por estabelecimentos de saúde devido à falta de documentos de identificação. Revelam a crença e a fé como fontes de motivação. Ressaltam que quando são atendidos no hospital e recebem alta deveria existir uma equipe de saúde para cuidar diariamente deles nas ruas (OLIVEIRA, 2018).

Considerando os aspectos supracitados, a Figura 1 representa de qual modo, as necessidades humanas básicas das PSR estão fragilizadas.

Figura 1 – Necessidades humanas básicas comprometidas em pessoas em situação de rua.

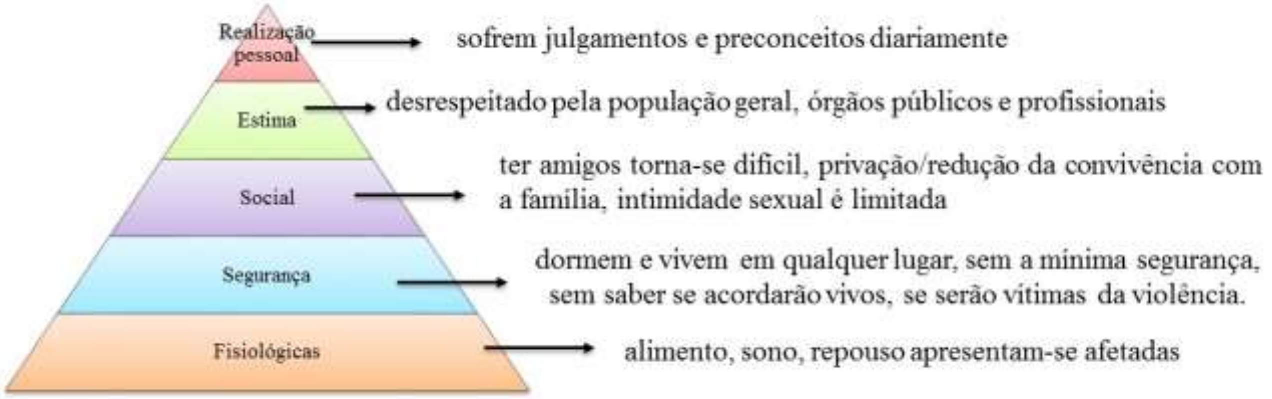

Fonte: da Pesquisadora, 2019.

4.7 TEORIA DE RESPOSTA AO ITEM

A Teoria de Resposta ao Item (TRI) consiste em uma linha teórica da psicometria que permite avaliar itens de um instrumento, validando-o ou não como capaz de mensurar determinada variável. Além disso, permite verificar a confiabilidade de instrumentos de mensuração.Verifica a habilidade dos indivíduos em responder determinados questionamentos (PASQUALI, 2009).

A classificação das respostas dadas a um item podem der dicotômicas, politômicas ou contínuas. Para esta pesquisa optou-se pela primeira opção onde cada item foi classificado como vulnerável ou não vulnerável.

A avaliação do item pode ser realizada pela TRI a um, dois ou três parâmetros, são elas: dificuldade (b) do item; dificuldade e discriminação(a); dificuldade, discriminação e a probabilidade de acerto ao acaso (c) (BORTOLOTTI *et al*., 2010; ANJOS, 2012; ARAÚJO *et al*., 2009; PASQUALI ,2009).

O acerto ao acaso consiste na probabilidade de uma pessoa mesmo com pouca habilidade/aptidão conseguir acertar um item. Ressalta-se também o traço latente que consiste na habilidade da pessoa avaliada responder corretamente um item. Existem dois princípios da TRI: unidimensionalidade e independência local. A primeira refere-se ao conjunto de itens que deve estar medindo um único traço latente (conhecimento). A segunda defende que a resposta dada em um item independe do outro item (PASQUALI; PRIMI, 2003).

Ressalta-se que quanto maior a habilidade/traço latente do indivíduo maior será a probabilidade de acertar o item (PASQUALI, 2009).

5 MÉTODO

5.1 TIPO DE ESTUDO

Trata-se de uma pesquisa de abordagem quantitativa do tipo transversal que ocorreu no período de fevereiro a maio de 2018.

Estudos transversais se caracterizam por demonstrar a realidade como se apresenta em um exato momento, apresentando uma fotografia dos fatos, desta forma, permite que não ocorra perda de amostra (HOCHMAN *et al.*,2005).

5.2 LOCAL DE COLETA DE DADOS

O projeto de pesquisa foi realizado na Casa da Acolhida para Pessoas em Situação de Rua, localizado na Rua Capitão José Pessoa, número 65, no bairro de Jaguaribe e no Centro de Referência Especializado para População em Situação de Rua (CENTRO POP), localizado na Rua Treze de Maio, 508 – Centro, João Pessoa, Paraíba, Brasil.

O CENTRO POP faz parte dos serviços ofertados pelo Sistema Único de Assistência Social (SUAS) que é realizado através de uma gestão compartilhada nos três níveis (municipal, estadual e federal). Encontra-se regulamentado pelo Conselho Nacional de Assistência Social (CNAS) através da aprovação do Serviço de Proteção Social Especial de Média Complexidade estabelecido na Resolução 109/2009 e pela Política Nacional para População em Situação de Rua através do Decreto 7053/2009 (BRASILb, 2009; BRASILa, 2009).

Destaca-se que o CENTRO POP é um espaço que funciona de segunda a sexta feira, nos turnos manhã e tarde, atende diariamente em média 50 pessoas em situação de rua que devem ter idade superior a 18 anos. Oferecem três refeições diárias, local para descanso e para realizar a higiene pessoal. Além disso, possuem equipe de suporte composta por educador, assistente social e psicólogo. Realizam atividades de estímulo a redução de danos, fazem encaminhamentos necessários para serviços de saúde de referência, promovem a melhoria das relações interpessoais, o respeito, autonomia e solidariedade. Neste local, as pessoas em situação de rua não podem adentrar se estiverem em uso de drogas, arma, material perfurocortante e bebida alcoólica, além de não permitir violência física e verbal. O acesso pode ocorrer por demanda espontânea, encaminhamento pela rede de serviços ou pelo Serviço Especializado de Abordagem Social.

A casa da acolhida abriga pessoas em situação de rua maiores de 18 anos por um período médio de 60 dias, quando tentam a ressocialização, auxiliam na inserção ao mercado de trabalho, retirada de documentos, encaminhamentos para outros serviços. Funciona por 24 horas, todos os dias da semana Neste local, as pessoas dormem, alimentam-se, fazem sua higiene pessoal, além de desenvolverem atividades lúdicas (jogos, rodas de capoeira e artes) por todo período de moradia.

Desta forma, destaca-se que os locais supracitados dispõem de ambiente limpo, calmo e individual, permitindo a ética, o compromisso, a responsabilidade em todos os momentos da coleta de dados, reduzindo a possibilidade de riscos aos participantes da pesquisa.

Salienta-se que estes locais de coleta de dados foram escolhidos, pois não seria possível realizar esta pesquisa com pessoas em situação de rua que não estivessem vinculados aos mesmos, uma vez que se realizou testes rápidos que exigem cuidados importantes para segurança dos participantes e dos pesquisadores, como: preservação da identidade dos participantes, acolhimento pré e pós teste, ambiente limpo, uso de materiais descartáveis e descarte em local adequado, iluminação adequada e encaminhamento para hospital de referência.

5.3 POPULAÇÃO E AMOSTRA

A população do estudo foi composta por 110 pessoas em situação de rua, a amostra foi constituída por 100 indivíduos, sendo 15 da Casa da Acolhida para Pessoas em Situação de Rua e 85 do CENTRO POP. Para calcular a amostra utilizou-se 95% de confiança e 3% de margem de erro, realizado com auxílio do Programa Statdisk 11.1.0.

A fórmula utilizada para amostragem foi (MOORE, 2000):

$$n = \frac{N.p.q.(Z)^2}{p.q(z)^2 + (N-1) . E^2}$$

*N= população (110)

n= amostra

p.q= proporção da população (0,25)

Confiança = 95%

E= Erro (3% = 0,03)

Z= Valor crítico (1,96)

$$n = \frac{110 . 0,25 . (1,96)^2}{0,25 . (1,96)^2 + (110-1) . (0,03)^2}$$

$$n = \frac{105,6}{1,0581}$$

n=99,801

5.4 CRITÉRIOS DE INCLUSÃO E EXCLUSÃO

Foram incluídos indivíduos em situação de rua com idade igual ou superior a 18 anos, com capacidade de comunicação verbal e que estivessem aptos a contribuir com a pesquisa no momento da coleta de dados.

Foram excluídas aquelas pessoas em situação de rua que apresentaram-se agressivos ou em uso de droga ou bebida alcoólica no momento da coleta de dados, bem como, aqueles que não assinassem o Termo de Consentimento Livre e Esclarecido (APÊNDICE A).

5.5 INSTRUMENTOS DE COLETA DE DADOS

Os dados foram coletados em forma de entrevista e exame clínicos, sendo o tempo médio para cada sujeito do estudo de duas horas. Foram utilizados nove instrumentos para coleta de dados descritos à seguir.

Justifica-se o uso de duas escalas de depressão, uma vez que a escala de Beck permite a classificação da depressão em quatro níveis (mínima, leve, moderada e grave), enquanto que a escala de Hamilton apesar de classificar a depressão em grave, moderada e leve, também pode classificar o indivíduo com presença ou ausência de depressão. Além disso, destaca-se que a escala de depressão de Beck avalia itens que não estão na escala de Hamilton e vice-versa.

5.5.1 Instrumento Sociodemográfico (APÊNDICE B)

As variáveis que compuseram o instrumento sociodemográfico incluiu as seguintes variáveis: idade, gênero, religião, filhos, motivo de estar na situação de rua, tempo que está em situação de rua, atividade remunerada desempenhada, número de filhos, estado civil ou presença de companheiro, procura periódica por serviço de saúde e acesso ao serviço de saúde.

Estas variáveis foram selecionadas com base em estudos que apresentam-nas como estatisticamente significativas à vulnerabilidade ao adoecer (VILLA et al., 2017; BALIEIRO *et al*., 2017; PAIVA *et al*., 2016).

5.5.2 Instrumento referente aos dados Clínicos e de vulnerabilidade ao HIV, sífilis e tuberculose. Testes rápidos para HIV, sífilis e Hepatite B e C (APÊNDICE C)

Os dados clínicos que foram coletados foram divididos em: gerais, específicos para vulnerabilidade ao adoecer (HIV/aids, sífilis, IST's), específicos para vulnerabilidade ao adoecer (tuberculose), testes rápidos (HIV, sífilis, hepatite B e C).

5.5.2.1 Dados Clínicos Gerais

Incluiu as seguintes variáveis: uso de medicamentos contínuos, sintomatologia (taquicardia, dor, tristeza, tosse, outros), sintomatologia relacionada a saúde mental (tontura, desmaios, esquecimento), etilismo, uso de drogas ilícitas (crack, maconha, ecstasy, outros), antecedentes familiares (diabetes, acidente vascular encefálico, hipertensão arterial, infarto agudo do miocárdio, outros), antecedentes pessoais (diabetes, acidente vascular encefálico, hipertensão arterial, infarto agudo do miocárdio, tuberculose, pneumonia, outros). (DEGENHARDT *et al*., 2017; FIORATI *et al*., 2015; SOUZA *et al*., 2016).

5.5.2.2 Vulnerabilidade ao adoecer: HIV/aids, sífilis e IST's

Para este aspecto admitiu-se as seguintes variáveis: idade da primeira relação sexual, diagnóstico e tratamento prévio de alguma Doença/Infecção Sexualmente Transmissível, utilização de preservativo com parceiro regular ou não (sexo anal, vaginal, oral), razão para não usar preservativo (prefere pele na pele, confiança, podem pensar que sou HIV positivo, preservativo não está disponível, outros); tipo de parceiro sexual (trabalhador do sexo, namorada, esposa e parceiro casual); atividade sexual com usuário de droga; relação sexual em troca de dinheiro; já apresentou algum sintoma de infecção sexualmente transmissível (corrimento, ardor e coceira); quais tipos de sexo realiza (oral, anal e vaginal); possui quantos parceiros sexuais; dor durante o ato sexual; sexo com parceiro com ferimento na região genital ou anal; parceiro com sintoma de infecção sexualmente transmissível;

compartilhamento de perfurocortantes; reutilização de agulhas; sofreu violência física e sexual (PINTO *et al*., 2014).

Realizou-se um escore para as variáveis referentes à vulnerabilidade ao HIV/aids, sífilis e outras IST's. O escore total são 16 pontos, sendo atribuído um ponto para comportamentos que apresentam vulnerabilidade e zero ponto para ausência de vulnerabilidade, conforme elenca o Quadro 1.

Quadro 1. Escore dos itens relacionados à vulnerabilidade ao HIV/aids, sífilis e outras IST's.

QUESTIONAMENTOS	ESCORE
Idade da 1ª relação sexual?	< 18 anos = 01<br>≥ 18 anos = 0
Diagnóstico prévio de alguma IST?	SIM = 1; NÃO = 0
Utiliza preservativo em todas as relações sexuais?	SIM = 0; NÃO = 1
Razão para não utilizar preservativo?	NÃO SE APLICA = 0; QUALQUER OUTRA = 01
Tipo de parceiro sexual?	Casual e/ou Trabalhador do sexo = 01; Namorado(a) e/ou esposo(a) = 0
Utiliza droga?	SIM = 1; NÃO = 0
Sexo com usuário de droga?	SIM = 1; NÃO = 0
Relação sexual em troca de dinheiro?	SIM = 1; NÃO = 0
Já apresentou algum sintoma de IST?	SIM = 1; NÃO = 0
Quantidade de parceiros?	1 = 0; >1 = 1
Dor durante o ato sexual?	SIM = 1; NÃO = 0
Sexo com parceiro com ferimento?	SIM = 1; NÃO = 0
Parceiro com sintoma de IST?	SIM = 1; NÃO = 0
Compartilhamento de perfurocortantes?	SIM = 1; NÃO = 0
Reutilização de agulhas?	SIM = 1; NÃO = 0
Já sofreu violência sexual?	SIM = 1; NÃO = 0

Fonte: Da pesquisa,2019.

5.5.2.3 Questionamentos/comportamentos quanto a vulnerabilidade à tuberculose

As variáveis deste aspecto incluíram quantitativo de banhos diários, aspecto/aparência suja/bem cuidada, escovação dos dentes, exposição à chuva, tosse, secreção, chiado no peito, febre periodicamente, tabagismo, ritmo respiratório (cheyne-stokes, biot, dispneico, kussmaul e eupneia), sons respiratórios (múrmuros vesiculares, creptos, roncos e sibilos) (ALECRIM *et al*., 2016; FIORATI *et al*., 2015; COSTA *et al*., 2015).

Estas variáveis foram elencadas e somadas as variáveis gerais (diagnóstico prévio de pneumonia e tuberculose) e criou-se um escore de vulnerabilidade à tuberculose, sendo atribuído um ponto para comportamentos vulneráveis e zero ponto para ausência de vulnerabilidade que encontram-se no Quadro 2. O escore máximo são 12 pontos.

Quadro 2. Escore referente à vulnerabilidade à tuberculose.

QUESTIONAMENTO	ESCORE
Quantidade de banhos diários.	≥1 = 0 Não toma banho = 1
Aspecto/aparência limpa.	SIM = 0; NÃO = 1
Escovação dos dentes.	≥1 vez ao dia = 0; < 1 vez ao dia = 1
Exposição à chuva.	SIM = 1; NÃO = 0
Tosse.	SIM = 1; NÃO = 0
Secreção amarelada ou esverdeada.	SIM = 1; NÃO = 0
Chiado no peito.	SIM = 1; NÃO = 0
Febre periodicamente.	SIM = 1; NÃO = 0
Tabagismo.	SIM = 1; NÃO = 0
Ritmo respiratório.	Cheyne-stokes= 1; Biot=1; Dispneico =1; Kussmaul=1; Eupneia= 0
Sons respiratórios	Múrmuros vesiculares = 0; Creptos=1; Roncos=1; Sibilos=1
Diagnóstico prévio de doenças respiratórias?	SIM= 1; NÃO = 0

Fonte: Da pesquisa,2019.

Para reverificar o ritmo respiratório utilizou-se a técnica de inspeção e palpação superficial que consiste em verificar com auxílio do órgão da visão e do tato a expansibilidade

torácica do indivíduo. Estas técnicas são realizadas com foco na região torácica do paciente (PORTO, 2012).

Para verificar os sons respiratórios utilizou-se a técnica de ausculta com auxílio do equipamento estetoscópio cardiológico da marca BIC. A técnica consistiu em colocar o diafragma do estetoscópio em pontos do hemitórax direito e esquerdo, nas regiões posterior, anterior e laterais do voluntário da pesquisa (PORTO, 2012). A ausculta foi realizada de forma simétrica, sempre um ponto do hemitórax direito e o mesmo no hemitórax esquerdo, solicitou-se ao voluntário da pesquisa inspirações profundas durante a ausculta. Estes pontos de ausculta estão representados conforme Figura 2.

Figura 2. Pontos de ausculta respiratória realizada nos voluntários da pesquisa.

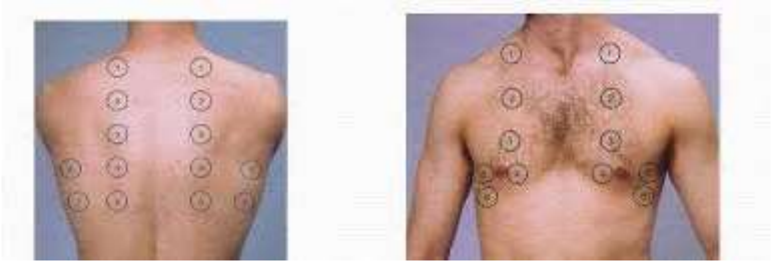

Fonte: Google imagens, 2019.

Na ausculta dos sons respiratórios, a presença de múrmuros vesiculares são os sons normais do ser humano durante a inspiração e expiração, os ruídos adventícios são sons patológicos, como os creptos que indicam presença de secreção viscosa e espessa e podem sugerir pneumonia; roncos predominam na expiração podem indicar presença de bronquite; sibilos surgem na inspiração e expiração indicam falta de ar, asma, obstrução, desconforto respiratório ou dispneia (PORTO,2012).

5.5.2.4 - Testes rápidos para HIV, sífilis, hepatite B e hepatite C

Todos os testes rápidos (HIV, sífilis, Hepatite B e Hepatite C) respeitaram o protocolo do Ministério da Saúde (2013), utilizando o teste imunocromatografia ou fluxo lateral. Para iniciar a coleta dos testes rápidos foi realizada a identificação da amostra com as iniciais do paciente, posteriormente a antissepsia na digital do paciente e a punção com uma lanceta descartável, pressionando levemente a extremidade maior da pipeta até fazer a sucção do sangue, em seguida foi dispensado duas gotas de sangue no orifício redondo do teste e uma gota do reagente.

Ressalta-se que o teste rápido para HIV foi da marca Bioclin, o teste rápido para sífilis da marca Alere, o teste rápido para hepatite B Biomérieux e o teste rápido para hepatite C da marca Alere.

O teste de HIV, sífilis e hepatites foram considerados positivos quando surgiram duas linhas, sendo uma no espaço controle (C) e outra no espaço teste (T), sendo considerado negativo quando surgiu apenas uma linha no “C”, como demonstra a Figura 3.

Figura 3. Leitura dos testes rápidos utilizados nesta pesquisa.

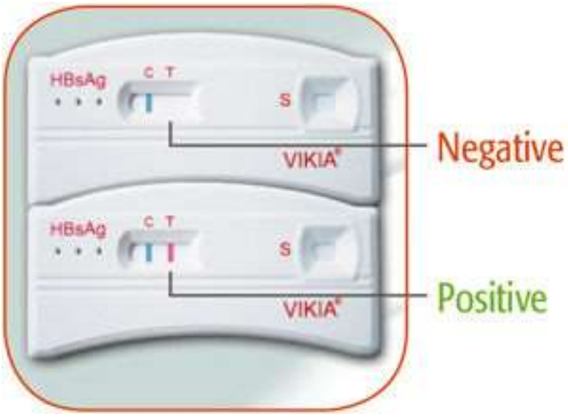

Fonte: Google imagens,2019.

As figuras 4,5,6,7 demonstram os passos realizados nos respectivos testes utilizados nesta pesquisa.

Figura 4. Passo a Passo do teste rápido para HIV realizado nos voluntários desta pesquisa.

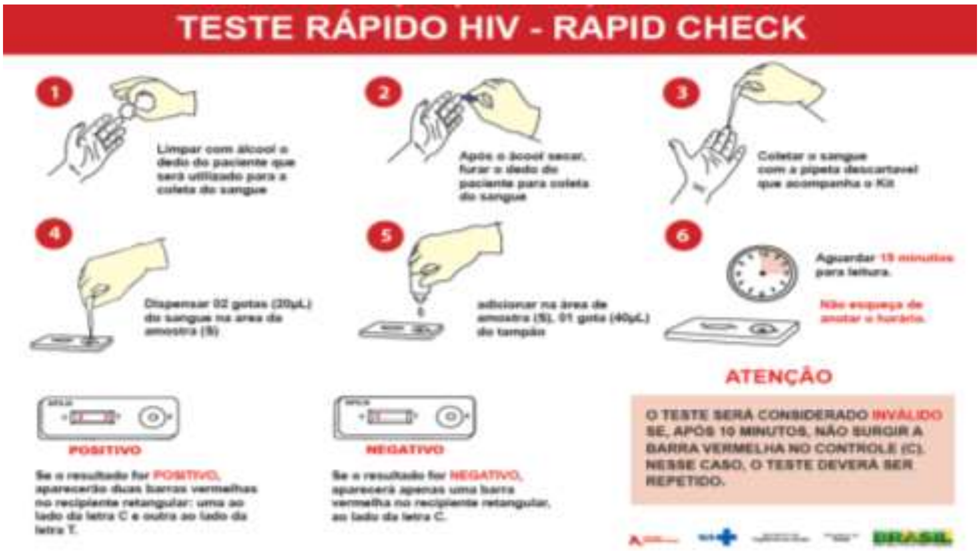

Fonte: Google imagens,2019.

Para o teste rápido de HIV a leitura foi realizada após 15 minutos. Salienta-se que o teste se torna inválido se após 10 minutos não aparecer nenhuma linha do controle, sendo dessa forma refeito (BRASIL,2013).

Quanto ao teste rápido para sífilis a leitura foi realizada após 20 minutos, os passos seguidos para realização do teste encontram-se na Figura 5 (BENZAKEN *et al.*, 2013; ALERE,2017).

Figura 5. Passo a Passo do teste rápido para sífilis realizado nos voluntários da pesquisa.

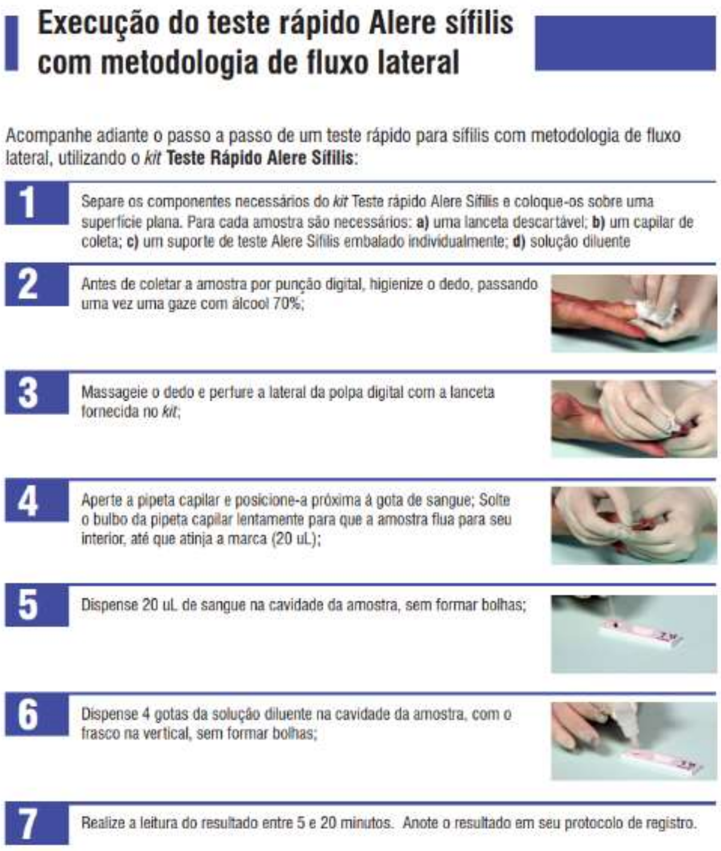

Fonte: Alere, 2017.

O teste para Hepatite B seguiu as etapas descritas por Biomérieux (2016) e foi interpretado em 30 minutos.

Figura 6: Etapas para realização do teste de Hepatite B.

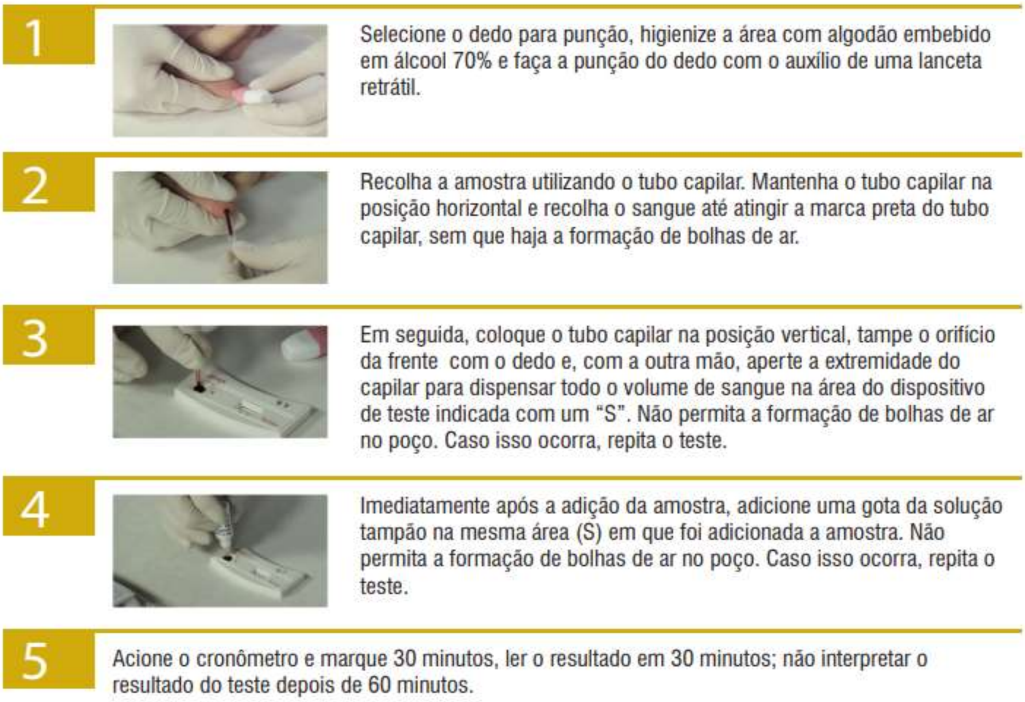

Fonte: Biomérieux, 2016.

Os passos que foram seguidos para realização do teste para Hepatite C estão descritos na Figura 7 e o resultado foi obtido após 20 minutos (ALERE, 2016).

Figura 7. Passo a Passo do teste rápido de Hepatite C realizado nos voluntários da pesquisa.

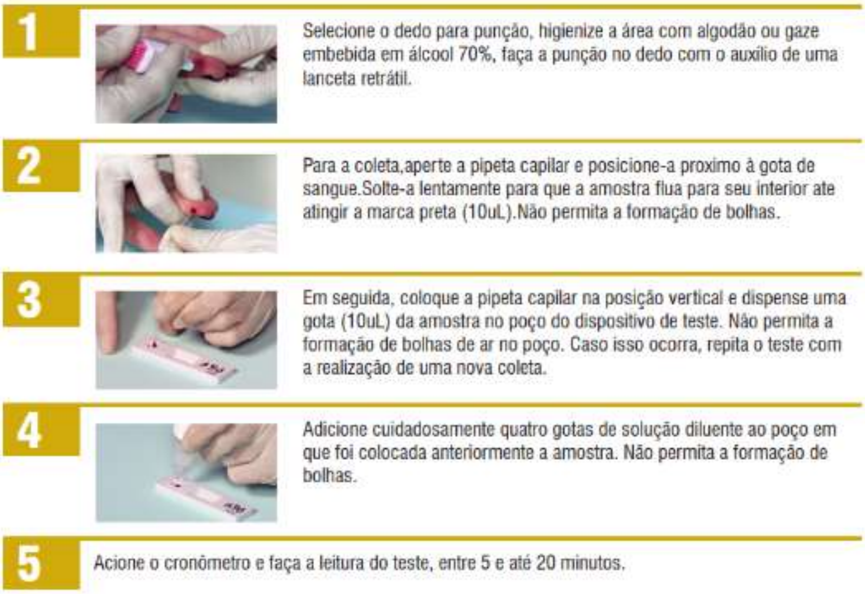

Fonte: Alere, 2016.

5.5.3 Instrumento: Escala Self-Reporting Questionnaire (SRQ 20) (ANEXO A)

Dentre os instrumentos precisos, validados nos Brasil que possibilitam identificar transtornos mentais comuns em adultos está a escala SRQ20. A consistência interna desta escala foi avaliada em uma população de trabalhadores brasileiros com proporção de variância de 59,6%, sendo considerado viável para identificar fatores que podem rastrear o transtorno mental comum. A sensibilidade e especificidade deste instrumento obteve 0,696 a 0,882 (SANTOS *et al*., 2009).

Esta escala identifica transtornos mentais comuns (insônia, fadiga, irritabilidade, esquecimento, dificuldade de concentração e queixas somáticas), é composta de 20 questões, sendo quatro referentes a sintomas físicos e 16 psicoemocionais, com alternativas de respostas sim ou não. O indivíduo que obteve escore com sete ou mais respostas "sim" foi considerado com transtorno mental comum (SANTOS *et al*., 2009; GONÇALVES *et al*., 2008).

5.5.4 Instrumento: Escala de Ansiedade de Beck - BAI (ANEXO B)

A escala de ansiedade de Beck é validada no Brasil, apresenta alfa de Cronbach de 0,76. Avalia a ansiedade através de 21 questionamentos. Cada item possui quatro opções de respostas: absolutamente não (0), levemente (1), moderadamente (2) e gravemente (3), cujo escore máximo atinge 63 pontos. A classificação está dividida em: nível mínimo de ansiedade (0 a 10 pontos); nível leve de ansiedade (11 a 19 pontos); nível moderado de ansiedade (20 a 30 pontos) *e* nível grave de ansiedade (31 a 63 pontos) (CUNHA, 2001).

5.5.5 Instrumento: Escala de Depressão de Beck –BDI (ANEXO C)

A escala de depressão de Beck foi desenvolvida por Beck *et al.*, (1961) validada no Brasil com alfa de Conbach 0,81 (GORENSTEIN; ANDRADE, 1996). No Brasil, esta escala foi aplicada em estudantes universitários da cidade de São Paulo, sendo realizada validação fatorial por Gorenstein e Andrade (1998). Também foi realizada validação fatorial por Cunha (2001) em uma amostra de dependentes de álcool.

Esta escala apresenta 21 descrições de sintomas de depressão, com alternativas de zero a três pontos. Esta escala classifica a depressão em níveis: mínimo (0 a 11 pontos), leve (12 a 19 pontos), moderado (20 a 35 pontos) e grave (36 a 63 pontos) (CUNHA, 2001).

5.5.6 Instrumento: Escala de Desesperança de Beck - BHS (ANEXO D)

A escala de desesperança de Beck foi desenvolvida originalmente no Center of Cognitive Therapy (CCT) da Universidade da Pensilvânia, na Filadélfia, nos Estados Unidos. A escala é validada no Brasil, composta por 20 itens com opções de respostas certo ou errado, permitindo verificar o pensamento pessimista e negativo do voluntário da pesquisa em relação ao futuro. Ressalta-se que a respectiva escala permite também avaliar a possibilidade de suicídio (CUNHA, 2001).

Nesta escala, cada item, pode pontuar zero ou um, sendo quanto mais alto o escore, maior o nível de desesperança. Permite classificar o indivíduo da seguinte forma: mínimo (zero a quatro); leve (5 a 8); moderado (9 a 13) e grave (14 a 20) (CUNHA, 2001). Estabelece-se o escore ≥9 como maior suscetibilidade ao suicídio.

5.5.7 Instrumento: Escala de Resiliência (ANEXO E)

A escala de Resiliência de Wagnild e Young (1993) adptada por Perce (2005) avalia questões psicossociais positivas em decorrência de eventos importantes com índice de consistência alfa de Cronbach de 0,80 (WAGNILD, 2009). Foi validada no Brasil com estudantes de escolas públicas.

Apresenta 25 itens que engloba cinco temas: serenidade, perseverança, autoconfiança, sentido de vida e autosuficiência. As respostas variam de 1 (discordo totalmente) a 7 (concordo totalmente), com escore mínimo de 25 e máximo 175 pontos, sendo quanto mais elevado o escore, maior a resiliência. Escore até 125 indica baixa resiliência, 125 a 145 média e acima de 145 alta (PESCE *et al.*, 2005).

5.5.8 Instrumento: Escala de Depressão de Hamilton (ANEXO F)

A escala de depressão de Hamilton avalia itens específicos, como: somáticos, cognitivos, motores, ansiedade, humor e sociais. É considerada padrão ouro para verificar a depressão, validada no Brasil em jovens, apresenta alfa de Cronbach >0,70 (BAGBY *et al.*, 2004; FREIRE *et al.*, 2014).

Composta por 17 itens com mínimo de zero e total de 52 pontos, sendo considerado escore ≥25 como depressão grave, entre 18 e 24 moderadamente deprimido, entre 7 e 17 pontos depressão leve. Para que o indivíduo seja classificado com algum grau de depressão

precisa pontuar um escore de no mínimo oito (GUILLEMIN, 1993; MORENO 1998; GALLUCCI NETO; CAMPOS JÚNIOR; HUBNER, 2001).

5.5.9 Instrumento: Questionário de Vulnerabilidade ao HIV/aids (ANEXO G)

O Questionário referente a vulnerabilidade ao HIV/aids apresenta 11 questões com respostas divididas em cores verde, amarela e azul, sendo a pontuação 0,1 e 2, conforme as cores respectivamente. A soma das respostas variam entre 0 e 22 pontos, escore entre zero e quatro indica baixa vulnerabilidade, cinco e onze moderada e alta entre 12 e 22 (BRASIL, 2006; COSTA, 2017). O questionário apresenta as seguintes temáticas: início das relações sexuais, uso do preservativo e gravidez, exposição ao HIV, serviços de saúde, teste de HIV, diálogo, facilidades e dificuldades para aquisição do preservativo.

5.6 PROCEDIMENTOS DE COLETA DOS DADOS

A coleta de dados seguiu etapas, de modo a facilitar a operacionalização e padronizar os procedimentos realizados. A Figura 8 demonstra os passos seguidos.

Figura 8. Etapas da coleta de dados desta pesquisa.

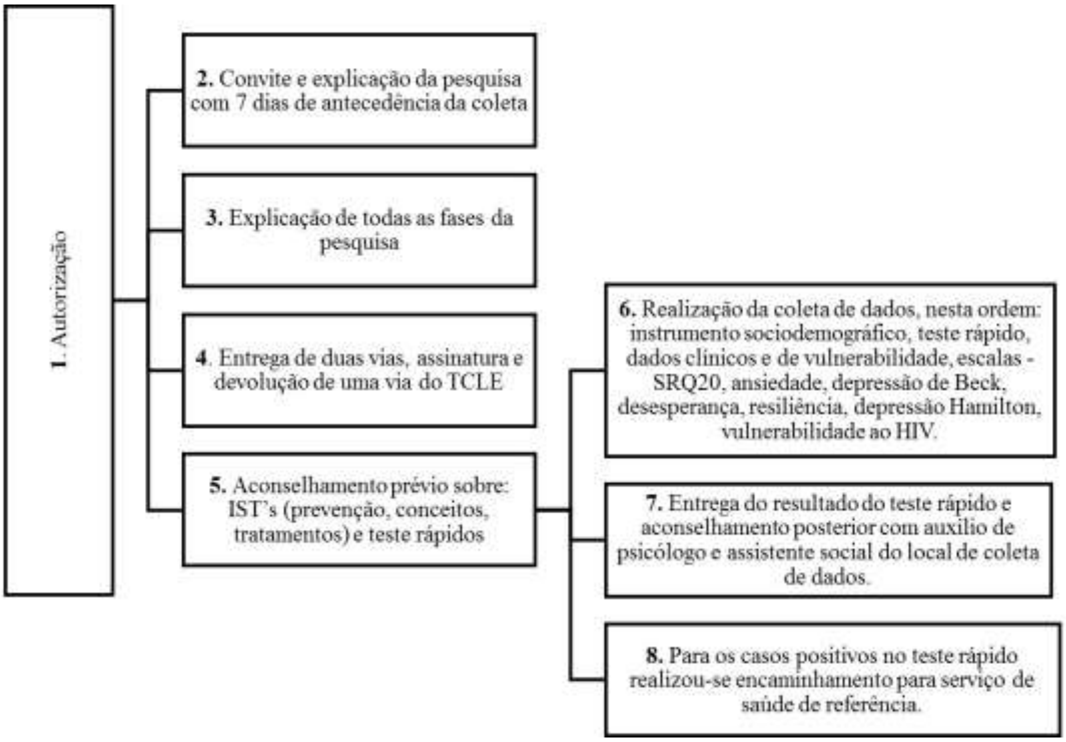

Fonte: Da pesquisa,2019. TCLE = Termo de Consentimento Livre e Esclarecido

A etapa 1 compreendeu a solicitação de autorização para realização da pesquisa. Este procedimento foi requisitado ao responsável pelo local de coleta de dados na Secretaria de Assistência Social da Prefeitura Municipal de João Pessoa, posteriormente comunicação ao funcionário do respectivo local. Também realizou-se a submissão ao comitê de ética em pesquisa.

A etapa 2 ocorreu após autorização pelo comitê de ética. A pesquisadora apresentou-se aos locais de coleta de dados, explicou todas as etapas da mesma e com auxílio de funcionários do local de coleta de dados, convidou algumas pessoas em situação de rua para participarem da pesquisa, agendando a coleta de dados com um período de sete dias de antecedência.

Na etapa 3, na data agendada para coleta de dados, foi realizada de forma individual, a explicação de todas as fases de coleta de dados e os objetivos da pesquisa. Neste momento, ocorreu a etapa 4 com a entrega de duas vias do TCLE, retornando uma via assinada ou com impressão digital para a pesquisadora.

Após este momento ocorreu a etapa 5 que consistiu em um acolhimento prévio, de forma individual, em que era explicado conceitos e esclarecido dúvidas sobre IST's, bem como quanto aos testes rápidos.

Na etapa 6 houve, de fato, a coleta de dados, quando optou-se primeiramente pela realização do questionário sociodemográfico, pois torna-se necessário identificar o teste rápido e os instrumentos com as iniciais do voluntário da pesquisa. Posteriormente aplicou-se o teste rápido visto que seu resultado não é obtido de forma imediata, desta forma, otimizava-se o tempo. Os demais instrumentos foram coletados logo à seguir.

Após aplicação de todos os instrumentos, o voluntário da pesquisa era convidado a adentrar em uma sala de forma individual para receber o resultado do exame (etapa 7). Após explicação do resultado, era realizado um aconselhamento posterior, independentemente de ser positivo ou não, contando com a presença de assistente social e psicólogo.

Na etapa 8 para aqueles com resultados positivos, foram encaminhados para o serviço de saúde de referência para doenças infectocontagiosas no município de João Pessoa/PB.

5.6.1 Equipe de coleta de dados

Destaca-se que a equipe de coleta de dados foi composta por uma doutoranda (enfermeira e professora) e seis graduandos em enfermagem treinados previamente através de

um mini curso com duração de seis horas, quando houve descrição dos instrumentos utilizados na pesquisa e simulação de aplicação dos mesmos, padronizando a linguagem e a forma de coleta de dados.

5.6.2 Equipamentos utilizados na coleta de dados

Ressalta-se que os equipamentos eram utilizados de forma individual, descartados a cada atendimento (testes rápidos, equipamentos de proteção individual e algodão), desprezados em local correto e que antes de todo atendimento realizou-se higienização simples das mãos e desinfecção simples com fricção de álcool no estetoscópio. Os equipamentos utilizados foram:

- Estetoscópio cardiológico da marca BIC;
- Teste rápido para HIV da marca Bioclin;
- Teste rápido para sífilis da marca Alere;
- Teste rápido para hepatite B Biomérieux;
- Teste rápido para hepatite C da marca Alere;
- Caixa de material perfurocortante;
- Equipamentos de Proteção Individual: gorro, luvas de procedimento, máscara;
- Sabonete líquido neutro;
- Álcool 70%;
- Algodão.

5.7 ÉTICA DA PESQUISA

A pesquisa seguiu os princípios éticos estabelecidos na Resolução n°466/2012 do Conselho Nacional de Saúde que diz respeito às Diretrizes e Normas Regulamentadoras de Pesquisa Envolvendo Seres Humanos (BRASILb, 2012).

Esta pesquisa foi aprovada pelo Comitê de Ética em Pesquisa, conforme Certificado de Apresentação para Apreciação Ética (CAAE) 79486517000005176 (ANEXO H), parecer 2456847. Destaca-se que foi assinalado o Termo de Compromisso de utilização e divulgação dos dados (APÊNDICE D). Além disto, cada participante recebeu duas vias do Termo de Consentimento Livre e Esclarecido. Ressalta-se ainda a carta de anuência fornecida pela

Secretaria de Desenvolvimento Pessoal, órgão responsável pelos locais de coleta de dados (ANEXO I).

5.8 PROCEDIMENTOS DE ANÁLISE DOS DADOS

Os dados foram processados pelo programa Statistical Package for the Social Sciences (SPSS) - versão 19.0 e pelo programa R pacote 3.3.1 gratuito, de domínio público. Além disso, utilizou-se o software MPLUS. A estatística seguiu etapas, sendo iniciada de forma descritiva com frequência absoluta e relativa, média, desvio padrão da média, máximo e mínimo das variáveis.

Segunda etapa: validação do questionário composto por 16 itens referentes a vulnerabilidade ao HIV e outras IST's por meio da Teoria de Resposta ao Item.

Quadro 3. Itens do questionário validado sobre à vulnerabilidade ao HIV/aids, sífilis e outras IST's.

QUESTIONAMENTOS	ESCORE
Idade da 1ª relação sexual?	< 18 anos = 0<br>≥ 18 anos = 01
Diagnóstico prévio de alguma IST?	SIM = 0; NÃO =01
Utiliza preservativo em todas as relações sexuais?	SIM = 01; NÃO = 0
Razão para não utilizar preservativo?	NÃO SE APLICA = 01; QUALQUER OUTRA = 0
Tipo de parceiro sexual?	Casual e/ou Trabalhador do sexo = 01; Namorado(a) e/ou esposo(a) = 0
Utiliza droga?	SIM = 0; NÃO = 01
Sexo com usuário de droga?	SIM = 0; NÃO = 01
Relação sexual em troca de dinheiro?	SIM = 0; NÃO = 01
Já apresentou algum sintoma de IST?	SIM = 0; NÃO = 01
Quantidade de parceiros?	1 = 01; >1 = 0
Dor durante o ato sexual?	SIM = 0; NÃO = 01
Sexo com parceiro com ferimento?	SIM = 0; NÃO = 01
Parceiro com sintoma de IST?	SIM = 0; NÃO = 01
Compartilhamento de perfurocortantes?	SIM = 0; NÃO = 01
Reutilização de agulhas?	SIM = 0; NÃO = 01
Já sofreu violência sexual?	SIM = 0; NÃO = 01

Fonte: Da pesquisa,2019.

Terceira etapa: validação do questionário composto por 12 itens referentes a vulnerabilidade à tuberculose por meio da Teoria de Resposta ao Item.

Quadro 4. Itens do questionário validado referente à vulnerabilidade à tuberculose.

**QUESTIONAMENTO**	**ESCORE**
Quantidade de banhos diários.	≥1 = 01 Não toma banho = 0
Aspecto/aparência limpa.	SIM = 01; NÃO = 0
Escovação dos dentes.	≥1 vez ao dia = 01; < 1 vez ao dia = 0
Exposição à chuva.	SIM = 0; NÃO = 01
Tosse.	SIM = 0; NÃO = 01
Secreção amarelada ou esverdeada.	SIM = 0; NÃO = 01
Chiado no peito.	SIM = 0; NÃO = 01
Febre periodicamente.	SIM = 0; NÃO = 01
Tabagismo.	SIM = 0; NÃO = 01
Ritmo respiratório.	Cheyne-stokes= 0; Biot=0; Dispneico =0; Kussmaul=0; Eupneia= 01
Sons respiratórios	Múrmuros vesiculares = 01; Creptos=0; Roncos=0; Sibilos=0
Diagnóstico prévio de doenças respiratórias?	SIM= 0; NÃO = 01

Fonte: Da pesquisa,2019.

Para a segunda e terceira etapa atribuiu-se um ponto para os comportamentos não vulneráveis e zero para vulnerabilidade. Utilizou-se a Teoria de Resposta ao Item (TRI) a dois parâmetros, sendo: discriminação(a) e dificuldade(b). O parâmetro acerto ao acaso (c) foi descartado, pois neste tipo de questionário não existe uma resposta certa ou errada, desta forma não sendo influenciada por “chutes” para encontrar uma alternativa correta, uma vez que o indivíduo respondeu com base nos seus comportamentos individuais. Os valores de discriminação não foram aceitos quando negativos e esperou-se o grau de dificuldade não muito elevado.

Para dificuldade(b) foi considerado aceitável entre -2 e + 2; para discriminação(a) foram considerados os valores entre 0 e +2. A carga fatorial foi considerada adequada quando obteve valor no mínimo de 0,30 para que o item permanecesse no instrumento.

O modelo TRI a dois parâmetros é dado por:

$$P(U_{ij} = 1|\theta_j) = \frac{1}{1 + e^{-Da_i(\theta_j - b_i)}},$$

com $i = 1, 2, \cdots, I$, e $j = 1, 2, \cdots, n$.

O valor de D representado na equação é uma constante igual a 1,7 que aproxima a curva logística da distribuição normal.

A relação entre respostas que indicam ausência de vulnerabilidade (eixo vertical) e a respectiva habilidade (eixo horizontal) do indivíduo é descrita através da Curva Característica do Item (CCI) que é representada por uma curva sigmoide (formato de S).

Além disto, ressalta-se a análise fatorial com valor p que deve ser >0,05; Raíz Quadrada Média dos Quadrados dos Erros de Aproximação (RMSEA) no máximo 0,08; Índice Quadrático Médio (RMSR) com valor desejável de 0,07; Índice de Tucker-Lewis (TLI) e Índice de Ajuste Comparativo (CFI) com valor mínimo de 0,90; Utilizou-se também a carga fatorial do item sendo aceitável quando >0,30 (HAIR, 2005).

Acrescenta-se ainda a realização de teste diagnóstico através da Curva Receive Operation Characteristics (ROC) e do ponto de corte, com o objetivo de encontrar o escore do instrumento sobre vulnerabilidade ao HIV e a tuberculose, que representa como de risco, bem como sua respectiva comparação com o teste padrão ouro (para HIV teste rápido e para tuberculose foi o autorrelato). Quanto mais a curva ROC se aproximar do número um, melhor será o teste.

Em geral se escolhe na curva ROC o ponto superior mais à esquerda e observa-se que quanto maior for a área sob esta curva mais exato será o teste. Considerando que a incidência desta doença é muito baixa, na amostra estudada, nesta pesquisa, se dará mais atenção à sensibilidade. Neste estudo, para o ponto de corte, foram elencados os seguintes parâmetros: sensibilidade, especificidade, acurácia, valor preditivo positivo e negativo. A sensibilidade consiste na probabilidade de um teste ser positivo quando há a doença. A especificidade é a capacidade do teste dar negativo quando não se tem o diagnóstico. A acurácia refere-se a como um teste discrimina corretamente a saúde e a doença. O valor preditivo positivo refere-se ao indivíduo doente realmente ter a doença. O valor preditivo negativo é a probabilidade da pessoa com teste negativo não estar doente. Uma sensibilidade alta gerará um valor preditivo negativo alto, uma especificidade alta gerará um valor preditivo positivo alto. Pontos de corte altos referem-se a pouca sensibilidade e muita especificidade, e os baixos são muito sensíveis e pouco específicos. (METZ, 1986; EUSEBI, 2013).

Quarta etapa: para verificar a influência de variáveis sobre o diagnóstico de HIV confirmado por meio do teste rápido e do diagnóstico prévio de tuberculose (autorrelatado) foi realizado o Weight of Evidence (WoE) – Peso da Evidência.

As variáveis que foram testadas pelo WoE para verificar a influência sobre o HIV e a tuberculose, foram divididas em três classes/categorias/fatores, sendo:

- Psíquicos: desesperança, ansiedade, depressão, resiliência, transtornos mentais.
- Sociodemográficos: tempo de moradia na rua, idade, presença de companheiro/estado civil, gênero.
- Comportamentais: prática de sexo por via oral, anal e vaginal.

Foi realizado o WoE através da medida de Information Value (IV) por meio de gráficos com auxílio do software R, gratuito (PRABHAKARAN,2016). O WoE consiste em um parâmetro que permite verificar a influência de variáveis revelando a sua intensidade, podendo ser usado para interpretar evidências referentes à riscos relacionados à saúde. Para calcular o WoE utiliza-se um logaritmo da razão de chances ou Odds Ratio (OR): WoE=In (OR).

O IV permite mensurar a força de cada variável sobre o desfecho, podendo ser classificado em Very Strong = influência muito forte (≥0.5) ; Strong= influência forte (≥0.3); Average = influência média ($0.1 \leq IV \leq 0.3$); Weak= influência fraca ($0.02 \leq IV \leq 0.1$); Very Weak= influência muito fraca ($IV < 0.02$) (SIDDIQI, 2006). O cálculo do IV pode ser encontrado pela equação:

$$IV = \sum_{i\,=\,1}^{n} (a_i/c_i - b_i/d_i) \times WOE$$

Quinta etapa: construção de um dendograma através do software R, gratuito, para verificar/comprovar a divisão dos três grandes fatores (psíquicos, sociodemográficos e comportamentais) que influenciam à vulnerabilidade ao HIV e a Tuberculose em pessoas em situação de rua.

Utilizou-se o agrupamento hierárquico com o método da ligação média da distância em que os valores das variáveis foram substituídos pelos seus respectivos escores padronizados e distância euclidiana, pois as variáveis são mistas (dicotômicas e contínuas), sendo necessário padronizá-las para então realizar o agrupamento hierárquico.

O dendograma permite representar graficamente e organizar fatores através da similaridade, sendo quanto maior a proximidade entre as variáveis, maior a similaridade, possibilitando uma visão bidimensional do conjunto de fatores.

Sexta etapa: Modelo de classe latente (Latent Class model = LCA), realizado no software R, gratuito, de domínio público através do pacote poLCA.

O LCA permite verificar o quantitativo de classes/grupos da amostra no aspecto de intersecção de características individuais. Para a escolha dos grupos, levou-se em consideração os seguintes parâmetros: parâmetros estimados, graus de liberdade dos resíduos, Lg Verossimilhança, Critério de Informação Akaike (AIC), Critério de Informação Bayesiano (BIC) que quanto menor melhor, Razão de verossimilhança (G^2), X^2 (qui-quadrado com

p≥0,05), entropia relativa (%) que quanto maior melhor (HAGENAARS; MCCUTCHEON, 2002).

O critério AIC é calculado pela fórmula:

$$AIC = 2k - 2ln(L)$$

O critério BIC é calculado pela fórmula:

$$BIC = k\ ln(n) - 2ln(L)$$

Nestas fórmulas K é o número de parâmetros, L o valor maximizado da função da verossimilhança, n é o tamanho da amostra.

Para AIC e BIC quanto menor seu valor mais adequado é o modelo. A entropia relativa permite verificar se a classificação das classes são satisfatórias para a amostra, sendo adotado valores entre 0 e 100%, quanto mais próximo a 100 melhor a definição das classes, demonstrando quantas vezes os indivíduos foram classificados corretamente nas classes latentes.

A entropia relativa é dada pela fórmula:

$$ENTROPY = 1 - \frac{S}{(n \ln(C))}$$

S é a entropia bruta, n o tamanho da amostra e C o número de classes latentes.

Posteriormente foi realizada a interpretação do LCA, no intuito de identificar as características dos indivíduos que estão presentes em cada classe, sendo caracterizados como alto, médio e razoável risco.

Sétima etapa: foi realizada a análise fatorial confirmatória para um modelo no intuito de verificar a associação das variáveis com os fatores psíquicos, sociodemográficos e comportamentais.

Os parâmetros utilizados foram: Gl (graus de liberdade), valor p≥0,05, CFI e TLI >0,90, RMSEA no máximo 0,08, SMR no máximo 0,10. Este procedimento foi realizado no Software R, gratuito, de domínio público.

Além disso, utilizou-se o Software MPLUS para construir uma figura representativa da análise fatorial confirmatória.

5.9 RISCOS

Salienta-se que segundo a Resolução 466/12 toda pesquisa que envolve seres humanos oferece riscos. Não obstante os riscos potenciais, as pesquisas envolvendo seres humanos serão admissíveis quando oferecerem elevada possibilidade de gerar conhecimento para entender, prevenir ou aliviar um problema que afete o bem-estar dos sujeitos da pesquisa e dos outros indivíduos.

Nesse sentido, o referido estudo pode oferecer desconforto de origem psicológica/ emocional uma vez que o sujeito da pesquisa foi submetido a questionários contendo informações sociodemográficas, dados clínicos e práticas/comportamentos vulneráveis ao HIV/aids, sífilis e tuberculose, ausculta respiratória através de técnicas de exame físico, testes rápidos para HIV, sífilis, Hepatite B e Hepatite C. Para minimizar esse risco, a coleta de dados foi realizada individualmente e em local reservado.

Ressalta-se que durante o exame físico (ausculta respiratória) e testes rápidos pode ocorrer desconforto e incômodo com o posicionamento ou o próprio exame. Para minimizar esse risco, a coleta de dados foi realizada individualmente e em um local reservado adequado para avaliação clínica: ambiente arejado, iluminado e com presença de cadeira, deixando o ambiente limpo e confortável. Além disso, há o risco biológico pois envolve sangue ou fluídos (testes rápidos), mas para minimizar estes riscos todos os exames foram realizados com o uso de Equipamentos de Proteção Individual e foi realizada a higienização das mãos ao atender cada participante da pesquisa. As lancetas e kits de avaliação eram descartáveis e utilizados de forma individual, descartados em local adequado, não havendo risco de contaminação.

Quanto aos testes rápidos os sujeitos da pesquisa receberam informações/orientações/aconselhamentos antes do exame e após o resultado. Este resultado foi revelado de forma sigilosa e individual em ambiente fechado dentro do próprio local de coleta de dados. Para os resultados positivos além de orientações fornecidas pelos pesquisadores, o sujeito da pesquisa foi aconselhado a procurar o Serviço de Saúde de Referência para Doenças Infectocontagiosas no município de João Pessoa/PB. Além disso, ressalta-se a presença de psicólogo e assistente social do local de coleta de dados de forma a auxiliar no encaminhamento dos casos positivos.

Foi garantido que caso o sujeito da pesquisa sofresse algum dano comprovadamente em decorrência deste estudo assumiu-se o compromisso de interromper imediatamente a pesquisa, e se necessário, foi garantido ao participante do estudo acompanhamento e

assistência imediata e/ou integral fornecida pela equipe de saúde da instituição que os coordenadores estão vinculados. Além disso, salienta-se o direito do participante, quando preciso, ter acompanhamento posterior e plena liberdade de recusar-se a participar ou retirar o consentimento em qualquer fase da pesquisa, sem penalização alguma, sendo garantida a manutenção do sigilo e da sua privacidade durante todas as fases da mesma.

Informa-se ainda que o risco se justifica pela importância do benefício esperado, haja vista que pesquisas como estas podem oferecer grandes contribuições à sociedade e a comunidade científica de um modo geral na medida em que seus resultados contribuem para a construção de estratégias que possibilitam uma melhor assistência as pessoas em situação de rua.

6 RESULTADOS

Os resultados encontram-se divididos em três seções: características sociodemográficas e clínicas; saúde mental/fatores psíquicos e testes rápidos, comportamentos e fatores que predispõem a vulnerabilidade ao HIV, IST's e tuberculose.

6.1 Características sociodemográficas e clínicas

Das 100 pessoas em situação de rua que participaram da pesquisa, 84% eram homens, tinham idade entre 18 e 62 anos, vivem nas ruas a um tempo mínimo de dois meses e máximo de 55 anos, quanto a presença de companheiro/estado civil, filhos, religião, motivo de moradia na rua, procura e acesso ao serviço de saúde a Tabela 1 demonstra os resultados.

Tabela 1. Caracterização de pessoas em situação de rua. João Pessoa, Paraíba, Brasil, 2018. N=100.

Variáveis	N	%
**Presença de companheiro/estado civil**		
Solteiro	67	67%
Casado/companheiro(a)	22	22%
Divorciado(a)	11	11%
**Filhos**		
Sim	58	58%
Não	42	42%
**Religião**		
Católica	49	49%
Não tem	29	29%
Evangélica	20	20%
Espírita	1	1%
Candomblé	1	1%
**Motivo de moradia na rua**		
Confronto familiar	45	45%
Uso de drogas	35	35%
Desejo de Liberdade	6	6%
Não possui dinheiro	7	7%
Depressão	1	1%
Desemprego	1	1%
Homicídio	2	2%
Não tem casa	1	1%
Tráfico	1	1%
Nasceu na rua	1	1%
**Procura periódica por serviço de saúde**		
Não procura	36	36%
Anualmente	29	29%
A cada 3 meses	18	18%
A cada 6 meses	7	7%
De 2 em 2 anos	5	5%
Só procura quando tem sintoma	4	4%
A cada 15 dias	1	1%
**Acesso ao serviço de saúde**		

Bom	41	41%
Não tenho acesso	23	23%
Regular	16	16%
Ótimo	11	11%
Não preciso	5	5%
Ruim	4	4%

Fonte: Da pesquisa,2019.

Quanto as doenças prévias, manifestações clínicas, uso de medicamentos e drogas ilícitas a Tabela 2 descreve estas informações.

Tabela 2. Distribuição das características relacionadas aos dados clínicos gerais de pessoas em situação de rua. João Pessoa, Paraíba, Brasil, 2018. N=100.

Variáveis	n	%
**Doenças prévias (familiares)**		
Hipertensão arterial	38	38%
Diabetes mellitus	35	35%
Acidente Vascular Encefálico	9	9%
Infarto Agudo do Miocárdio	9	9%
**Doenças prévias (pessoais)**		
Tuberculose	15	15%
Pneumonia	14	14%
Diabetes mellitus	7	7%
Hipertensão arterial	5	5%
Infarto Agudo do Miocárdio	1	1%
Acidente Vascular Encefálico	-	-
**Sintomatologia/Manifestações clínicas**		
Dor	37	37%
Tristeza	34	34%
Tosse	20	20%
Taquicardia	15	15%
**Sintomatologia relacionada a saúde mental**		
Esquecimento	44	44%
Cefaleia	38	38%
Tontura	37	37%
Visão turva	25	25%
Desmaios	9	9%
**Uso de medicamento contínuo**		
Não	73	73%
Sim	27	27%
**Etilismo**		
Sim	68	68%
Não	32	32%
**Drogas ilícitas**		
Sim	74	74%
Não	26	26%
**Quais drogas?**		
Maconha	57	57%
Crack	54	54%
Cocaína	17	17%
Artane	5	5%
Outros	4	4%

Fonte: Da pesquisa,2019.

6.2 Saúde mental/fatores psíquicos

No que diz respeito a resiliência, transtornos mentais comuns, ansiedade e depressão, a Tabela 3 aborda que a maioria das PSR desta pesquisa, apresentam transtornos mentais comuns, ansiedade e desesperança mínima, baixa resiliência e depressão leve.

Tabela 3. Resiliência, transtornos mentais comuns, ansiedade, desesperança e depressão de pessoas em situação de rua. João Pessoa, Paraíba, Brasil, 2018. N=100.

Variáveis	n	%
**Transtornos mentais comuns (SRQ20)**		
Presença	79	79%
Ausência	21	21%
**Ansiedade**		
Mínimo	37	37%
Leve	37	37%
Moderado	22	22%
Grave	4	4%
**Desesperança**		
Mínimo	29	29%
Leve	29	29%
Moderado	26	26%
Grave	16	16%
**Resiliência**		
Baixa	48	48%
Média	32	32%
Alta	20	20%
**Depressão Escala de Beck**		
Leve	44	44%
Mínimo	30	30%
Moderado	26	26%
Grave	-	-
**Depressão Escala de Hamilton**		
Presente	69	69%
Ausente	31	31%

Fonte: Da pesquisa,2019.

6.3 Testes rápidos, comportamentos e fatores que predispõem a vulnerabilidade ao HIV, IST's e tuberculose

Tratando-se de aspectos preditores de infecções sexualmente transmissíveis, como resultados de testes rápidos para HIV, sífilis, hepatite B e hepatite B, além dos comportamentos vulneráveis a infecção ao HIV, sífilis e outras IST's em pessoas em situação de rua, a Tabela 4 revela informações importantes que podem sugerir maior vulnerabilidade destas pessoas ao adoecimento.

Tabela 4. Testes rápidos e comportamentos vulneráveis ao adoecer relacionados à infecção ao HIV, sífilis e outras IST's de pessoas em situação de rua. João Pessoa, Paraíba, Brasil, 2018. N=100.

Variáveis	n	%
**TESTES RÁPIDOS**		
**Sífilis**		
Negativo	70	70%
Positivo	29	29%
Não fez	1	1%
**HIV**		
Negativo	94	94%
Positivo	5	5%
Não fez	1	1%
**Hepatite b**		
Negativo	98	98%
Positivo	1	1%
Não fez	1	1%
**Hepatite c**		
Negativo	99	99%
Não fez	1	1%
Positivo	-	-
**COMPORTAMENTOS VULNERÁVEIS AO HIV, SÍFILIS E OUTRAS IST'S**		
**Idade da 1ª relação sexual?**		
**Menor que 18 anos**	91	91%
13 anos	21	21%
12 anos	19	19%
14 anos	14	14%
15 anos	14	14%
17 anos	8	8%
16 anos	6	6%
11 anos	5	5%
10 anos	3	3%
9 anos	1	1%
**Maior ou igual a 18 anos**	9	9%
18 anos	6	6%
20 anos	2	2%
19 anos	1	1%
**Diagnóstico prévio de alguma IST?**		
NÃO	75	75%
SIM	25	25%
**Qual IST você já teve?**		
Sífilis	12	12%
Gonorreia	7	7%
Candidíase	5	5%

Tricomoníase	1	1%
**Utiliza preservativo em todas as relações sexuais?**		
NÃO	75	75%
SIM	25	25%
**Utiliza preservativo no sexo anal?**		
NÃO	35	35%
SIM	28	28%
AS VEZES	27	27%
NÃO FAZ	10	10%
**Utiliza preservativo no sexo vaginal?**		
NÃO	37	37%
SIM	32	32%
AS VEZES	31	31%
**Utiliza preservativo no sexo oral?**		
NÃO	45	45%
AS VEZES	25	25%
SIM	24	24%
NÃO FAZ	6	6%
**Razão para não utilizar preservativo?**		
Confiança	39	39%
Prefere pele na pele	23	23%
Não dá tempo de colocar	21	21%
Não se aplica	11	11%
Podem pensar que sou HIV positivo	4	4%
Preservativo não está disponível	1	1%
Reduz o prazer	1	1%
**Tipo de parceiro sexual?**		
Casual	52	52%
Esposo(a)	24	24%
Namorado(a)	18	18%
Trabalhador do sexo	6	6%
**Sexo com usuário de droga?**		
SIM	54	54%
NÃO	46	46%
**Relação sexual em troca de dinheiro?**		
NÃO	80	80%
SIM	20	20%
**Já apresentou algum sintoma de IST (corrimento, ardor, coceira)?**		
NÃO	57	57%
SIM	43	43%
**Tipos de sexo realizados.**		
Vaginal	99	99%
Oral	87	87%
Anal	81	81%
**Quantidade de parceiros?**		
Um	74	74%
Mais que um	26	26%
**Dor durante o ato sexual?**		
NÃO	95	95%
SIM	5	5%
**Sexo com parceiro com ferimento?**		
NÃO	86	86%
SIM	14	14%
**Parceiro com sintoma de IST?**		
NÃO	75	75%
SIM	25	25%
**Compartilhamento de perfurocortantes?**		
NÃO	95	95%
SIM	5	5%
**Reutilização de agulhas?**		

NÃO	95	95%
SIM	5	5%
**Já sofreu violência sexual?**		
NÃO	85	85%
SIM	15	15%
**Já sofreu violência física?**		
SIM	71	71%
NÃO	29	29%

Fonte: Da pesquisa,2019.

Quanto à vulnerabilidade ao HIV/aids mensurada através do questionário validado, a Tabela 5 demonstra a classificação.

Tabela 5. Classificação da vulnerabilidade ao HIV/aids de pessoas em situação de rua. João Pessoa, Paraíba, Brasil, 2018. N=100.

Variáveis	n	%
**Vulnerabilidade ao HIV/aids (questionário validado)**		
Alta	52	52%
Moderada	37	37%
Baixa	11	11%

Quanto as variáveis que indicam comportamentos vulneráveis a tuberculose, a Tabela 6 demonstra os resultados.

Tabela 6. Comportamentos vulneráveis a tuberculose de pessoas em situação de rua. João Pessoa, Paraíba, Brasil, 2018. N=100.

Variáveis	n	%
**Quantidade de banhos diários**		
Dois ou mais	72	72%
Um	28	28%
**Aspecto/aparência limpa**		
Sim	69	69%
Não	31	31%
**Escovação dos dentes**		
Mínimo uma vez ao dia	85	85%
Não escova	15	15%
**Exposição à chuva**		
Sim	63	63%
Não	37	37%
**Tosse**		
Não	80	80%
Sim	20	20%
**Secreção/expectoração amarelada ou esverdeada**		
Não	83	83%
Sim	17	17%
**Chiado no peito**		
Não	94	94%
Sim	6	6%
**Febre periodicamente**		
Não	88	88%
Sim	12	12%
**Tabagismo**		
Sim	83	83%

Não	17	17%
**Ritmo Respiratório**		
Eupneia	100	100%
**Ausculta respiratória (ruídos adventícios)**		
Não apresenta	92	92%
Roncos	7	7%
Sibilos	1	1%
Creptos	-	-

Fonte: Da pesquisa,2019.

Após análise por meio da TRI, o questionário referente aos comportamentos vulneráveis ao HIV, sífilis e outras IST's, previamente composto por 16 itens foi demonstrado que é capaz de mensurar esta variável através de nove questionamentos, conforme Tabela 7.

Pode-se verificar que todos os itens que foram aceitos apresentam dificuldade entre -2 e +2, exceto o compartilhamento de perfurocortantes e ter ~~sito~~ sido vítima de violência sexual, entretanto, apresentam boa discriminação, atingindo o valor desejado entre 0 e +2, além da carga fatorial superior a 0,30. Destaca-se ainda o valor de significância do instrumento por p>0,05, RMSEA de até 0,08. Esta análise confirma estes itens como imprescindíveis na mensuração da vulnerabilidade ao HIV e outras IST's em pessoas em situação de rua.

Tabela 7. Parâmetros da TRI, Carga Fatorial e índices de ajuste da Análise Fatorial para instrumento referente aos comportamentos vulneráveis ao adoecer relacionados à infecção ao HIV, sífilis e outras IST's de pessoas em situação de rua. João Pessoa, Paraíba, Brasil, 2018. N=100.

Itens	Discriminação (a)	Dificuldade (b)	Carga fatorial (F)
Diag. Prévio de IST's	0,917	1,402	0,473
Parceiro com sintoma de IST	1,032	-0,390	0,518
Uso de droga ilícita	1,007	-1,251	0,509
Cliente/parceiro usa droga	1,358	-0,172	0,624
Sexo em troca de Dinheiro	1,128	1,519	0,552
Sintomas de IST's	0,854	0,376	0,448
Quantidade de parceiro sexual	1,330	1,940	0,616
Compartilhamento de Perfurocortante	0,739	4,316	0,398
Vítima de Violência sexual	0,630	2,968	0,347
Variação % explicada	-	-	25,6
p-Valor	-	-	0,054
RMSEA	-	-	0,0691
RMSR	-	-	0,0926
TLI	-	-	0,7074
CFI	-	-	0,7805

Fonte: Da pesquisa,2019.

As Figura 9 e 10 demonstram a capacidade explicativa do fenômeno, pois apresenta formato aproximado de "s", revelando também a aptidão dos sujeitos do estudo.

A Curva Característica do Item, representado nas Figuras 9 e 10, do instrumento de comportamentos vulneráveis ao adoecer relacionados à infecção ao HIV, sífilis e outras IST's revela que todos os itens são capazes de explicar o fenômeno, pois apresentam curva sigmoide.

Figura 9. Curva Característica do Item do instrumento referente aos comportamentos vulneráveis ao adoecer relacionados à infecção ao HIV, sífilis e outras IST's de pessoas em situação de rua. João Pessoa, Paraíba, Brasil, 2018. N=100.

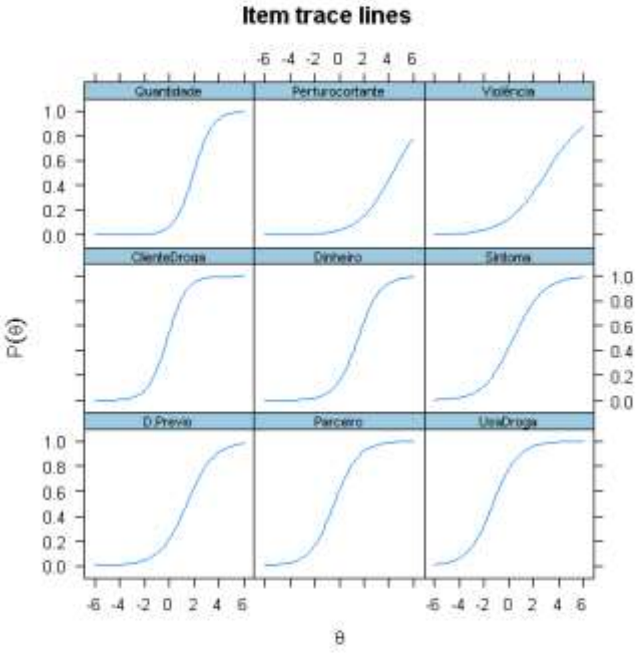

Fonte: Da pesquisa,2019.

Figura 10. Curva referente a todos os itens do instrumento de comportamentos vulneráveis ao adoecer relacionados à infecção ao HIV, sífilis e outras IST's de pessoas em situação de rua. João Pessoa, Paraíba, Brasil, 2018. N=100.

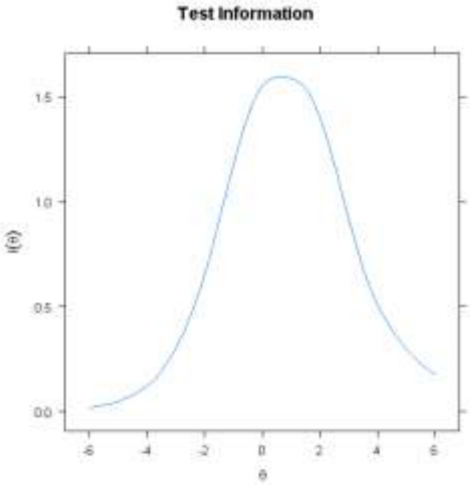

Fonte: Da pesquisa,2019.

A Tabela 8 demonstra a análise do instrumento referente à vulnerabilidade a tuberculose, totalizando oito itens que são capazes de avaliar esta variável por meio da TRI. Ressalta-se que todos os itens apresentam carga fatorial >0,30, exceto o diagnóstico prévio de doenças respiratórias. Destaca-se os valores de TLI e CFI >0,90, comprovando que este conjunto de itens são capazes de mensurar a vulnerabilidade à tuberculose em pessoas em situação de rua.

Tabela 8. Parâmetros da TRI, Carga Fatorial e índices de ajuste da Análise Fatorial para instrumento referente à vulnerabilidade a tuberculose de pessoas em situação de rua. João Pessoa, Paraíba, Brasil, 2018. N=100.

Itens	Discriminação (a)	Dificuldade (b)	Carga fatorial (F)
Escovação dentária precária	0,806	2,414	0,428
Exposição à Chuva	0,784	-0,769	0,418
Tosse	2,244	0,704	0,797
Chiado	4,475	1,666	0,935
Febre	1,211	2,047	0,580
Tabagismo	0,962	-1,933	0,492
Sons respiratórios com ruídos adventícios	2,328	1,765	0,807
Diagnóstico prévio de doenças respiratórias	0,460	2,501	0,261
Variação % explicada	-	-	39,6
p-Valor	-	-	0,2353
RMSEA	-	-	0,0459
RMSR	-	-	0,0811
TLI	-	-	0,9156
CFI	-	-	0,9697

Fonte: Da pesquisa,2019.

Embora o item diagnóstico prévio de doenças respiratórias não tenha apresentado valores ideais para discriminação, dificuldade e carga fatorial, este item deve permanecer no instrumento, pois apresentou curva aproximada da sigmoide, além de ser descrito em diversas literaturas como um fator de predisposição à tuberculose (FRANCO *et al.*, 2003).

Figura 11. Curva Característica do Item do instrumento de vulnerabilidade a tuberculose de pessoas em situação de rua. João Pessoa, Paraíba, Brasil, 2018. N=100.

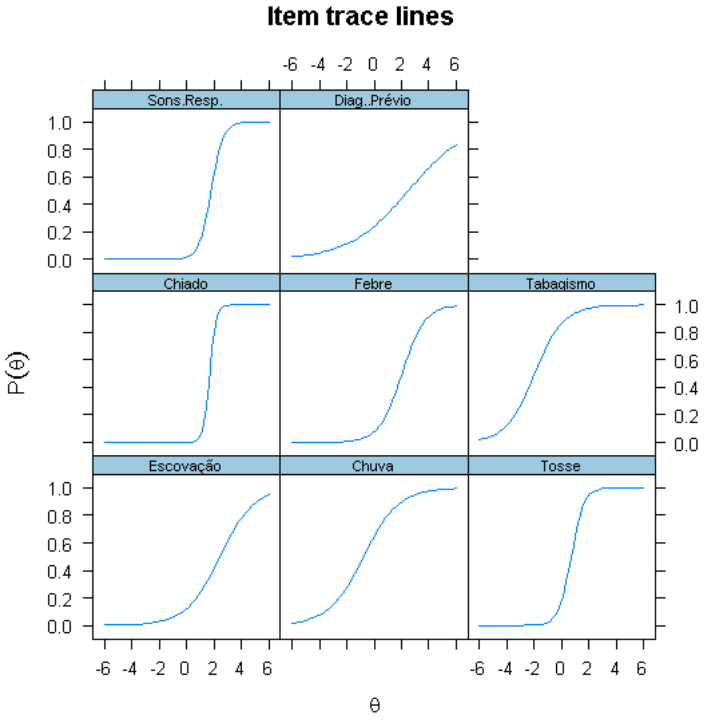

Fonte: Da pesquisa,2019.

Observa-se que a Curva Característica do Item comprova a eficácia dos itens do instrumento como capazes de mensurar a vulnerabilidade à tuberculose, conforme Figura 12.

Figura 12. Curva referente a todos os itens do instrumento de vulnerabilidade a tuberculose de pessoas em situação de rua. João Pessoa, Paraíba, Brasil, 2018. N=100.

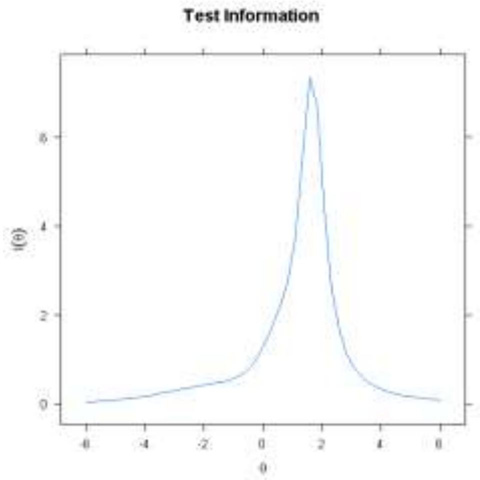

Fonte: Da pesquisa,2019.

O teste elaborado para o HIV apresentou Curva ROC com área 0,680 e intervalo de 95% de confiança compreendido entre 0,55 a 0,808. Para o ponto de corte (*Cuttoff point*) dois pontos foram candidatos ao melhor ponto de corte: (0,200; 0,234) e (0,60; 0,319). O primeiro foi descartado pela sua baixa sensibilidade. A Tabela 9 apresenta o ponto de corte para este teste e a Tabela 10 as propriedades diagnósticas para este ponto da curva ROC.

Tabela 9. Teste HIV elaborado com ponto de corte no escore igual a 4 confrontado com o padrão ouro (Teste ELISA) em pessoas em situação de rua. João Pessoa, Paraíba, Brasil, 2018. N=100.

Escore do Teste HIV	Teste padrão (ELISA)	
	Positivo	Negativo
Positivo ≥ 4	3	30
Negativo < 4	2	64

Fonte: Da pesquisa,2019.

Tabela 10. Propriedades do teste HIV com *cuttoff* igual a 4 para diagnóstico positivo de pessoas em situação de rua. João Pessoa, Paraíba, Brasil, 2018. N=100.

**Parâmetro**	**%**	**IC a 95%**
Sensibilidade	60,00	14,66 a 94,72
Especificidade	68,08	57,67 a 77,32
Acurácia	67,68	57,53 a 76,73
Valor preditivo positivo (VPP)	9,09	1,92 a 24,33
Valor preditivo negativo (VPN)	96,97	89,48 a 99,63

Fonte: Da pesquisa,2019.

Para o teste de Tuberculose, a área sob a curva ROC foi 0,783 com intervalo de confiança a 95% compreendido entre 0,667 a 0,900. O escore que determinou o ponto de corte é apresentado na Tabela 11 e as propriedades do teste estão na Tabela 12.

Tabela 11. Teste de Tuberculose elaborado com ponto de corte no escore igual a 3 confrontado com o padrão ouro de pessoas em situação de rua. João Pessoa, Paraíba, Brasil, 2018. N=100.

Escore do Teste Tuberculose	Teste padrão (diagnóstico autorrelatado)	
	Positivo	Negativo
Positivo ≥ 3	11	30
Negativo < 3	4	55

Fonte: Da pesquisa,2019.

Tabela 12. Propriedades do teste de Tuberculose com *cuttoff* igual a 3 para diagnóstico positivo de pessoas em situação de rua. João Pessoa, Paraíba, Brasil, 2018. N=100.

**Parâmetro**	**%**	**IC a 95%**
Sensibilidade	73,30	44,90 a 92,21
Especificidade	64,70	53,58 a 74,77
Acurácia	66,00	55,85 a 75,18
Valor preditivo positivo (VPP)	26,83	14,22 a 42,94
Valor preditivo negativo (VPN)	93,22	83,54 a 98,12

Fonte: Da pesquisa,2019.

Nas análises usando o WoE, foram identificados como principais fatores de risco para o HIV: desesperança, ansiedade, tempo de moradia de rua, idade, prática de sexo oral. A descrição da influência de fatores psíquicos, sociodemográficos e comportamentais sobre o HIV estão expressas nas Figuras 13,14,15,16.

Figura 13. Influência dos fatores psíquicos sobre o HIV de pessoas em situação de rua. João Pessoa, Paraíba, Brasil, 2018. N=100.

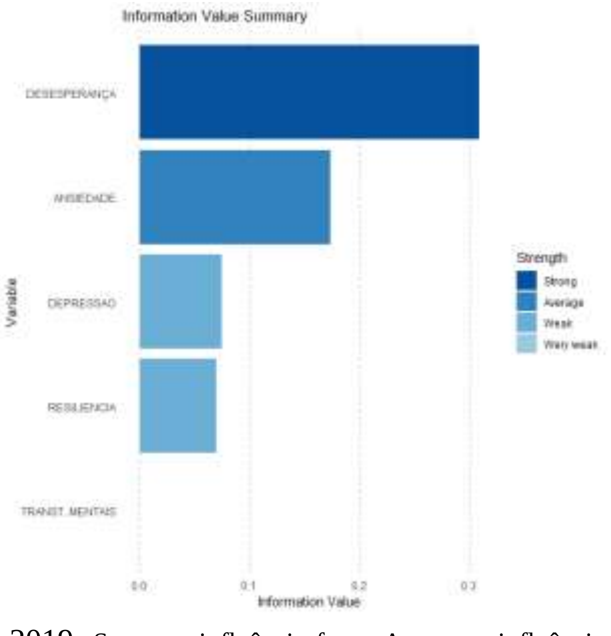

Fonte: Da pesquisa,2019. Strong = influência forte. Average= influência media. Weak= influência fraca. Very Weak= influência muito fraca.

Figura 14. Influência dos fatores sociodemográficos sobre o HIV de pessoas em situação de rua. João Pessoa, Paraíba, Brasil, 2018. N=100.

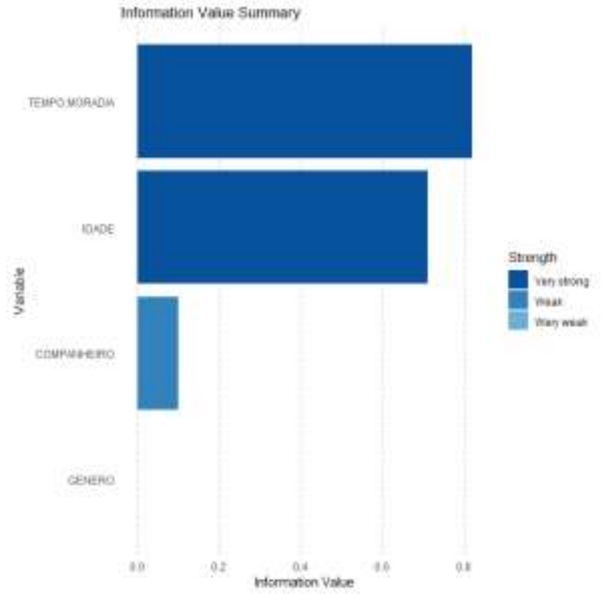

Fonte: Da pesquisa,2019. Very Strong = influência muito forte. Weak= influência fraca. Very Weak= influência muito fraca.

Figura 15. Influência dos fatores comportamentais sobre o HIV de pessoas em situação de rua. João Pessoa, Paraíba, Brasil, 2018. N=100.

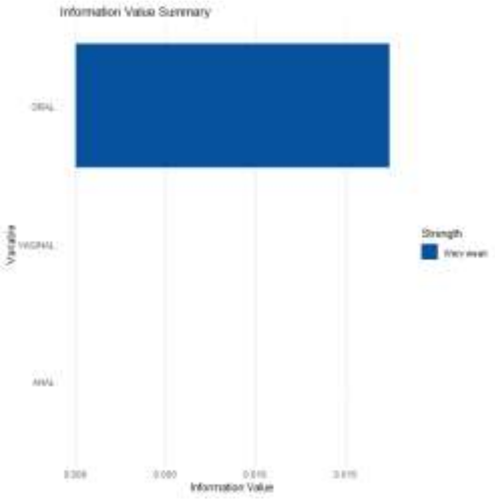

Fonte: Da pesquisa,2019. Very Weak= influência muito fraca.

Figura 16. Influência dos fatores (psíquicos, sociodemográficos, comportamentais) sobre o HIV de pessoas em situação de rua. João Pessoa, Paraíba, Brasil, 2018. N=100.

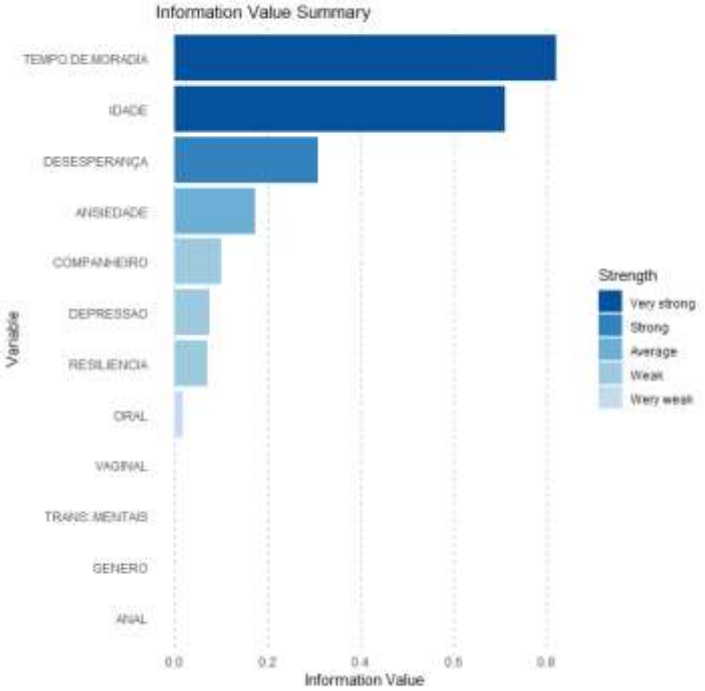

Fonte: Da pesquisa,2019. Very Strong = influência muito forte. Strong= influência forte. Average = influência média. Weak= influência fraca. Very Weak= influência muito fraca.

Nas análises usando o WOE, foram identificados como principais fatores de risco para a tuberculose: desesperança, baixa resiliência, tempo de moradia de rua, idade, sexo anal. Estas informações estão expostas nas Figuras 17,18,19 e 20.

Figura 17. Influência dos fatores psíquicos sobre a tuberculose de pessoas em situação de rua. João Pessoa, Paraíba, Brasil, 2018. N=100.

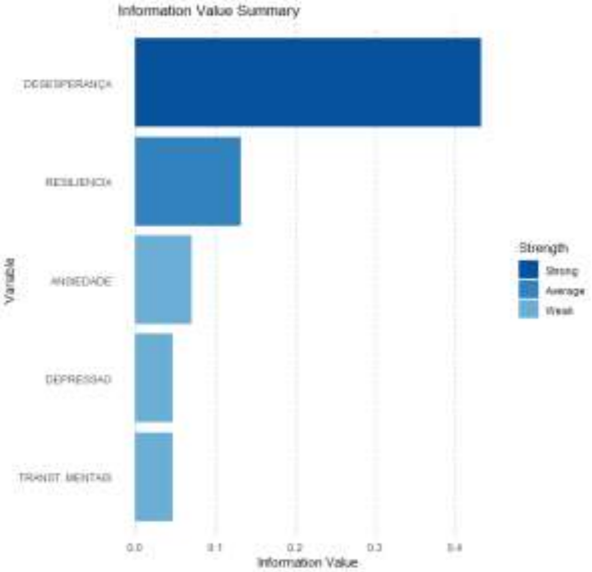

Fonte: Da pesquisa,2019. Strong= influência forte. Average= influência média. Weak= influência fraca.

Figura 18. Influência dos fatores sociodemográficos sobre a tuberculose de pessoas em situação de rua. João Pessoa, Paraíba, Brasil, 2018. N=100.

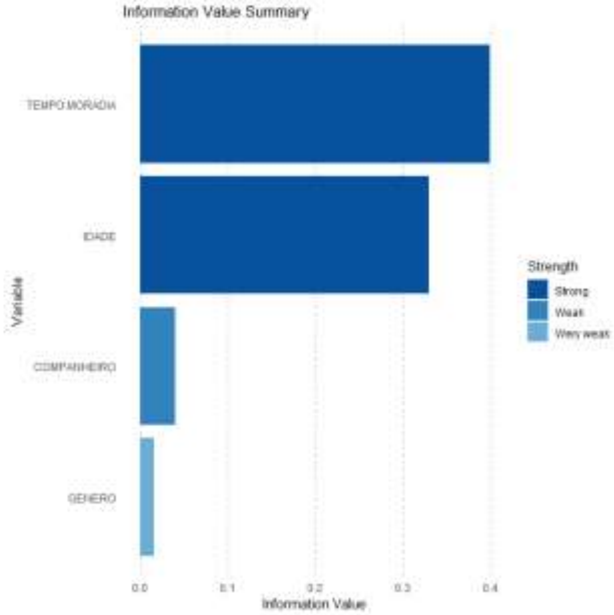

Fonte: Da pesquisa,2019. Strong= influência forte. Weak= influência fraca. Very Weak= influência muito fraca.

Figura 19. Influência dos fatores comportamentais sobre a tuberculose de pessoas em situação de rua. João Pessoa, Paraíba, Brasil, 2018. N=100.

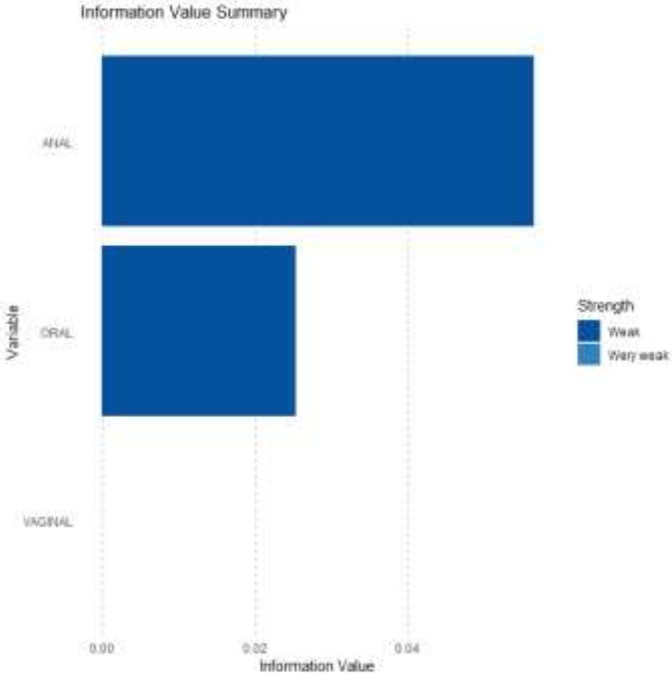

Fonte: Da pesquisa,2019. Weak= influência fraca. Very Weak= influência muito fraca.

Figura 20. Influência dos fatores (psíquicos, sociodemográficos, comportamentais) sobre a tuberculose de pessoas em situação de rua. João Pessoa, Paraíba, Brasil, 2018. N=100.

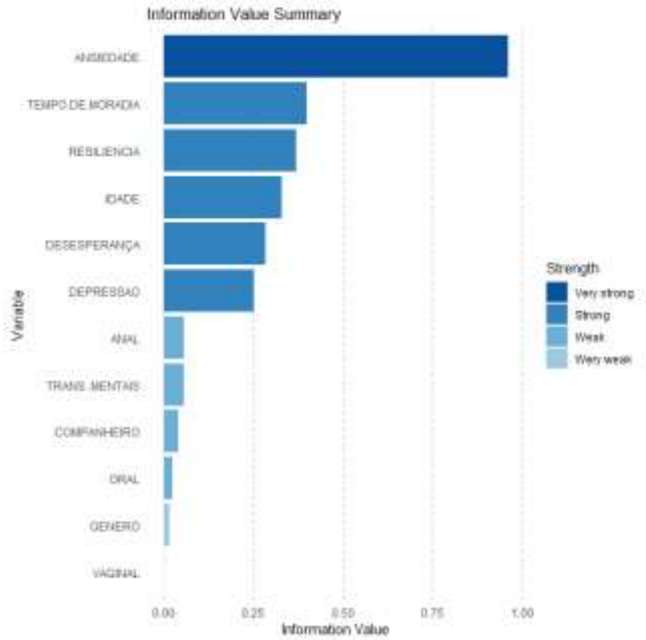

Fonte: Da pesquisa,2019. Very Strong = influência muito forte. Strong= influência forte. Weak= influência fraca. Very Weak= influência muito fraca.

O dendograma de ligação entre as variáveis/fatores permitiu distribuir as pessoas em situação deste estudo em três grandes grupos: desesperança e ansiedade; depressão, resiliência, transtornos mentais, prática de sexo vaginal e gênero; sexo oral, sexo anal, idade, tempo de moradia na rua e companheiro.

Figura 21. Dendograma que comprova o agrupamento de fatores psíquicos, sociodemográficos e comportamentais. João Pessoa, Paraíba, Brasil, 2018. N=100.

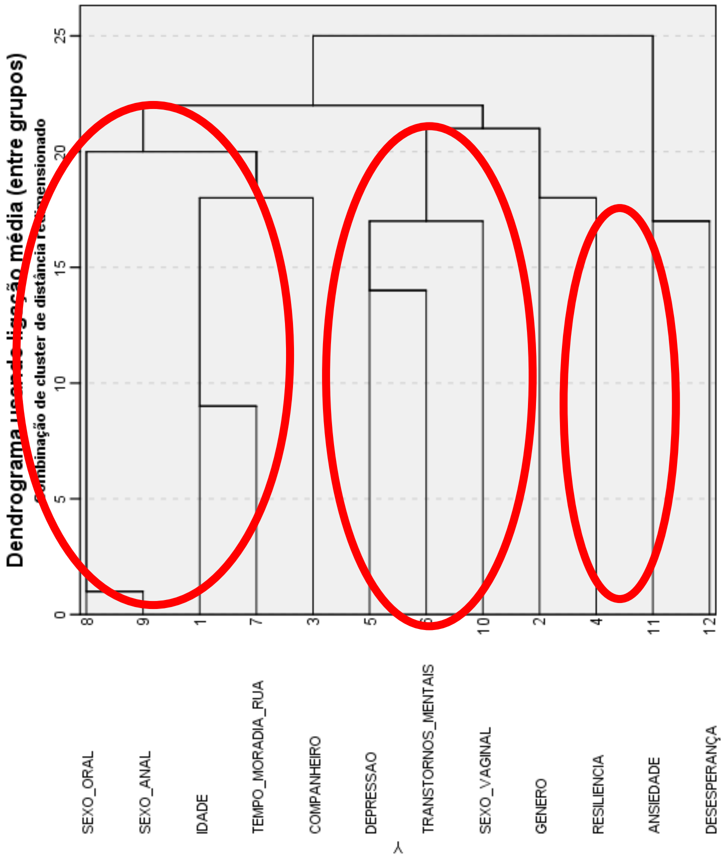

Fonte: Da pesquisa,2019.

O modelo de classes latentes, representado na Tabela 13, demonstra que três classes é o melhor modelo, pois apresenta o segundo menor valor de BIC e entropia relativa mais elevada ao comparar com o de duas classes.

Tabela 13. Modelo de classes latentes de pessoas em situação de rua. João Pessoa, Paraíba, Brasil, 2018. N=100.

Número de classes(k)	Parâmetros estimados	Graus de liberdade dos resíduos	Log-verossimilhança	AIC	BIC	G^2	X^2	Entropia relativa(%)
2	41	59	-874,58	1831,16	1937,97	847,53	54949,66	93,49%
3	62	38	-852,12	1828,24	1989,76	802,62	20878,59	95,89%
4	83	17	-816,67	1799,34	2015,57	731,72	17404,92	96,74%

Fonte: Da pesquisa,2019. AIC = Critério de Informação Akaike. BIC= Critério de Informação Bayesiano. G^2= Razão de verossimilhança. X^2 = qui-quadrado.

Desta forma, optou-se por eleger o modelo de três classes latentes e classificar os indivíduos como alto, médio e razoável risco para adoecimento. Foi escolhido três classes,

pois anteriormente foi averiguado que os sujeitos desta pesquisa dividem-se em três categorias, além disso, considerando o valor de AIC como o menor do modelo.

Para entender quais são as características dos sujeitos do estudo que pertencem a cada classe realizou-se a interpretação do LCA, como demonstra a Tabela 14.

Tabela 14. Interpretação das classes latentes de pessoas em situação de rua. João Pessoa, Paraíba, Brasil, 2018. N=100.

Classe	Idade	Gênero	Companheiro	Resiliência	Depressão	Transtorno Mental	Tempo de Moradia	Oral	Anal	Vaginal	Ansiedade	Desesperança
1	30 a 40	Masculino	Solteiro	Baixa e média	Sim	82% sim	>5anos	Sim	Sim	Sim	Leve	Moderada
2	>40	Masculino	Solteiro	Baixa, média e alta	Sim	57% sim	Exceto 2 a 5 anos	Não	Não	Sim	Mínima	Mínima
3	≤30	Masculino	Solteiro	Baixa	Sim	85% sim	0 a 1 ano	Sim	Sim	Sim	Leve	Leve

Fonte: Da pesquisa,2019.

Sendo assim, classificou-se como características dos riscos relacionados ao adoecimento de pessoas em situação rua: alto risco a classe 1, risco médio a classe 3 e risco razoável a classe 2.

Verificação da estrutura fatorial dos fatores sociodemográficos, psíquicos e comportamentais que influenciam a vulnerabilidade ao HIV e outras IST's através da análise fatorial confirmatória, como revela Tabela 15 e Figura 22.

Tabela 15. Análise fatorial confirmatória referente a categorias de risco à saúde de pessoas em situação de rua. João Pessoa, Paraíba, Brasil, 2018. N=100.

CFI	TLI	Gl	p(X²)	RMSEA	IC RMSEA	SRMR
0,986	0,982	51	0,383	0,022	0.000 0.069	0,075

Fonte: Da pesquisa,2019.

A Figura 22 demonstra que não há interação entre as variáveis de uma categoria com outra, comprovando sua distinção e correta classificação das classes psíquica, sociodemográfica e comportamental, além disso apresenta cargas fatoriais que demonstram a significância das variáveis de cada categoria.

Figura 22. Análise fatorial confirmatória das categorias de vulnerabilidade ao adoecimento de pessoas em situação de rua. João Pessoa, Paraíba, Brasil, 2018. N=100.

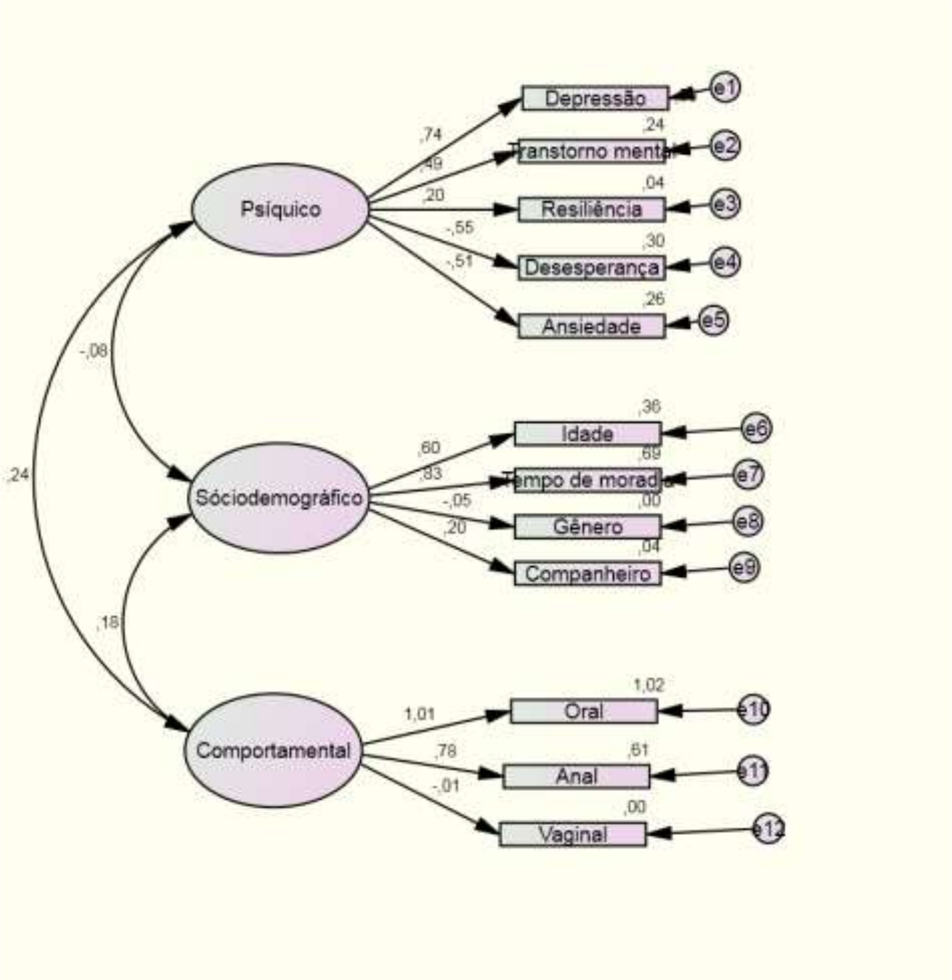

Fonte: Da pesquisa,2019.

A figura 22 demonstra cargas fatoriais entre as variáveis observadas e os fatores, sendo valores >0,30 estatisticamente significativos, como entre os fatores psíquicos que encontram-se os transtornos mentais e a depressão; entre os sociodemográficos a idade e tempo de moradia; entre os comportamentais o sexo oral e anal, sendo estes os fatores que mais influenciam no adoecimento de pessoas em situação de rua.

7 DISCUSSÃO

A discussão encontra-se subdivida em tópicos:

1. Características sociodemográficas e clínicas
2. Saúde mental/fatores psíquicos
3. Testes rápidos, comportamentos e fatores que predispõem a vulnerabilidade ao HIV, IST's e tuberculose

7.1Características sociodemográficas e clínicas

Observa-se que a maioria das pessoas em situação de rua deste estudo são solteiros, em sua maior parte homens, sendo estas variáveis similares a outros estudos realizados na região Sul e Centro-Oeste do Brasil que encontraram maior prevalência de homens, solteiros, entre 24 a 60 anos, onde em sua maioria teve no mínimo um filho (WINKELMANN *et. al*., 2018; LAWDER *et. al*., 2019).

Dentre os motivos que levaram as pessoas a estarem em situação de rua o confronto familiar e o uso de drogas obteve maior prevalência. A fragilidade nos laços familiares torna mais difícil o processo de viver nas ruas, gerando insegurança, medo, solidão, tristeza e desesperança. Ressalta-se ainda que o consumo excessivo de drogas contribui para redução de expectativa de vida destas pessoas (ALDRIDGE *et al*., 2018; FIORATI *et al*., 2016).

Quanto ao serviço de saúde a maior parte das pessoas em situação de rua não o procuram, fato justificado pela ausência de endereço fixo e documentação de identificação, dados exigidos para o atendimento na rede de atenção à saúde gratuita. Além disso, ressalta-se a ênfase no modelo biomédico medicalizador que trata apenas a doença e não promove prevenção de doenças ou promoção da saúde. Pesquisa realizada com a Teoria das Representações Sociais com pessoas em situação de rua detectou a figura do profissional médico como o centro da assistência à saúde, comprovando ainda mais o cuidado direcionado a cura, atuando na legitimação do modelo higienista (SILVA *et al*., 2018).

Tratando-se de acesso a saúde, vale lembrar que as pessoas em situação de rua podem contar com a assistência das Equipes de Consultório de Rua que possuem profissionais médicos, odontólogos e enfermeiros, que oferecem procedimentos de atenção primária e encaminham para outros setores conforme necessidade. Além disso, estas equipes trabalham na perspectiva da prevenção de doenças e promoção da saúde (BORYSOW, 2017).

Destaca-se ainda a Política Nacional para a População em Situação de Rua que defende o cumprimento dos princípios do Sistema Único de Saúde para este público, reconhece as características intrínsecas às pessoas em situação de rua e estimula ações de promoção da saúde e articulação com os demais serviços (BRASIL, 2009).

Os dados da presente pesquisa apontam alta prevalência de diabetes e hipertensão, doenças existentes na população geral, mas que tornam ainda mais preocupantes em pessoas em situação de rua, uma vez que o contexto em que vivem potencializa outras consequências cardíacas. Pesquisa realizada com pessoas em situação de rua na Itália evidenciou que as condições de saúde estão diretamente associadas a presença de doenças crônicas, déficit de atividade física e uso de medicamentos, fatos que podem ser evitados e/ou tratados através do livre acesso aos serviços de saúde (ALAGNA *et al*., 2019).

As doenças cardiovasculares estão entre as principais causas de morte entre as pessoas em situação de rua, sendo alguns comportamentos influenciadores nesta estatística, como a depressão, ansiedade, consumo de drogas, cigarro e etilismo (BAGGETT *et al*., 2018). Fatores com elevada prevalência neste estudo são um dos elementos que contribuem para a permanência nas ruas como problemas familiares, falta de apoio social, histórico de abuso físico, contribuindo para o desenvolvimento de comportamentos negativos (PANADERO *et al*., 2017; BARMAN *et al*., 2016).

Estas doenças prévias tornam-se ainda mais preocupantes em pessoas em situação de rua, pois embora muitas vezes diagnosticados, não recebem tratamento oportuno ou medidas de controle/redução de agravos relacionados aos fatores que predispõem ao adoecimento como o sobrepeso, alimentação inadequada, uso de substâncias químicas, doenças psiquiátricas, violência física e sexual e estresse, como afirmado por Axon (2016) e Lebrun-harris *et al*. (2013), podendo gerar sintomatologias como dor, tristeza e cefaleia.

Ressalta-se também, a prevalência de consumo de drogas e álcool na presente pesquisa, corroborando com estudo com PSR de São João Del Rei,Minas Gerais, Brasil que objetivou compreender as vulnerabilidades de adultos em situação de rua ao comportamento suicida (CASTRO *et al*., 2019).

No contexto de PSR, pesquisa de representações sociais evidenciou que o consumo de drogas e uso de álcool está associado a: exclusão, viver no risco, discriminação, pobreza, fome (CAMPOS *et al*., 2019).

O consumo de drogas entre PSR torna-se cada vez mais prevalente, sendo motivo de preocupação entre gestores de saúde pública, pois aumentam a exposição da vulnerabilidade

ao adoecimento envolvendo violência, crimes, tráfico, venda do sexo, detenção, roubos, assaltos (TOLEDO *et al*., 2017).

Desta forma, uma pesquisa nos Estados Unidos, revelou duas estratégias/intervenções para minimizar a problemática do álcool em PSR, que inclui o Modelo de Gerenciamento de Casos e o Modelo de Recuperação. O primeiro é subdividido em quatro estratégias: casos padrão que estabelece suporte durante todo o atendimento mas possui tempo limitado; intensiva de casos que não possuem tempo limitado e inclui contatos mais frequentes com o paciente; no comunitário assertivo o acompanhamento é 24 horas através de uma equipe multidisciplinar; intervenção crítica no tempo é colocado em prática quando o indivíduo encontra-se em estado crítico e existe uma comunicação entre os serviços com níveis de atenção elevados (ALESSANDRINI *et al*., 2018; VET *et al*., 2013).

Acrescenta-se que o modelo de recuperação abrange pilares que são capazes de estruturar a recuperação de pessoas em situação de rua que encontram-se em abuso de substâncias: saúde (superar ou administrar), casa (possuir lugar seguro para moradia), finalidade (realizar atividade diárias – trabalho, estudos) e comunidade (relacionamentos e redes sociais) (ALESSANDRINI *et al*., 2018; VET *et al*., 2013).

7.2 Saúde mental/fatores psíquicos

Nesta tese foi encontrado, com maior representatividade, a presença de baixa resiliência, ansiedade leve, transtornos mentais comuns e depressão. Entender o processo de resiliência abrange a necessidade de desvencilhar e encontrar alternativas para superar adversidades da melhor maneira possível, sendo potencializado quando há um ambiente que favorece a construção de novas estratégias para o enfrentamento. Desta forma, pessoas em situação de rua tendem a apresentarem resiliência comprometida, pois não conseguem encontrar um ambiente propício e apresentam fragilidades no relacionamento com família e amigos (UNGAR, 2013).

Existem três medidas que influenciam diretamente a resiliência, que são a autoestima, autoeficácia e controle interpessoal, como dito por Windle et al (2008), tornando difícil possuir estas características no contexto em que a PSR vive, pois este ambiente apresenta julgamento, discriminação e solidão, favorecendo sentimento de tristeza e desesperança, contribuindo para a fragilidade na resiliência.

Estudo realizado em Cheshire demonstrou que tornar-se desabrigado é um processo que influência a resiliência de forma negativa e progressiva que por sua vez associa-se a

discriminação que é justificada pelo abuso de substâncias, alcoolismo, autoflagelação, roubo, entre outros (MABHALA *et al*., 2017).

Pesquisa com pessoas em situação de rua encontrou características importantes que estimulam a resiliência, como ter perspectiva de futuro, boa identidade pessoal, apoio social através da inclusão, apoio familiar, acesso a recursos materiais e moradia segura (TOZER *et al*., 2015; UNGAR *et al*., 2008). Além disso, outro estudo revelou que quanto maior a resiliência menores são as chances de consumo drogas neste público (UNGAR, 2004).

Referindo-se aos transtornos mentais, destaca-se que os mais frequentes na população geral, assim como nas PSR são a ansiedade e a depressão (HUSAIN *et al*., 2008; LAPORTE *et. al*., 2018).

Pesquisa com PSR na Finlândia, que examinou a prevalência de transtornos mentais em pessoas em situação de rua encontrou resultados similares a este estudo, em que a maioria possuíam-no (89,2%). Ainda, nesta mesma pesquisa, encontrou-se que o principal motivo de procura de assistência na atenção primária eram relacionadas à saúde mental e uso de substâncias, seguido por infecções e traumas (STENIUS-AYOADE *et al*., 2017).

Diante de todas estas características relacionadas à saúde mental, uso de drogas, dificuldade de atendimento pelo serviço de saúde, abandono dos familiares, entre outros, as pessoas em situação de rua sentem-se com a esperança fragilizada, assim como revelado por 29% dos voluntários desta pesquisa. Entretanto, a esperança é considerada uma válvula de escape que impulsiona e motiva estas pessoas a procurarem melhores condições de vida, recomeçarem suas vidas, buscarem educação, emprego e reconstruir laços futuros (KIRST *et al*., 2014).

Destaca-se que a desesperança direciona o indivíduo para o sofrimento, angústia, baixa qualidade de vida, suicídio e comprometimento da resiliência (HUGHES *et al*., 2010).

A depressão, por sua vez, encontra-se presente nas pessoas em situação de rua desta pesquisa e de outras, como a de Botti *et al* (2010) e Irwin *et al* (2008) que por sua vez é explicada pela solidão, abandono, estresse e dificuldades diárias.

Por fim, é possível compreender que as pessoas em situação de rua apresentam saúde mental frágil, favorecendo comportamentos vulneráveis à saúde, uma vez que não apresentam esperança de dias melhores, potencializando as chances de adoecimento.

7.3 Testes rápidos, comportamentos e fatores que predispõem a vulnerabilidade ao HIV, IST's e tuberculose

Esta pesquisa detectou prevalência e comportamentos que influenciam IST's, assim como, HIV e outras IST's.

Pesquisas de metanálise e outra realizada com PSR de diversos lugares dos EUA revelaram a prevalência de 6,3% a 21,1% de HIV, respectivamente (BEIJER *et. al.*,2012; STREHLOW *et. al.*,2012). Quando a hepatite C 3,9% a 36,2% e hepatite B 32,4% em PSR segundo Klinkenberg *et al* (2003), sendo todos estes valores superiores quando comparados ao presente estudo que encontrou 5% para HIV, 29% sífilis, 1% hepatite B.

Torna-se importante destacar os comportamentos vulneráveis às infecções ao HIV e outras IST's presentes neste estudo e que corroboram com outras pesquisas com pessoas em situação de rua, como início da relação sexual precoce, diagnóstico prévio de IST, fragilidade no uso do preservativo, parceiro sexual casual, uso de drogas, sexo em troca de dinheiro. Além disso, Barros (2018), afirma que no Brasil, que a prevalência de sífilis, nesta população, está relacionada a baixa escolaridade, sexo sob efeito de drogas, úlcera genital prévia e outras IST's, além do quantitativo de parceiros sexuais.

Outro item encontrado na presente pesquisa é a relação sexual em troca de dinheiro, sendo caracterizada muitas vezes como hábito em pessoas em situação de rua e pode estar associada ao consumo de drogas, tornando esta prática extremamente vulnerável a IST's. Ressalta-se também que o dinheiro adentra neste processo por motivo de sobrevivência, em troca de alimentação, higiene e necessidades fisiológicas básicas (BOSKA *et al.*, 2017; URADA *et al.*, 2019).

Desta forma, comportamentos vulneráveis a infecção ao HIV e outras IST's, praticados por pessoas em situação de rua, também foram evidenciados em outra pesquisa incluindo múltiplos parceiros sexuais, compartilhamento de objetos pessoais, assédio sexual, além da fragilidade no conhecimento sobre gonorreia e HIV (DESMENNU *et al.*, 2018).

O entendimento da relação entre HIV e as práticas inseguras, como o uso de drogas injetáveis, profissionais do sexo, transexuais, vem sendo estudados e categorizados como de maior risco para o HIV (MIMIAGA *et al.*, 2015; MTHEMBU *et al.*, 2017).

Na pesquisa em lide, houve predominância de consumo de drogas ilícitas e sabe-se que o sexo com usuários de drogas contribui para o aumento da infecção ao HIV/aids, pois influencia diretamente no uso do preservativo, além de estar relacionado a violência física e/ou sexual (SOPHEAB *et al.*, 2018).

A violência sexual, também presente neste estudo, torna-se prevalente devido a muitas mulheres em situação de rua serem coagidas, forçadas a realizarem esta prática, justificadas pela sua fragilidade emocional e financeira, contribuindo também para deixar de lado a negociação do sexo seguro, aumentando a chance de infecção pelo HIV/aids (ALLAHQOLI *et al*., 2014). Quanto as estratégias para minimizar as IST's, na população em situação de rua, pesquisa revela três prioridades citadas por este público: organização de grupos de apoio, grupos de aconselhamento e assistência (emprego), educação para mudança de comportamento, sendo todos estes em parceria com o governo (DESMENNU *et al*., 2018).

Diversos estudos indicam que pessoas com saúde mental comprometida, que fazem uso de álcool, possuem múltiplos parceiros sexuais e compartilham agulhas apresentam maior risco de infecção ao HIV e outras IST's (RAMIRO *et al*., 2013; HILL *et al*., 2018; BEKELE *et al*., 2018; SSEWAMALA *et al*., 2018; COOK *et al*., 2018). Desta forma, tornando as variáveis do instrumento validado pela TRI e os fatores de risco encontrados pelo WoE, como fidedignas na mensuração de vulnerabilidade ao HIV e outras IST's em pessoas em situação de rua.

O sexo em troca de dinheiro, uso de drogas e desuso do preservativo realizados pela amostra desta pesquisa, merece destaque, pois pessoas vivendo com HIV que realizam estas práticas apresentam carga viral detectável, aumentando a possibilidade de transmissão (DASGUPTA *et al*., 2019). Ressalta-se ainda que o uso de drogas injetáveis aumentou a transmissão do HIV entre pessoas em situação de rua mesmo em cidades que possuem programas de prevenção (GOLDEN *et al*., 2019).

Ainda referente a validação dos questionários sobre HIV, outras IST's e tuberculose, ressalta-se que a curva ROC detectou pontos de corte e seus parâmetros diagnósticos que são capazes de rastrear a probabilidade de, verdadeiramente, se ter a doença quando comparados ao teste padrão ouro. Isto, torna-se relevante a medida em que pode ser utilizado como método de detectar ou descartar a probabilidade de ter ou não a doença.

A influência da desesperança no diagnóstico de HIV dá-se ao fato da difícil convivência e aceitação deste diagnóstico, a incerteza do futuro, sentimento de culpa e ideação suicida. Destaca-se o apoio social e a perspectiva de enxergar o HIV como uma patologia e não como uma doença moral, como alternativa de reconstrução da esperança (GARCIA; RAMOS, 2017).

A desesperança é considerada um fator que predispõe a ideação suicida em pessoas com HIV (DUKO *et al*., 2019). O diagnóstico de HIV em outra pesquisa com pessoa em

situação de rua mostrou-se associado ao sentimento de morte devido a estigmatização e discriminação (ANTUNES *et al*., 2016)

Além disso, ressalta-se que a ansiedade está fortemente relacionada ao HIV, revelando abandono na terapia antirretroviral, qualidade de vida prejudicada e aumento na mortalidade (TAO et al., 2017). Mas, o apoio social é indicado como um grande protetor da ansiedade nestas pessoas (WANG *et al*., 2014).

O sexo oral, identificado nesta pesquisa como influenciador do HIV, merece atenção, pois esta prática apresenta certa invisibilidade de risco de transmissão de IST's e normalmente é realizada na ausência de preservativo (DIAZ *et al*., 2013).

No Brasil, de acordo com a Política Nacional de Pessoas em Situação de Rua há a necessidade de assegurar políticas de saúde, bem como sua articulação com os setores de assistência social (BRASILa, 2009). Destaca-se ainda que o Ministério da Saúde considera as pessoas em situação de rua como grupo vulnerável ao HIV e a tuberculose, sendo esta última patologia com risco 56 vezes maior quando comparados a população geral. Além disso, refere que deve-se rastrear/suspeitar/diagnosticar a tuberculose em pessoas em situação de rua independentemente do tempo de tosse (BRASIL, 2019).

Adiciona-se a isso, o definido na Resolução MS 02/2013 que estabelece diretrizes e estratégias para o enfrentamento das iniquidades e desigualdades com foco nas PSR, que afirma o compromisso das três esferas com cinco eixos, sendo o segundo referente a busca ativa e tratamento supervisionado de doenças infecciosas como tuberculose, IST's, HIV/aids, assim como, a redução dos casos, além de assegurar acesso a vacinação (BRASILb, 2013).

Quanto a tuberculose, ressalta-se pesquisa realizada em João Pessoa/PB que aponta que a forma como se vive nas ruas contribui para o adoecimento por tuberculose e que, uma vez diagnosticado a adesão ao tratamento se torna frágil (BARBOSA, 2018).

Nota-se que a tuberculose permanece como de difícil controle em pessoas em situação de rua devido a aglomeração, diagnóstico tardio, fragilidade na adesão ao tratamento e é considerada um dos fatores importantes que contribuem para a mortalidade destas pessoas (GOETSCH *et al*., 2012; KHAN *et al*., 2011). Além disso, ressalta-se a associação do abuso de álcool, tabagismo e infecção por HIV com o elevado índice de tuberculose (VIEIRA *et al*., 2018).

Pesquisa de Heuvelings et al (2017) demonstrou estratégias eficazes para o tratamento e diagnóstico da tuberculose em pessoas em situação de rua, como a triagem, através da radiografia de tórax com equipamento móvel e exame do escarro, além da terapia diretamente observada.

Estudo realizado com PSR da Alemanha revelou que dentre os fatores de risco para tuberculose em nestas pessoas encontra-se a baixa escolaridade (VON *et al.*, 2019). Outrossim, a vulnerabilidade à tuberculose é sensível a fatores climáticos como o aumento da temperatura, conforme afirma Cao *et al*. (2016) que favorece a atividade das bactérias e melhoram sua viabilidade que por sua vez está diretamente relacionada ao expressivo número de mortes por tuberculose durante o verão (PRUDENCIO *et al*., 2016).

Sendo assim, o presente estudo ao encontrar variáveis capazes de medir a vulnerabilidade a tuberculose, como exposição à chuva, encontra-se de acordo com achados de que o clima interfere significativamente na prevalência da tuberculose.

Uma pesquisa de Nanzaluka *et al*. (2019) que buscou encontrar desfechos desfavoráveis para tuberculose detectou o tabagismo como um fator importante, sendo o mesmo considerado como um dos itens capazes de verificar a vulnerabilidade a tuberculose em pessoas em situação de rua validado nesta pesquisa através da TRI.

Outro item que pode predispor a tuberculose é a higiene bucal precária, fato rotineiro no contexto de pessoas em situação de rua assim como afirmado por Hwang e Quiñez (2013), bem como nesta pesquisa, que encontraram necessidade de escovação, restauração dentária e tratamento periodontal.

As pessoas em situação de rua apresentam características específicas que favorecem o adoecimento por tuberculose, como comprovado pela WoE que encontrou fatores, como: desesperança, baixa resiliência, tempo de moradia de rua, idade, sexo anal e oral.

A desesperança e o comprometimento da resiliência estão presentes constantemente na vida de pessoas em situação de rua, e ainda mais potencializado se estão com tuberculose. A incerteza do tratamento e da cura, o ambiente que não favorece e a assistência nem sempre acolhedora, torna a recuperação lenta, podendo ocorrer o abandono do tratamento. Para assistir estas pessoas com tuberculose se faz necessário muito mais do que apenas uma equipe médica, é preciso de serviços sociais, capazes de garantir o básico para manutenção das necessidades humanas básicas (CHENG *et al*., 2015). Outra pesquisa realizada em São Paulo, Brasil, afirma que, enquanto não houver implementação de direitos básicos para as pessoas em situação de rua, será quase impossível conseguir erradicar a tuberculose (ALECRIM *et al*., 2016).

Ainda, outra pesquisa realizada em Teerã, Irão, definiu que as condições de moradia e acesso limitado a serviços de saúde influenciam diretamente na infecção por tuberculose, podendo este fato está associado ao tempo de moradia de rua (AMIRI *et al*., 2018).

A infecção por tuberculose pode aumentar em 8% conforme se envelhece a cada ano. A tuberculose pode estar associada ao uso de drogas, condições sociais e financeiras precárias, e além disso, a atividade sexual (ZHANG *et al*., 2013).

Os casos de tuberculose transmitida por via sexual, normalmente, ocorrem em pessoas que tiveram ou têm um diagnóstico de TB pulmonar. Após uma recidiva, o *Mycobacterium tuberculosis* pode infectar a área genital, sendo de fundamental importância que ao diagnosticar o indivíduo com TB genital, o seu(a) parceiro(a) seja investigado, com o objetivo de fornecer tratamento idêntico e resolutivo. Além disso, ressalta-se que a TB genital provoca fibrose nos órgãos genitais, ocasionando a infertilidade (KIMURA *et al*., 2018).

Registra-se que em 2018, foi diagnosticado o primeiro caso de tuberculose genital, em estudo realizado por Kimura et al. (2018). Assim, esta tese torna-se relevante, à medida que encontrou, um ano após a referida pesquisa, a influência da relação sexual com esta doença.

Desta forma, ao constatar que o sexo por via oral e anal exercem influência nos casos de tuberculose, aponta para a necessidade de se investigar profundamente as suspeitas de TB genital, e para além disso, solicitar que o indivíduo com TB tenha abstinência sexual, ao menos na fase de transmissão da doença.

Referente ao atendimento em saúde, a Resolução MS 02/2013 estabelece o comprometimento em capacitar os funcionários em busca de fornecer uma atenção acolhedora e qualificada, sensibilizando-se com as PSR, tendo a inserção de temáticas relevantes no contexto de PSR como obrigatório no currículo nos cursos de formação em saúde (BRASILb, 2013).

Ressalta-se que os consultórios de/em rua devem prestar assistência as pessoas em situação de rua, entretanto, não se configuram como único acesso ao Sistema Único de Saúde, o atendimento pode ser realizado em Unidades Básicas de Saúde (UBS), Unidades de Pronto Atendimento, Centros de Atenção Psicossocial, Serviço de Atendimento Móvel de Urgência (BRASILa, 2011; BRASIL, 2017).

Ainda esclarece-se que as UBS’s são responsáveis pelo cuidado integral da população em situação de rua, além de atuar na vigilância sanitária considerando fatores ambientais e epidemiológicos, culturais e socioeconômicos (BRASIL, 2017).

Outro aspecto importante é que o atendimento pelo Sistema Único de Saúde não pode ser negado devido à falta de endereço, documento de identificação e cartão SUS conforme estabelece a Portaria MS 940/2011 (BRASILc, 2011).

Dentre as estratégias de enfrentamento da tuberculose em pessoas em situação de rua estão: incentivos materiais, acesso a habitação, cuidados à saúde centrados na PSR, práticas socioeducativas com participação ativa da comunidade (BRASIL, 2016).

Sendo assim, estes resultados trazem implicações para a prática de profissionais de saúde e gestores, no sentido de aprimorar a Política Nacional para Pessoas em Situação de Rua, incluindo temas específicos como HIV e tuberculose, enfatizando de forma minuciosa que além deste atendimento ser garantido pelo SUS, é preciso ressaltar que fatores influenciam estas doenças, quais melhores métodos de investigá-las, como acolher melhor estas pessoas. Pois, uma vez que se sabe que existem aspectos sociodemográficos, psíquicos e comportamentais que influenciam estas duas doenças, torna-se essencial incluí-los na busca ativa e anamnese dos órgãos que atendem estas pessoas, como os CENTRO POP e os consultórios de/em rua.

8 CONCLUSÃO

Dentre os objetivos desta tese tem-se a caracterização das pessoas em situação de rua, sendo encontrado o confronto familiar e uso de drogas como responsáveis pelo maior quantitativo entre os motivos para estarem na rua. Além disso, a maioria não procura serviço de saúde, tiveram tuberculose e pneumonia, são etilistas, utilizam drogas ilícitas em sua maior parte maconha e crack. Apresentaram transtornos mentais comuns, além de ansiedade, desesperança, baixa resiliência e depressão.

No aspecto relacionado aos comportamentos vulneráveis a infecção ao HIV, sífilis e outras Infecções Sexualmente Transmissíveis, bem como Tuberculose de pessoas em situação de rua, encontrou-se casos de sífilis e HIV para os testes rápidos realizados. Destaca-se a relação sexual precoce, sexo inseguro sem preservativo, sendo o principal motivo a confiança. Praticam sexo com parceiro casual, alguns em troca de dinheiro. Relataram violência sexual e física, exposição à chuva e tabagismo.

Foram validados itens importantes quanto a vulnerabilidade ao HIV e outras IST’s como o diagnóstico prévio e parceiro com sintoma de IST’s, uso de drogas ilícitas pelo indivíduo e pelo parceiro, compartilhamento de perfurocortantes e violência sexual. Além disso, identificou-se itens que predispõe a vulnerabilidade à tuberculose, como a escovação dentária, exposição à chuva, tosse, chiado, febre, tabagismo, sons respiratórios com ruídos adventícios e diagnóstico prévio de doenças respiratórias.

Observou-se ainda, na amostra, a presença de três classes/fatores que influenciam o HIV e a tuberculose: sociodemográficos, psíquicos e comportamentais. Logo, a desesperança, ansiedade, depressão, resiliência, tempo de moradia de rua, idade e o sexo oral exercem influência sobre o diagnóstico de HIV. Quanto à tuberculose foram encontrados que, a desesperança, resiliência, ansiedade, tempo de moradia na rua, idade, prática de sexo anal e oral influenciam-na.

Pessoas em situação de rua apresentam condições de saúde que podem provocar adoecimento e exigem transformações dos sistemas de saúde pública, de modo a inseri-las em uma assistência integral, através de um atendimento qualificado.

No tocante às doenças transmissíveis, esta pesquisa possibilitou um mapeamento elencando fatores que contribuem para sua vulnerabilidade, contribuindo para visibilidade dessa população que necessita de atendimento de saúde, com vistas a minimização de transmissão de doenças, bem como, tratamento e reestabelecimento. Além do cumprimento de

um dos pilares da promoção da saúde que consiste em promover o bem estar dos cidadãos, no caso, pessoas em situação de rua.

A enfermagem constitui um dos membros da equipe multiprofissional de consultório de rua e da atenção primária que assiste estas pessoas, então, os resultados desta pesquisa demonstram fatores relevantes e fáceis de serem aplicados nesta prática clínica, de prevenção de doenças e promoção da saúde. Ao incorporar estes dados na anamnese o enfermeiro poderá prever situações de potencial vulnerabilidade ao HIV, IST's e a tuberculose, direcionando para uma assistência rápida, eficaz e assertiva.

Destaca-se que esta tese é relevante no contexto social e de saúde pública, pois subsidiará condutas de profissionais que assistem pessoas em situação de rua, no sentido de incrementar ações de prevenção de doenças e detecção precoce de fatores que influenciam o adoecimento por HIV e tuberculose.

Ressalta-se que o estudo, terá um efeito em toda saúde pública, a partir do momento, que pode ajudar a identificar, prevenir e a tratar o HIV/aids e a tuberculose, diminuindo o contágio, o número de casos e assim o alastramento dessas doenças.

Apesar de ter respondido a todos os objetivos e da pesquisa ter sido realizada com uma amostra de difícil acesso, apresentou limitações, como: ser um estudo transversal, detectando as variáveis pontualmente, apresenta reduzido quantitativo de amostra, ter sido realizada em dois locais, adiamento de algumas coletas por motivo de violência interna.

Por fim, sugere-se a criação de mais casas de apoio, facilitar o acesso a assistência à saúde, grupos de apoio que promovam a capacitação das PSR no intuito de trazer fonte de renda e reinseri-los na sociedade. Distribuir preservativo e realizar educação sexual nos centros de apoio. Garantir a política de redução de danos e encaminhá-los para centros de dependentes químicos para reabilitação, sendo assim, torna-se importante garantir o número de vagas suficientes. A fiscalização dos currículos dos cursos da área de saúde, de forma que estes abordem temáticas relacionadas as pessoas em situação de rua.

REFERÊNCIAS

ALAGNA, E. *et al*. Health status, diseases and vaccinations of the homeless in the city of Palermo, Italy. Ann Ig. v. 31,n.1,p. 21-34, 2019. Disponível em: doi:10.7416/ai.2019.2255. Acesso em: 6 jun. 2019.

ALDRIDGE, R.W. *et al*. Morbidity and motality individuals, prisoners, sex workers, and individuals with substance use disordes in high-income countries: a systematic review and meta-analysis. The Lancet. v.391,n.10117,p. 241-50,2018.

ALECRIM, T. F. A. *et al*. Experience of health professionals in care of the homeless population with tuberculosis. Rev Esc Enferm USP, v. 50, n. 5, p. 808-15, 2016. Disponível em: < http://dx.doi.org/10.1590/S0080-623420160000600014> Acesso em: 6 jun. 2019.

ALERE - Instruções de uso de testes rápidos para sífilis, 2017.

ALERE. Manual de uso do kit Alere HCV,2016.

ALESSANDRINI, G. *et al*. Treatment of alcohol dependence. Alcohol and homelessness: social point of view. Riv Psichiatr. v.53,n.3,p.107-12,2018. Disponível em: DOI 10.1708/2925.29411. Acesso em: 6 jun. 2019.

ALLAHQOLI, L. *et al*. A percepção das mulheres sexualmente transmissíveis sobre as infecções sexualmente transmissíveis: um estudo qualitativo. Life Sci J.v.11,n.4s,p.244-50, 2014.

AMIRI, F.B. *et al*. Knowledge. Atitude, and practices regarding HIV and TB among homeless people in Tehran, Iran. Int J Health policy Manag.v.7,n.6,p.549-55, 2018. Disponível em: https://www.ncbi.nlm.nih.gov/pmc/articles/PMC6015517/pdf/ijhpm-7-549.pdf Acesso em: 6 jun. 2019.

AMIRI, F.B. *et al*. Vulnerabilidade de moradores de rua em Teerã, no Irão, ao HIV, tuberculose e hepatite viral. PLoS One. v.9,n.6,p.e98742, 2014. Disponível em: doi: 10.1371 / journal.pone.0098742. Acesso em: 6 jun. 2019.

AMIRI, FB.; SEDAGHAT, A.; MOSTAFAVI, E. Diferenças de gênero entre pessoas em situação de rua em Teerã, Irão. J Saúde Pública. v. 22,n.5,p.461-5, 2014. Disponível em: doi: 10.1007 / s10389-014-0633-8. Acesso em: 8 jun. 2019.

ANJOS, A. Teoria da Resposta ao Item com uso do R. João Pessoa, PB 30 de julho a 3 de agosto de 2012.

ANTUNES, C.M.C.; ROSA, A.S.; BRÊTAS, A.C.P. From the stigmatizing disease to resignification of living on the streets. Rev Eletrôn Enferm.v.18,n.e1150,p. 2016. Disponível em: http://fi-admin.bvsalud.org/document/view/yx9jn Acesso em: 8 jun. 2019.

APTEKAR, L.;STOECKLIN, D. Children in street situations: Street children and homeless youth. In L. Aptekar & D. Stoecklin (Eds.), Street children and homeless youth: A crosscultural perspective. New York: Springer,2014.

ARAUJO, E.A.P.C; ANDRADE, D.F; BORTOLOTTI, S.L.V. Teoria da Resposta ao Item. Rev Esc Enferm USP.v.43,n.Esp,p.1000-8, 2009.

ARGINTARU, N. *et al*. Um estudo de observação transversal de necessidades de saúde não atendidas entre adultos sem abrigo e vulneráveis em três cidades canadenses. BMC Saúde Pública, v.13,n.1,p.577, 2013.

AXON R.N. *et al*. Differential Impact of Homelessness on Glycemic Control in Veterans with Type 2 Diabetes Mellitus. J Gen Intern Med. v.31,n.11,p.1331-7, 2016. Disponível em: doi: 10.1007/s11606-016-3786-z. Acesso em: 10 ago. 2019.

AYRES, J.R.; PAIVA, V.; FRANÇA JUNIOR, I. Conceitos e práticas de prevenção: da história natural da doença ao quadro da vulnerabilidade e direitos humanos. In: Paiva V,

AYRES, J.R.; BUCHALLA, C.M. Vulnerabilidade e direitos humanos - prevenção e promoção da saúde: da doença à cidadania. Curitiba (PR): Juruá, p.71-94, 2012.

AYRES, J.R.C.M. O conceito de vulnerabilidade e as práticas de saúde: novas perspectivas e desafios. Em: Czeresnia D, Freitas CM, (Org.). Promoção da saúde: conceitos, reflexões, tendências. Rio de Janeiro: Fiocruz; 2009. p.121-43.

AYRES, J.R.C.M.; PAIVA, V.; FRANÇA, I. From natural history of disease to vulnerability: changing concepts and practices in contemporary public health. In: Parker R, Sommer M, organizadores. Routledge Handbook in Global Public Health. Abingdon: Taylor and Francis Books, p. 98-107, 2011.

BAGBY, R.M.; RYDER, A.G.; SCHULLER, D.R.; MARSHALL, M.B. The Hamilton Depression Rating Scale: Has the Gold Standard Become a Lead Weight? Am J Psychiatry. v.161,p.2163-77, 2004.

BAGGETT, T.P.; LIAUW, S.S.; HWANG, S.W. Cardiovascular Disease and Homelessness. J Am Coll Cardiol.v.71,n.22,p.2585-597,2004. Disponível em: https://doi.org/10.1016/j.jacc.2018.02.077. Acesso em: 9 jun. 2019.

BALIERO, E.O.; SOARES, C.P.D.; VIEIRA, E.A. Morador de rua: causas, entraves e serviços ofertados. Temporalis.v.17,n.34,2017. Disponível em: http://periodicos.ufes.br/temporalis/article/view/14606/pdf_1 Acesso em: 9 jun. 2019.

BARBOSA, K.K.S. Significados e experiências de pessoas em situação de rua acometidas por tuberculose. 2018. 121f.Tese. (Doutorado em Enfermagem). Programa de Pós Graduação em Enfermagem da Universidade Federal da Paraíba. 2018.

BARMAN-ADHIKARI, A. *et al*. Uma abordagem de capital social para identificar correlatos do apoio social percebido entre os jovens sem-teto. Fórum de Juventude Infantil.v.45,p.691-708, 2016.

BARROS, C.V.L. *et al*. Bio-behavioral survey of syphilis in homeless men in Central Brazil: a cross-sectional study. Cad. Saúde Pública.v,34, n.6,p. e00033317, 2018 . Disponível em: http://www.scielo.br/pdf/csp/v34n6/1678-4464-csp-34-06-e00033317.pdf Acesso em: 9 jun. 2019.

BECK, A.T. et al. An inventory for measuring depression. Archives of General Psychiatry.v.4,p. 561-71, 1961.

BEECH, B.M.; MYERS, L.; BEECH, D.J.; KERNICK, N.S. Human immunodeficiency syndrome and hepatitis B and C infections among homeless adolescents. *Semin Pediatr Infect Dis*. 2003;14(1):12-9

BEIJER, U.; WOLF, A.; FAZEL, S. Prevalence of tuberculosis, hepatites C vírus, and HIV in homeless people: a systematic review and meta-analysis. Lancet.v.12,n.11,p. 859-70, 2012. Disponível em: https://ac.els-cdn.com/S1473309912701779/1-s2.0-S1473309912701779-main.pdf?_tid=d9d85685-9ff7-4aaa-8866-39b64a4cba89&acdnat=1548271093_08883db39ac835df2f95e5613a295276 Acesso em: 9 jun. 2019.

BEKELE, T. *et al*. Childhood adversities and physical and mental health outcomes in adults living with HIV: findings from the Ontario HIV Treatment Network cohort study. AIDS Research and Treatment. 2018.

BIOMÉRIEUX. Manual de uso do kit Vikia HBsAg,2016.

BISCOTTO, P.R. *et al*. Understanding of the life experience of homeless women. Rev Esc Enferm USP.v.50,n.5,p.750-6, 2016. Disponível em: DOI: http://dx.doi.org/10.1590/S0080-623420160000600006 Acesso em: 9 jun. 2019.

BONUGLI, R.; MENOR, J.; ESCANDON, S. A segunda coisa para o inferno é viver sob essa ponte: narrativas de mulheres que vivem com vitimização, doença mental grave e desamparo. Questões Ment Health Nurs.v.34,n.11,p. 827-35,2013.

BORLOTTI, S.L.V. *et al*. Proposta de avaliação da satisfação por meio do modelo logistico de dois parâmetros da teoria da resposta ao item. VI Congresso nacional de excelência em gestão Energia, Inovação, Tecnologia e Complexidade para a Gestão Sustentável Niterói, RJ, Brasil, 5, 6 e 7 de agosto de 2010.

BORYSOW, I.C.; CONILL, E.M.; FURTADO, J.P. Health care of people in homelessness: a comparative study of mobile units in Portugal, United States and Brazil. Ciência & Saúde Coletiva.v.22,n.3,p.879-90,2017. Disponível em: http://dx.doi.org/10.1590/1413-81232017223.25822016 Acesso em: 15 jun. 2019.

BOSKA, G. A. *et al*. Vulnerability to sexual risk behavior in users of alcohol and other drugs. Revista Eletrônica Saúde Mental Álcool Drog. v. 13, n. 04, p. 189-95, 2017. Disponível em: http://pepsic.bvsalud.org/pdf/smad/v13n4/en_03.pdf Acesso em: 17 de jan. 2019.

BOTTI, N.C.L. *et al*. Prevalência de depressão entre homens adultos em situação de rua em Belo Horizonte. J Bras Psiquiatri.v.59,n.1,p.10-16, 2010. Disponível em: http://www.scielo.br/pdf/jbpsiq/v59n1/v59n1a02 Acesso em: 7 set. 2019.

BRASIL. Ministério da Saúde, Secretaria de Vigilância em Saúde. Boletim Epidemiológico. Brasília.v.45,n.2,2014a.Disponível em: <http://portalsaude.saude.gov.br/images/pdf/2014/maio/29/BE-2014-45--2--tb.pdf>. Acesso em: 11 jun. 2019.

BRASIL. Ministério da Saúde. Populações vulneráveis. Disponível em: http://www.saude.gov.br/saude-de-a-z/tuberculose/populacoes-vulneraveis Acesso em: 20 set. 2019.

BRASIL. Ministério da Saúde. Portaria nº 2.436, de 21 de setembro de 2017. Aprova a Política Nacional de Atenção Básica, estabelecendo a revisão de diretrizes para a organização da Atenção Básica, no âmbito do Sistema Único de Saúde (SUS).

BRASIL. Ministério da Saúde. Secretaria de Ciência, Tecnologia e Insumos Estratégicos. Departamento de Ciência e Tecnologia. Síntese de Evidências para Políticas de Saúde : adesão ao tratamento de tuberculose pela população em situação de rua / Ministério da Saúde, Secretaria de Ciência, Tecnologia e Insumos Estratégicos, Departamento de Ciência e Tecnologia. – Brasília : Ministério da Saúde, 2016.

BRASIL. Ministério da Saúde. Secretaria de Vigilância em Saúde. Boletim Epidemiológico. Hepatites Virais 2018. Brasília, DF, v.49, n.31.

BRASIL. Ministério da Saúde. Secretaria de Vigilância em Saúde. Programa Nacional de DST e Aids. Diretrizes para implantação do Projeto Saúde e Prevenção nas Escolas. Brasília, Ministério da Saúde,2006.24p.

BRASIL. Secretaria de Vigilância em Saúde. Ministério da Saúde. Boletim Epidemiológico. 2015. Disponível em: http://portalarquivos.saude.gov.br/images/pdf/2015/marco/25/Boletim-tuberculose-2015.pdf

BRASILa. Decreto n.º 7.053, de 23 de Dezembro de 2009. Institui a Política Nacional para a População em Situação de Rua e seu Comitê Intersetorial de Acompanhamento e Monitoramento, e dá outras providências. Diário Oficial da União 24 Dez. 2009. Disponível em: http://www.planalto.gov.br/ccivil_03/_ato2007-2010/2009/decreto/d7053.htm Acesso em: 10 marc. 2019.

BRASILa. Hepatites Virais 2019. Boletim Epidemiológico. Secretaria de Vigilância em Saúde. Ministério da Saúde. v.50,n.17. Brasília/DF, 2019. Disponível em: file:///D:/Arquivos%20do%20Usuario%20N%C3%83O%20APAGAR/Documents/Downloads/boletim_hepatites_2019_c_.pdf

BRASILa. Ministério da Saúde (MS). Secretaria de Vigilância, Prevenção e Controle das Infecções Sexualmente Transmissíveis, do HIV/Aids e das Hepatites Virais. Boletim Epidemiológico HIV-AIDS, 2017.

BRASILa. Ministério da Saúde. Portaria nº 122, de 25 de janeiro de 2011. Define as diretrizes de organização e funcionamento das Equipes de Consultório na Rua.

BRASILa. Ministério da Saúde. Secretaria de Atenção à Saúde. Departamento de Atenção Básica. Manual sobre o cuidado à saúde junto a população em situação de rua / Ministério da Saúde. Secretaria de Atenção à Saúde. Departamento de Atenção Básica. – Brasília: Ministério da Saúde, 2012.

BRASILa. Ministério da Saúde. Secretaria de Vigilância em Saúde. Departamento de DST, Aids e Hepatites Virais. Manual Técnico para o Diagnóstico da Infecção Pelo HIV, 2013.

BRASILb. Ministério da Saúde. Secretaria de vigilância em saúde. Boletim Epidemiológico Sífilis 2018. v.49, 2018.

BRASILb. Ministério da Saúde (BR). Resolução nº 466, de 12 de dezembro de 2012. Dispõe sobre Pesquisas com Seres Humanos.Diário Oficial da União. Brasília, 2012. Disponível em: <http://conselho.saude.gov.br/resolucoes/2012/Reso466.pdf>.

BRASILb. Ministério da Saúde. Hepatite: causas, sintomas, diagnóstico, prevenção e tratamento, 2019. Disponível em: http://saude.gov.br/saude-de-a-z/hepatite

BRASILb. Ministério da Saúde. Resolução nº 2, de 27 de fevereiro de 2013. Define diretrizes e estratégias de orientação para o processo de enfrentamento das iniquidades e desigualdades em saúde com foco na População em Situação de Rua (PSR) no âmbito do Sistema Único de Saúde (SUS).

BRASILb. Ministério da Saúde. Saúde da população em situação de rua: um direito humano. Brasília: Ministério da Saúde, 2014.

BRASILb. Ministério da Saúde. Secretaria de Vigilância em Saúde. Boletim Epidemiológico HIV/aids, 2017. Disponível em: file:///D:/Arquivos%20do%20Usuario%20N%C3%83O%20APAGAR/Documents/Downloads/boletim_aids_internet%20(1).pdf

BRASILb. Resolução 109 de 11 de novembro de 2009. Aprova a Tipificação Nacional de Serviços Socioassistenciais. Diário Oficial da União, Seção 1, número 255, 25 nov. 2009. Disponível em: file:///D:/Arquivos%20do%20Usuario%20N%C3%83O%20APAGAR/Documents/Downloads/Legisla%C3%A7%C3%A3o_Resolu%C3%A7%C3%A3o%20CNAS%20109.2009.pdf Acesso em: 20 set. 2019.

BRASILb. Secretaria Nacional de Renda e Cidadania e Secretaria Nacional de Assistência Social Ministério do Desenvolvimento Social e Combate à Fome – MDS. Orientações Técnicas: Centro de Referência Especializado para População em Situação de Rua – Centro Pop. SUAS e População em Situação de Rua. v.3, Brasília, 2011.

BRASILc. Ministério da Saúde. Portaria nº 940, de 28 de abril de 2011. Regulamenta o Sistema Cartão Nacional de Saúde (Sistema Cartão).

BRASILc. Ministério da Saúde. Secretaria de Vigilância em Saúde. Boletim Epidemiológico Sífilis. 2017. Disponível em: file:///C:/Users/Anna%20Cl%C3%A1udia/Downloads/boletim_sifilis_11_2017.pdf Acesso em: 19 de ago. 2019.

BRASILc. Ministério da Saúde. Secretaria de Vigilância em Saúde. Boletim epidemiológico Tuberculose. v.49,2018.

BRITO, V.O.C.; PARRA, D.; FACCHINI R.; BUCHALLA, C.M. HIV infection, hepatitis B and C and syphilis in homeless people, in the city of São Paulo, Brazil. Rev. Saúde Pública vol.41 suppl.2 São Paulo Dec. 2007. Available from: http://www.scielo.br/pdf/rsp/v41s2/en_5951.pdf

CAMPOS, L.C.M. *et al*. Social representations held by homeless individuals regarding homeless individuals who consume drugs. Rev. baiana enferm. vol.33, n.e26778, p.1-9, 2019. Disponível em: http://www.revenf.bvs.br/pdf/rbaen/v33/en_1984-0446-rbaen-33-e26778.pdf

CAMPOS, L. S.; CUNHA, L. S. P. A busca pelo sentido da vida em meio a exclusão: um estudo logoterápico com pessoas em situação de rua. Revista Logos & Existência. v. 5, n. 2, p. 175-190, 2016. Disponível em: <http://www.periodicos.ufpb.br/ojs/index.php/le/article/view/28841/16845> Acesso em: 19 de ago. 2019.

CAO, K. *et al*. Spatial-Temporal Epidemiology of Tuberculosis in Mainland China: An Analysis Based on Bayesian Theory. Int J Environ Res Public Health.v.13,n.5,p.469, 2016. Disponível em: https://www.ncbi.nlm.nih.gov/pmc/articles/PMC4881094/pdf/ijerph-13-00469.pdf Acesso em: 19 jun. 2019.

CARRAPATO, P.; CORREIRA, P.; GARCIA, B. Health Determinants in Brasil: searching for health equity. Articles Saude soc. v.26,n.3,2017. Disponível em: https://doi.org/10.1590/S0104-12902017170304 Acesso em: 19 jun. 2019.

CARVALHO, P.M.R.S. *et al*. Prevalence, risk factors and hepatites B immunization: helping fill the gap on hepatites B epidemiology among homeless people, Goiânia, Central Brazil. Cad Saúde Pública. 2017. v.33,n.7,p.1-9. Disponível em: http://www.scielo.br/pdf/csp/v33n7/1678-4464-csp-33-07-e00109216.pdf Acesso em: 19 jun. 2019.

CASTRO, R.A.S. et al. Vulnerabilities of the homeless population to suicide behavior. Rev enferm UFPE on line, v.13,n.2,p.431-7, 2019. Disponível em: https://periodicos.ufpe.br/revistas/revistaenfermagem/article/view/237023/31353 Acesso em: 5 dez. 2019.

CENTRO EUROPEU DE PREVENÇÃO E CONTROLE DE DOENÇAS (ECDC) / Organização Mundial de Saúde (OMS).Escritório Regional para a Europa. Vigilância e monitoramento da tuberculose na Europa 2016.ECDC , Estocolmo, 2016 .

CHENG, J.M. *et al*. A Clonal outbreak of tuberculosis in homeless population the interior of British Columbia, Canada, 2008-2015. Epidemol Infect.v.143,n.15,p.:3220-6, 2015. Disponível em: DOI: https://doi.org/10.1017/S0950268815000825 Acesso em: 5 set. 2019.

CHENG, T. *et al*. Substance use patterns and unprotected sex among street-involved youth in a Canadian setting: a prospective cohort study. BMC Public Health.v.16,n.4,p.1-7, 2016. Disponível em: https://www.ncbi.nlm.nih.gov/pmc/articles/PMC4700772/#__ffn_sectitle Acesso em: 19 jun. 2019.

CONFERÊNCIA DE IMPRESSA DA ONU. 2018.

COOK, J.A. *et al*. Prevalence, comorbidity, and correlates of psychiatric and substance use disorders and associations with HIV risk behaviors in a multisite cohort of women living with HIV. AIDS Behav. 2018.

COSTA, M.I.F. Adolescentes em situação de pobreza: resiliência e vulnerabilidade às IST/HIV/aids. 2017.111f. Dissertação (Mestrado em Enfermagem)-Programa de Pós-Gradução em Enfermagem da Universidade Federal do Ceará. Fortaleza, 2017.

COSTA, S. L. *et al*. Homeless pregnant women in the city of Santos, São Paulo, Brazil: reflections and challenges to public policies. Saúde soc, v. 24, n.3, 2015. Disponível em: < https://doi.org/10.1590/S0104-12902015134769> Acesso em: 19 jun. 2019.

CRUZ, E.J.E.R. *et al*. Resilience as an object of study of occupational health:narrative review. Rev Cuid é fundamental online,v.10,n.1,p.283-8, 2018. Disponível em: https://docs.google.com/viewerng/viewer?url=http://www.seer.unirio.br/index.php/cuidadofundamental/article/viewFile/5047/pdf Acesso em: 19 jun. 2019.

CUNHA, J. A. Manual da versão em português das Escalas Beck. São Paulo: Casa do Psicólogo Livraria e Editora, 2001.

DASGUPTA, S. *et al*. Injection Practices and Sexual Behaviors Among Persons with Diagnosed HIV Infection Who Inject Drugs — United States, 2015–2017. v.68,n.30,p.653–7, 2019. Disponível em: https://www.cdc.gov/mmwr/volumes/68/wr/mm6830a1.htm?s_cid=mm6830a1_w Acesso em: 19 jun. 2019.

DEGENHARDT, L. *et al*. Global prevalence of injecting drug use and sociodemographic characteristics and prevalence of HIV, HBV, and HCV in people who inject drugs: a multistage systematic review. The Lancet Global Health.v.5,n.12,p.PE1192-E1207,2017. Available from: https://www.thelancet.com/journals/langlo/article/PIIS2214-109X(17)30375-3/fulltext#seccestitle10 Acesso em: 19 jun. 2019.

DELL, C.A. *et al*. Volte ao básico: identificando o desenvolvimento positivo da juventude como um quadro teórico para um programa de prevenção de drogas para jovens na Saskatchewan rural, no Canadá, em meio a uma avaliação do programa. Subst Abuse Treat Prev Policy,v.8,n.1,p. 36, 2013.

DESMENNU, A.T.; TITILOYE, M.A.; OWOAJE, E.T. Behavioural risk factors for sexually transmitted infections and health seeking behaviour of street youths in Ibadan, Nigeria. Afr Health Sci.v.18,n.1,p.180-7,2018. Disponível em: doi: 10.4314/ahs.v18i1.23. Acesso em: 19 jun. 2019.

DIAZ, A. *et al*. Gonorrhoea diagnoses in a network of STI clinics in Spain during the period 2006–2010: differences by sex and transmission route. BMC Public Health.v.13,p.1093, 2013. Disponível em: DOI: 10.1186/1471-2458-13-1093 Acesso em: 5 set. 2019.

DUKO, B. *et al*. Depression, Anxiety and Their Correlates Among Patients With HIV in South Ethiopia: An Institution-Based Cross-Sectional Study. Front Psychiatry. v.7,n.10,p.290. Disponível em: doi: 10.3389/fpsyt.2019.00290. Acesso em: 25 jun. 2019.

DUNNE, E.; DUGGAN, M.; O'MAHONY, J. Serviço de saúde mental para pessoas sem-teto: perfil do paciente e fatores associados ao suicídio e ao homicídio . IR Med J. v.105,p.71-2, 2012.

ERIKSEN, M.P. The Tobacco Atlas. 5ª ed. The American Cancer Society, 2015: 46.

EUSEBI, P. Diagnostic accuracy measures. Cerebrovasc Dis. v.36,n.4,p.267-72,2013.

FAGBAMIGBE, A.F.; LAWAL, A.M.; IDEMUDIA, E.S. Modelling self-assessed vulnerability to HIV and its associated factors in a HIV-burdened country. J SAHARA, v.14,n.1,p.140-52, 2017. Disponível em: https://www.ncbi.nlm.nih.gov/pmc/articles/PMC5678227/pdf/rsah-14-1387598.pdf Acesso em: 25 jun. 2019.

FARQUHAR , J.; KAMEI, R.; VIDARTHI, A. Estratégias para melhorar a resiliência dos estudantes de medicina: perspectivas dos alunos e do corpo docente. Int J Med Educ, v.9,n.1,p.1-6, 2018. Disponível em: https://www.ijme.net/archive/9/strategies-to-enhance-medical-student-resilience/?ref=linkout Acesso em: 25 jun. 2019.

FAZEL, S.; GEDDES, J.R.; KUSHEL, M. A saúde dos moradores de rua em países de alta renda: epidemiologia descritiva, consequências para a saúde e recomendações clínicas e políticas. Lancet.v.384,p. 1529-40,2014. https://doi.org/10.1016/s0140-6736(14)61132-6.

FERNANDEZ, D.B. *et al*. HIV vulnerability index in homeless persons. Enfermeria Global. v.16,n.46,p.154-81,2017. Disponível em: http://scielo.isciii.es/pdf/eg/v16n46/en_1695-6141-eg-16-46-00154.pdf Acesso em: 25 jun. 2019.

FIGUEROA-MUNOZ, J.; RAMON-PARDO, P. Tuberculosis controle em grupos vulneráveis. Organização Mundial da Saúde, Genebra, 2008.

FIORATI, C. R. *et al*. Inequity and social exclusion: a study with homeless population in Ribeirão Preto/SP. Revista eletrônica gestão & saúde, v. 6, n. 3, p. 2120-2135, 2015. Disponível em:<http://periodicos.unb.br/ojs311/index.php/rgs/article/view/3083. Acesso em 31 de jan 2019.

FIORATI, R.C. *et al*. Social ruptures and the everyday life of homeless people: an ethnographic study. Revista Gaúcha Enfermagem. v.37,2016. Disponível em: < http://www.scielo.br/pdf/rgenf/v37nspe/0102-6933-rgenf-1983-14472016esp72861.pdf>. Acesso em: 04 jul. 2019.

FRANCO, J.F. *et al*. Relação entre a ocorrência de tuberculose e um conjunto de fatores sócioeconômicos, demográficos e de saúde da população brasileira usando a PNAD. 2003 Disponível em: http://www.ime.unicamp.br/sinape/sites/default/files/Trabalho_19Sinape.PDF Acesso em: 10 maio. 2019.

FREIRE, M.A. *et al*. Escala Hamilton: estudo das características psicométricas em uma amostra do sul do Brasil. J Bras Psiquiatr.v.63,n.4,p.281-9,2014. Disponível em: http://www.scielo.br/pdf/jbpsiq/v63n4/0047-2085-jbpsiq-63-4-0281.pdf Acesso em: 04 jul. 2019.

GALLUCCI NETO, J.; CAMPOS JÚNIOR, M.S.; HÜBNER, C.K. Escala de Depressão de Hamilton (HAM-D): revisão dos 40 anos de sua utilização. Rev Fac Ciênc Méd, v.3,n.1,p.10-4, 2001.

GARCIA, R.; RAMOS, D.G. Experiências de homens que fazem sexo com homens vivendo com hiv: diferentes processos emocionais. CuidArte, Enferm. v.11,n.1,p.93-9,2017. Disponível em: http://www.webfipa.net/facfipa/ner/sumarios/cuidarte/2017v1/13%20ARTIGO%20EXPERI%C3%8ANCIAS%20DE%20HOMENS%20QUE%20FAZEM%20SEXO%20COM%20HOMENS%20VIVENDO%20COM%20HIV.pdf Acesso em: 10 maio. 2019.

GEDDS J.R.; FAZEL S. Extreme Health inequalities: mortality in homeless people. Lancet.v.377,n.9784,p.2156-7,2011. Disponível em: https://ac.els-cdn.com/S0140673611608854/1-s2.0-S0140673611608854-main.pdf?_tid=9b2bfa07-5219-4d9d-bb21-3b671911e127&acdnat=1548269782_10fe4f9efcfd9de64074ac30670216f6 Acesso em: 04 jul. 2019.

GOETSCH, U. *et al*. Tuberculose entre usuários de drogas e pessoas em situação de rua: impacto da investigação radiográfica voluntária na busca ativa de casos. Infecção.v.40,n.4,p. 389-95,2012.

GOLDEN, M.R. *et al*. Outbreak of Human Immunodeficiency Virus Infection Among Heterosexual Persons Who Are Living Homeless and Inject Drugs - Seattle, Washington, 2018. MMWR Morb Mortal Wkly Rep. v.68,n.15,p.344-9,2019. Disponível em: DOI: http://dx.doi.org/10.15585/mmwr.mm6815a2 Acesso em: 10 maio. 2019.

GONÇALVES, D.M.; STEIN, A.T.; KAPCZINSKI, F. Avaliação de desempenho do SelfReporting Questionnaire como instrumento de rastreamento psiquiátrico: um estudo comparativo com o Structured Clinical Interview for DSMIV-TR. Cad Saúde Pública, v.24,n.2,p.380-90, 2008. http://dx.doi.org/10.1590/ S0102-311X2008000200017. Acesso em: 04 jul. 2019.

GORENSTEIN, C.; ANDRADE, L. Validação de uma versão em português do Inventário de Depressão de Beck e do Inventário de Ansiedade Traço-Estado em sujeitos brasileiros. Braz J Med Biol Res. v.29,p.453-7, 1996.

GORENSTEIN, C.;ANDRADE, L. Inventário de Depressão de Beck: propriedades psicométricas da versão em português. Revista de Psiquiatria Clínica. v.25,n.5,p.245-50,1998.

GUILLEMIN, F.; BOMBARDIER, C.; BEATON, D. Cross-cultural adaptation of health-related quality of life measures: literature review and proposed guidelines. J Clin Epidemiol.v.46,n.12,p.1417-32,1993.

HAGENAARS, J.A.; MCCUTCHEON, A. Applied latent class analysis. New York: Cambridge University Press, 2002.

HAIR, J. et al. Análise multivariada de dados. Porto Alegre: Bookman, 2005.

HALPERN, S.C. *et al*. Vulnerabilidades clínicas e sociais em usuários de crack de acordo com a situação de moradia: um estudo multicêntrico de seis capitais brasileiras. Cad Saúde Pública, v.33,n.6,p.1-13, 2017. Disponível em: http://www.scielo.br/pdf/csp/v33n6/1678-4464-csp-33-06-e00037517.pdf Acesso em: 04 jul. 2019.

HEUVELINGS, C.C. *et al*. Effectiveness of interventions for diagnosis and treatment of tuberculosis in hard-to-reach populations in countries of low and medium tuberculosis incidence: a systematic review. Lancet Infect Dis.v.17,n.5,p.e144-e158,2017. Disponível em: DOI: https://doi.org/10.1016/S1473-3099(16)30532-1 Acesso em: 05 ago. 2019.

HILL, L.M. *et al*. Peer norms moderate the association between mental health and sexual risk behaviors among young men living in Dar Es Salaam, Tanzania. Soc Sci Med.v.196,p.77–85, 2018.

HILLS, F; WEITZ, A. M; ASANTE, K. O. The lived experiences of street children in Durban, South Africa: violence, substance use, and resilience. Int J Qualitative Stud Health Well-being.v.11, n.1, p.1-11, 2016.Disponível em:<https://www.ncbi.nlm.nih.gov/pmc/articles/PMC4904070/pdf/QHW-11-30302.pdf> Acesso em: 13 de Ago. 2019.

HOCHMAN, B.; NAHAS, F.X.; OLIVEIRA FILHO, R.S.; FERREIRA, L.M. Desenhos de pesquisa. Acta Cirúrgica Brasileira. v. 20 ,n.(Supl. 2), 2005. Disponível em: http://www.scielo.br/pdf/acb/v20s2/v20s2a02.pdf Acesso em: 27 ago. 2019.

HUGHES, J.R. et al. Youth Homelessness: The Relationships among Mental Health, Hope, and Service Satisfaction. J Can Acad Child Adolesc Psychiatry.v.19,n.4,p.274–83, 2010. Disponível em: https://www.ncbi.nlm.nih.gov/pmc/articles/PMC2962540/pdf/cacap19_4p274.pdf Acesso em: 27 ago. 2019.

HUSAIN, M.O. *et al*. a relação entre ansiedade, depressão e percepção da doença em pacientes com tuberculose no Paquistão. Clin Pract Epidemiol Saúde Mental.v.4,n.14,p.15, 2008.

HWANG, S.W.; QUIÑONEZ, C. Saúde bucal de adultos sem teto em Toronto, Canadá. J. Public Health Dent.v.73,p.74–8,2013.

INSTITUTO DE PESQUISA ECONÔMICA APLICADA. Estimativa da População em Situação de Rua no Brasil. Brasília, 2016.

IRWIN, J. *et al*. Ativos sociais e angústia mental entre os desabrigados: Explorando os papéis do apoio social e outras formas de capital social na depressão. Soc Sci Med. v.67,p.1935–1943, 2008. Disponível em: https://doi.org/10.1016/j.socscimed.2008.09.008 Acesso em: 7 set. 2019.

KENRICK, D.T. *et al*. Renovando a pirâmide de necessidades. Perspectivas da Ciência Psicológica. v.5,p.292-314,2010.

KENT, M.E.; ROMANELLI, F. Reexaminando Sífilis: uma atualização sobre epidemiologia, Manifestações Clínicas e Gerenciamento. Ann Pharmacother,v.42,n.2,p.226-36, 2008.

KHAN, K. *et al*. Tuberculose ativa entre pessoas em situação de rua, Toronto, Ontário, Canadá, 1998-2007 . Emerg Infect Dis.v.17,p. 357-365, 2011. Disponível em: 10.3201 / eid1703.100833. Acesso em: 05 ago. 2019.

KIMURA, M. *et al*. First case of sexually transmitted asymptomatic female genital tuberculosis from spousal epididymal tuberculosis diagnosed by active screening.

International Journal of Infectious Diseases.v.73,p.60-2, 2018. Disponível em: DOI: https://doi.org/10.1016/j.ijid.2018.05.021 Acesso em: 6 set. 2019.

KIRST, M. *et al*.The promise of recovery: narratives of hope among homeless individuals with mental illness participating in a Housing First randomised controlled trial in Toronto, Canada. BMJ Open. v.4,n.3,p. e004379, 2014 Disponível em: http://dx.doi.org/10.1136/bmjopen-2013-004379. Acesso em: 05 ago. 2019.

KLINKENBERG, W.D. *et al*. Prevalence of human immunodeficiency virus, hepatitis B, and hepatitis C among homeless persons with co-occurring severe mental illness and substance use disorders. Comprehensive psychiatry, v. 44, n. 4, p. 293-302, 2003.

LAPORTE, A. *et al*. Prevalence of Mental Disorders and addictions among Homeless people in the Greater Paris Area, France. Int. J. Environ. Res. Public Health.v.15,p.241,2018. Disponível em: doi:10.3390/ijerph15020241. Acesso em: 05 ago. 2019.

LAWDER, J.A.C. *et al*. Impact of oral condition on the quality of life of homeless people. Rev Saude Publica.v.53,p.22, 2019. Disponível em: DOI: https://doi.org/10.11606/S1518-8787.2019053000718 Acesso em: 05 ago. 2019.

LEBRUN-HARRIS, L.A. *et al*. Health status and health care experiences among homeless patients in federally supported health centers: findings from the 2009 patient survey. Health Serv Res. v.48,n.3,p.992-1017, 2013. Disponível em: doi: 10.1111/1475-6773.12009. Acesso em: 05 ago. 2019.

LUCHENSKI, S. *et al*. What Works in inclusion health: overview of effective interventions for marginalised and exclused populations. Lancet.v.391,n.10117,p.266-80, 2018. Disponível em: https://ac.els-cdn.com/S0140673617319591/1-s2.0-S0140673617319591-main.pdf?_tid=0ab8bc3a-ea4b-4f32-b57b-8350f4147d71&acdnat=1548268407_b5bb10ccef45ad8f872efa91b08ce6e5 Acesso em: 05 ago. 2019.

MABHALA, M.; YOHANNES, A.; GRIFFITH, M. Social conditions of becoming homelessness: qualitative analysis of life stories of homeless peoples. Int J Equity Health. v.16,p.150, 2017. Disponível em: https://www.ncbi.nlm.nih.gov/pmc/articles/PMC5568348/pdf/12939_2017_Article_646.pdf Acesso em: 05 ago. 2019.

MABHALA, M.A.; YOHANNES, A.; GRIFFTH, M. Social conditions of becoming homelessness: qualitative analysis of life stories of homeless peoples. Int J Equity Health. 2017; 16: 150. Disponível em: doi: 10.1186/s12939-017-0646-3 Acesso em: 05 ago. 2019.

MARIA, D.S. *et al*. Current age, age at first sex, age at first homelessness, and HIV risk perceptions predict sexual risk behaviors among sexually active homeless adults. Int J. Environ Res Public Health.v.15,n.2,p.1-12,2018. Disponível em: https://www.ncbi.nlm.nih.gov/pmc/articles/PMC5858287/pdf/ijerph-15-00218.pdf Acesso em: 05 ago. 2019.

MAROJELE, N.K.; PARRY, C.; KEKWALETSWE, C. Alcohol and drug use. In: Van Niekerk A, Suffla S, Seedat M, editors. Crime, Violence and Injury in South Africa: 21st century solutions for child safety. Tygerberg, Cape Town: Peace Promotion Research Unit,p.195-213, 2012.

MARSHALL, B.D.L. *et al*. Condom usam usuários de drogas injetáveis que acessam uma instalação de injeção supervisionada. Infecção por Transmissão Sexual v.85,n.2, p.121-6, 2009.

MASLOW, A.H. A Theory of Human Motivation. 1943. Disponível http://psychclassics.yorku.ca/Maslow/motivation.htm. Acesso em: 05 ago. 2019.

MATOS, M. A. Qualidade de vida de pessoas em situação de rua do brasil central.2016. Dissertação(Mestrado em Enfermagem) – Faculdade de Enfermagem da Universidade Federal de Goiás, GO. Disponível em: <https://repositorio.bc.ufg.br/tede/bitstream/tede/6714/5/Disserta%C3%A7%C3%A3o%20-%20Alcides%20Pereira%20Soares%20Neto%20-%202016.pdf> Acesso em: 10 de set. 2019.

MEDEIROS, R.P. Bêbados, noiados e moradores de rua. In: Fernandez OFRL, Andrade MM, Nery Filho A, organizadores. Drogas e políticas públicas: educação, saúde coletiva e direitos humanos. Salvador: EDUFBA/Brasília: ABRAMD. p. 19-40, 2015.

METZ, C. E. "Statistical Analysis of ROC Data in Evaluating Diagnostic Performance." Multiple Regression Analysis: Applications in the Health Sciences, number 13, edited by Donald E. Herbert and Raymond H. Myers. 365–384. American Institute of Physics, 1986.

MIMIAGA, M.J. *et al*. The effect of psychosocial syndemic production on 4-year HIV incidence and risk behavior in a large cohort of sexually active men who have sex with men. J Acquir Immune Defic Syndr.v.68,n.3,p.329–36,2015.

MOORE, D.S. A estatística básica e sua prática. Rio de Janeiro:LTC, 2000.

MORENO, R.A.; MORENO, D.H. Escalas de depressão de Montgomery & Asberg (MADRS) e de Hamilton (HAM-D). Rev Psiquiatr Clin.v.25,p.262-72, 1998.

MORERA, J. A. C.; PADILHA, M. I. A dinâmica das relações familiares de moradores de rua usuários de crack. Saúde debate. Rio de Janeiro, v.39, n.106, 2015. Disponível em: <https://doi.org/10.1590/0103-1104201510600030015> Acesso em: 15 de ago. 2019.

MTHEMBU, J.C. *et al.* Prevalence of psychological distress and its association with socio-demographic and HIV-risk factors in South Africa: findings of the 2012 HIV prevalence, incidence and behaviour survey. SSM - Population Health.v.3,p.658–62, 2017.

MTHEMBU, S.; NDATEBA, I. Exploração de conhecimentos, atitudes e comportamentos de crianças de rua na prevenção do HIV e SIDA no distrito de Huye, Ruanda . East Afr J Public Health. v.9,n.1,p.74-79, 2012.

MUDINGAYI, A.; LUTALA, P.; MUPENDA, B. Conhecimento do HIV e comportamento de risco sexual entre adolescentes de rua em centros de reabilitação em Kinshasa; DRC diferenças de gênero . PanAfricana Med J. v.10,n.23,p.1017, 2011. Disponível em: https://www.ncbi.nlm.nih.gov/pmc/articles/PMC3224059/?report=reader Acesso em: 10 ago. 2019.

NANZALUKA, F.H. *et al.* Factors associated with unfavourable tuberculosis treatment outcomes in Lusaka, Zambia, 2015: a secondary analysis of routine surveillance data. Pan Afr Med J.v.32,p.159, 2019. Disponível em:

https://www.ncbi.nlm.nih.gov/pmc/articles/PMC6609856/pdf/PAMJ-32-159.pdf Acesso em: 10 ago. 2019.

NASCIMENTO, V. S. “Eu não quero ser pequeno nem grande, quero ser igual”. Significações sobre o futuro construídas por pessoas em situação de rua. 2018. Dissertação (Mestrado em Psicologia) – Universidade Federal da Bahia, BA. Disponível em:<https://repositorio.ufba.br/ri/bitstream/ri/25936/1/DISSERTA%c3%87%c3%83O.pdf> Acesso em: 22 de Ago. 2019.

NIKOVSKA, D.C.; TOZIJA, F. Social determinants of equity in access to healthcare for tuberculosis patients in Republic of Macedonia – results from a casecontrol study. Int J Health Pollicy Manag. v.3,n.4,p.199–205, 2014. Disponível em: http://ijhpm.com/article_2891_50ef3a379f5c4bfd50bc99a1616a4d7d.pdf Acesso em: 10 ago. 2019.

OLIVEIRA, D.M. *et al.* Needs, expectations and care production of people in street situation. Rev Bras Enferm. v.71,n.supl 6, p. 2849-2857, 2018. Disponível em: http://www.scielo.br/pdf/reben/v71s6/0034-7167-reben-71-s6-2689.pdf Acesso em: 10 ago. 2019.

OPPONG, A.K. Crianças e adolescentes de rua em Gana: um estudo qualitativo de trajetória e experiências comportamentais de sem abrigo. Bem estar social global.v.3,n.1,p.33-43, 2016.

PAIVA, I.K.S. *et al*. Homeless people’s right to health: reflections on the problems and components. Ciência & Saúde Coletiva. v.21,n.8,p. 2595-606,2016.Disponível em: http://www.scielo.br/pdf/csc/v21n8/en_1413-8123-csc-21-08-2595.pdf Acesso em: 10 ago. 2019.

PANADERO, S.; VÁZQUEZ, J.J.; MARTÍN, R.M. Álcool, pobreza e exclusão social: consumo de álcool entre os sem abrigo e pessoas em risco de exclusão social em Madrid. Adicciones.v.29,p.33-6, 2017.

PARKER, R.D.; DYKEMA, S. Differences in Risk Behaviors, Care Utilization, and Comorbidities in Homeless Persons Based on HIV Status. J Assoc Nurses AIDS Care.v.25,n.3, p.214-23, 2014.

PASQUALI, L. Psicometria. Revista da Escola de Enfermagem da USP. v.43, n.SPE,p.992-9,2009.

PASQUALI, L.; PRIMI, R. Fundamentos da Teoria da Resposta ao Item – TRI. Avaliação Psicológica. v.2,n.2,2003.

PATRICIO, S.T.; AJURIA, A.F.; CASTRO, L.C. Características de Los ingressos hospitalarios de las personas sin hogar en Sevilla. Rev Esp Salud Pública.v.90,n.1,p.1-11, 2016. Disponível em: http://www.msssi.gob.es/biblioPublic/publicaciones/recursos_propios/resp/revista_cdrom/VOL90/ORIGINALES/RS90C_STP.pdf Acesso em: 10 ago. 2019.

PESCE, R.P. *et al*. Adaptação transcultural, confiabilidade e validade da escala de resiliência. Cadernos de Saúde Pública.v.21,n.2,p. 436-48, 2005.

PINTO, V.M. *et al*. Prevalence of Syphilis and associated factors in homeless people of Sao Paulo, Brazil, using a Rapid Test. Rev. bras. epidemiol.v.17,n.2,p.341-54, 2014. Disponível em: http://www.scielo.br/pdf/rbepid/v17n2/1415-790X-rbepid-17-02-00341.pdf Acesso em: 10 ago. 2019.

PORTO, C.C. Semiologia médica. Rio de Janeiro: Guanabara Koogan, 2012.

PRABHAKARAN, S. Performance Analysis and Companion Functions for Binary Classification Models. Version 1.2.3. Bangalore, 30 out. 2016. R Package ‘InformationValue’. Disponível em: http://r-statistics.co/Information-Value-With-R.html

PRUDÊNCIO, C.V. *et al*. Temperature and pH influence the susceptibility of Salmonella Typhimurium to nisin combined with EDTA. Food Control.v.61,p.248–53, 2016.

RAMIRO, M.T. *et al*. Social support, self-esteem and depression: relationship with risk for sexually transmitted infections/HIV transmission. Int J Clin Health Psychol.v.13,n.3,p.181–8, 2013.

RESENE, V. M. Violência simbólica: representação discursiva da extrema pobreza no Brasil- relações entre situação de rua e vizinhança. Discurso &Sociedad. v. 9, p. 106-28, 2015. Disponível em: <www.dissoc.org/ediciones/v09n01-2/DS9(1-2)Resende.pdf> Acesso em: 10 de Set. 2019.

RHOADES, H. *et al*. Predisposing, enabling and need correlates of mental health treatment utilization among homeless men. Community Ment Health J. v. 50,n.8,p.943-52, 2014. Disponível em: https://www.ncbi.nlm.nih.gov/pmc/articles/PMC4864028/pdf/nihms783045.pdf

RHOADES, H.; WINETROBE, H.; RICE, E. Pet Ownership Homeless Youth: associations with mental health service utilization and housing status. Child Psychiatry Hum Dev. 2015. v.46,n.2, p. 237-244. Disponível em: https://www.ncbi.nlm.nih.gov/pmc/articles/PMC4194276/pdf/nihms-585606.pdf Acesso em: 10 ago. 2019.

ROOK, M. I. Aspectos conceituais e metodológicos da resiliência psicológica: uma análise da produção cientifica brasileira. Estudos e Pesquisas em Psicologia.v. 15, n. 2, p. 671 – 687, Rio de Janeiro, 2015. Disponível em: <http://www.redalyc.org/pdf/4518/451844504013.pdf> Acesso em: 12 de Ago. 2019.

ROY, É. Et al. Predictors of residential stability among homeless young adults: a cohort study. BMC Public Health,v.16,p.131, 2016.

RUI, T. Nas tramas do crack: etnografia da abjeção. São Paulo: Terceiro Nome, 2014.

RUIZ-BARQUÍN, R.L.V.; DE LA ROCHA, M.; BATISTA, F. Resiliência em adaptadores de remo adaptados. Actas do Congresso Nacional de Investigação em Pádel Adaptado. Granada (España), 2015.

SANTOS, A.C.C.; SOARES, D.J.; RIVEMALES, M.C.C. (Des)conhecimento, adoecimento e limitações impostas pelo HTLV: experiências de mulheres soropositivas. Cad Saúde Colet.v.25,n.1,p.45-50, 2017. Disponível em: http://www.scielo.br/scielo.php?pid=S1414-462X2017000100045&script=sci_arttext Acesso em: 10 ago. 2019.

SANTOS, K.O.B. *et al*. Avaliação de um instrumento de mensuração de morbidade psíquica: Estudo de validação do Self-reporting questionnaire (SQR20). Revista Baiana de Saúde Pública. v.34,n.3,p.544-60, 2009. Disponível em: http://www.scielo.br/pdf/csp/v25n1/23.pdf Acesso em: 20 ago. 2019.

SAPERSTEIN, A.M. *et al*. Cognitive Deficit and Mental Health in Homeless Transition-Age Youth. Pediatrics.v.134,n.1,p.e138-e145,2014.Disponível em: https://www.ncbi.nlm.nih.gov/pmc/articles/PMC4067643/pdf/peds.2013-4302.pdf Acesso em: 20 ago. 2019.

SCHMIDT, R. *et al*. Trajetórias de sem teto das mulheres nos 3 territórios do Norte do Canadá. Int J Health,v.74,p.29778, 2015. Disponível em: https://www.ncbi.nlm.nih.gov/pmc/articles/PMC4696460/?tool=pubmed#__ffn_sectitle Acesso em: 20 ago. 2019.

SCHREITER, S. *et al*. The prevalence of mental illness in homeless people in Germany—a systematic review and meta-analysis. Dtsch Arztebl Int.v.114,p.665–72,2017. DOI: 10.3238/arztebl.2017.0665. Acesso em: 20 ago. 2019.

SIDDIQI, N. Credit Risk Scorecards: Developing and Implementing Intelligent Credit Scoring. 1. ed. New Jersey: Wiley. p.196, 2006. ISBN 978-0-471-75451-0.

SILVA, I.C.N. *et al*. Social representations of health care by homeless people. Rev Esc Enferm USP. v,52,p.e03314, 2018. Disponível em: DOI: http://dx.doi.org/10.1590/S1980-220X2017023703314. Acesso em: 20 ago. 2019.

SKEVINGTON, S.M.; SOVETKINA, C.E.; GILLISON, F.B. Uma revisão sistemática para avaliar quantitativamente 'Stepping Stones': uma intervenção comunitária participativa de prevenção do HIV / AIDS. AIDS Behav. v.17,n.3,p.1025-39, 2013. Disponível em: doi: 10.1007 / s10461-012-0327-6. Acesso em: 20 ago. 2019.

SOPHEAB, H. *et al*. HIV prevalence, related risk behaviors, and correlates of HIV infection among people who use drugs in Cambodia. BMC Infectious diseases.v.18,p.562, 2018. Disponível em: https://www.ncbi.nlm.nih.gov/pmc/articles/PMC6234604/pdf/12879_2018_Article_3472.pdf Acesso em: 25 ago. 2019.

SOUSA, A.G.L. Sou feio, pobre, sujo e alcoólico: Emoções e sociabilidade dos moradores das ruas de João Pessoa - PB. Revista Brasileira de Sociologia da Emoção, v. 8, n. 23, p. 373-416, 2009.

SSEWAMALA, F.M. *et al*. Suubi4Her: a study protocol to examine the impact and cost associated with a combination intervention to prevent HIV risk behavior and improve mental health functioning among adolescent girls in Uganda. BMC Public Health.v.18,n.1,p.693, 2018.

STENIUS-AYOADE, A. *et al*. Mental disorders and the use of primary health care services among homeless shelter users in the Helsinki metropolitan area, Finland. BMC Health Serv Res. v.17,p. 428, 2017. Disponível em: doi: 10.1186/s12913-017-2372-3. Acesso em: 25 ago. 2019.

STRAANTEN, V.B. *et al.* Intellectual Disability among Dutch Homeless People: prevalence and related psychosocial problems. Plos One. v.9,n.1,p.1-7, 2014. Disponível em: https://www.ncbi.nlm.nih.gov/pmc/articles/PMC3897643/pdf/pone.0086112.pdf Acesso em: 25 ago. 2019.

STREHLOW, A.J. *et al*. Hepatitis C among clients of health care for the homeless primary care clinics. Journal of health care for the poor and underserved. v. 23, n. 2, p. 811, 2012.

SZYMKOWIAK, D. *et al*. Super-utilização persistente de services de cuidados intensivos entre os subgrupos de veteranos que vivenciam sem-teto. Med Care. v.55,n.10,p.893-900, 2017.

TAO, J. *et al*. Efeitos da depressão e ansiedade na adesão à terapia antirretroviral entre HSH chineses infectados pelo HIV recém-diagnosticados . AIDS .v.31,n.3,p. 401 – 6,2017. Disponível em: doi: 10.1097/QAD.0000000000001287 Acesso em: 10 jul. 2019.

TIOZER, K. *et al*. Reorientando o risco para a resiliência: perspectivas da juventude envolvidas na rua sobre a prevenção da transição para o uso de drogas injetáveis. BMC Public Health.v.15,p.800,2015. Disponível em: https://www.ncbi.nlm.nih.gov/pmc/articles/PMC4545775/?tool=pubmed#__ffn_sectitle Acesso em: 25 ago. 2019.

TOLEDO, L.; GÓNGORA, A.; BASTOS, F.I.P.M. À margem: uso de crack, desvio, criminalização e exclusão social - uma revisão narrativa. Ciênc Saúde Coletiva.v.22,p.31-42, 2017.

TOMS, C. *et al*. Tuberculosis notificações na Austrália 2012 e 2013.Commun Dis Intell, v.39,n.1,p.217-235, 2015.

TOZER, K. *et al*. Reorienting risk to resilience: street-involved youth perspectives on preventing the transition to injection drug use. BMC Public Health.v.15,p.800,2015. Disponível em: https://www.ncbi.nlm.nih.gov/pmc/articles/PMC4545775/pdf/12889_2015_Article_2153.pdf Acesso em: 25 ago. 2019.

UNGAR, M. A Constructionist Discourse on Resilience: Multiple Contexts, Multiple Realities among At-Risk Children and Youth. Youth & Society.v.35,n.3,p.341–65, 2004. Disponível em: doi: 10.1177/0044118X03257030. Acesso em: 25 ago. 2019.

UNGAR, M. A ecologia social da resiliência: abordando a ambiguidade contextual e cultural de uma construção nascente. American Journal of Orthopsychiatry. v.81,n.1,p.1-17, 2011.

UNGAR, M. *et al*. Distinguishing differences in pathways to resilience among Canadian youth. Can J Commun Ment Health.v.27,n.1,p.1–13,2008. Disponível em: doi: 10.7870/cjcmh-2008-0001. Acesso em: 25 ago. 2019.

UNGAR, M. Resiliência em todas as culturas. British Journal of Social Work, v.38,n.1,p.218-235, 2008.

UNGAR, M. Resiliência, trauma, contexto e cultura . Trauma, Violência e Abuso. v.14,p. 255-266, 2013. Disponível em: DOI: 10.1177 / 1524838013487805. Acesso em: 25 ago. 2019.

URADA, L.A. *et al*. Sex Trade and Health Care Utilization Among People Living with HIV/AIDS. AIDS Behav. v.22,n.8,p.2553-63,2018. Disponível em: doi: 10.1007/s10461-018-2131-4. Acesso em: 25 ago. 2019.

USAID. Conhecimento de HIV / AIDS Outras DSTs e Formas de Prevenção, 2010.

VERULAVA, T. *et al*. Kowledge of Sexually Transmitted Infections Among Homeless People in a Municipal Shelter. International Quarterly of Community Health Education. v.38,n.issue:4,p.245-9,2018. Disponível em: https://journals.sagepub.com/doi/10.1177/0272684X18781793 Acesso em: 25 ago. 2019.

VET, R. *et al*. Effectiveness of case management for homeless persons: a systematic review. Am J Public Health.v.103,p. e13-e26, 2013.

VIEIRA, A.L. *et al*. Tuberculosis incidence rate among the homeless population: The impact of socio-demographic and health-related variables. Pulmonology. v.24,n.5,p.309-11, 2018. Disponível em: doi: 10.1016/j.pulmoe.2018.05.001. Acesso em: 27 ago. 2019.

VILLA, E.A. *et al*. Sociodemographic profile of women in street situation and vulnerability for the use of psychoactive substances. Rev enferm UFPE on line.v.11,n.5,p.2122-31, 2017. Disponível em: https://periodicos.ufpe.br/revistas/revistaenfermagem/article/view/23367 Acesso em: 27 ago. 2019.

VON, S.F. *et al*. Prevalence of latent tuberculosis in homeless persons: A single-centre cross-sectional study, Germany. PLoS One.v.14,n.3,p.e0214556,2019. Disponível em: doi: 10.1371/journal.pone.0214556. Acesso em: 27 ago. 2019.

WAGNILD, G. M.; YOUNG, H. M. Development and psychometric. Journal of Nursing Measurement.v.1,n.2,p. 165-78, 1993.

WAGNILD, G.M. The Resilience Scale User's Guide for the US English version of the Resilience Scale and the 14-Item Resilience Scale. New York, University Press, 2009.

WANG, H. *et al*. Sintomas depressivos e apoio social entre pessoas vivendo com HIV em Hunan, China . J Assoc Nurses AIDS Care.v. 25,n.6,p. 568 – 76, 2014. Disponível em: DOI:10.1016/j.jana.2014.02.001 Acesso em: 10 jul. 2019.

WATTON, R.; GALIVAN, C. O desafio de prestar cuidados médicos a homens sem-teto. Br J Gen Pract, v.63,n.617,p.659-660, 2013.

WERB, D. *et al*. Intervenções para prevenir o início do uso de drogas injetáveis: uma revisão sistemática. Drug Alcohol Depend.v.133,n.2,p.669-76, 2013.

WINDLE, G.; MARKLAND, D.A.; WOODS, R.T. Examination of a theoretical model of psychological resilience in older age. Aging Ment Health.v.12,n.3,p.285-292, 2008.

WINKELMANN, M.C.C. *et al*. Percepção das pessoas em situação de rua sobre os determinantes sociais da saúde. Rev Enferm UFSM.v.8,n.1,p.88-101,2018. Disponível em: Doi: 10.5902/2179769227259. Acesso em: 27 ago. 2019.

WORLD HEALTH ORGANIZATION. WHO. Global Tuberculosis report 2014. Report No. WHO/HTM/TB/2014.08. Geneva, WHO, 2014.

YIM, L.C. *et al*. Prevalence of Mental Illness among homeless people in Hong Kong.Plos One.v.10,n.10,p.1-26, 2015. Disponível em: https://www.ncbi.nlm.nih.gov/pmc/articles/PMC4618481/pdf/pone.0140940.pdf Acesso em: 27 ago. 2019.

ZENNER, D. *et al*. Conclusão de casos ativos de tuberculose entre os grupos de alto risco em países de baixa incidência. O Jornal Internacional de Tuberculose e Doença Pulmonar, v.17,n.5,p.573-582, 2013.

ZHANG, L. *et al*. High Prevalence of HIV, HCV and Tuberculosis and Associated Risk Behaviours among New Entrants of Methadone Maintenance Treatment Clinics in Guangdong Province, China. PLoS One.v.8,n.10,p.e76931, 2013. Disponível em: doi: 10.1371/journal.pone.0076931 Acesso em: 6 set. 2019.

ZUIM, R.C.B.; TRAJMAN, A. Itinerário terapêutico de doentes com tuberculose vivendo em situação de rua no Rio de Janeiro. Physis: Revista da Saúde Coletiva. v.28,n.2, p.1-19, 2018. Disponível em: http://www.scielo.br/pdf/physis/v28n2/0103-7331-physis-28-02-e280205.pdf Acesso em: 27 ago. 2019.

APÊNDICES E ANEXOS

APÊNDICE A

TERMO DE CONSENTIMENTO LIVRE E ESCLARECIDO

Você está sendo convidado (a) à participar de uma pesquisa intitulada PROMOVENDO SAÚDE E PREVENINDO DOENÇAS EM CASAS DE APOIO PARA PESSOAS EM SITUAÇÃO DE RUA que está sendo desenvolvida por Anna Cláudia Freire de Araújo Patrício, doutoranda em Enfermagem pela Universidade Federal do Rio Grande do Norte .Responsabilizo-me em cumprir as exigências contidas nos termos dos incisos IV-3 e IV-5 da Resolução 466/2012 do Conselho Nacional de Saúde.

O presente estudo possui objetivo geral: Analisar condições de saúde que levam as pessoas em situação de rua à vulnerabilidade. Objetivos específicos: Caracterizar pessoas em situação de rua quanto as variáveis: gênero, idade, estado civil/presença de companheiro, filhos, religião, tempo e motivo de moradia na rua, procura periódica por serviço de saúde, doenças prévias, manifestações clínicas, uso de medicamentos, drogas ilícitas; Identificar a presença de: depressão, ansiedade, transtornos mentais comuns, desesperança, resiliência, HIV, sífilis, hepatite b e c de pessoas em situação de rua; Validar por meio da Teoria de Resposta ao Item os questionários sobre vulnerabilidade ao HIV, sífilis e outras Infecções Sexualmente Transmissíveis, bem como Tuberculose de pessoas em situação de rua; Averiguar comportamentos vulneráveis a infecção ao HIV, sífilis e outras Infecções Sexualmente Transmissíveis, bem como Tuberculose de pessoas em situação de rua; Verificar a influência de fatores psíquicos, sociodemográficos e comportamentais sobre o HIV e a tuberculose.

Esse estudo justifica-se pela precariedade de assistência a saúde direcionada as pessoas em situação de rua, fragilidade de políticas públicas, estimativa incerta do quantitativo de Pessoas em Situação de Rua, apoio social carente, discriminação, estigma, maior exposição a comportamentos vulneráveis. Sendo assim, a enfermagem como profissão ética e atuante na equipe multiprofissional dos Centros de Atendimento, Acolhimento e Casas de Apoio de Pessoas em Situação de Rua pode investigar fatores relacionados a saúde e doença destas pessoas, especificamente sobre saúde mental, HIV, sífilis, hepatite, tuberculose, contribuindo de forma social para redução de agravos relacionados a fragilidade da assistência à saúde nesta população.

Considerando que as condições de vulnerabilidade vivenciadas pela população em situação de rua envolvem questões psicossociais geradoras de sofrimentos físicos e emocionais, possibilitam riscos maiores à saúde, representa-se como estratégia ímpar fornecer assistência e cuidado a saúde, na perspectiva da detecção precoce de agravos e prevenção de doenças.

Esta pesquisa irá contribuir como auxílio nas atividades realizadas na casa de apoio as pessoas em situação de rua no Município de João Pessoa/PB, proporcionando um retrato das condições de saúde na perspectiva de intervir nas mesmas, direcionando tomadas de decisões pela equipe da pesquisa e instigando gestores e profissionais de saúde, contribuindo para o controle e/ou redução dos agravos mais frequentes relacionados a esta população.

Caso participe da pesquisa, responderá a questionários com perguntas sociodemográficas, clínicas e práticas vulneráveis a infecção ao HIV/aids, sífilis e tuberculose, Escalas de Ansiedade, Depressão, Desesperança, Resiliência, além de realizar teste rápido para HIV, sífilis, hepatite B e hepatite C. A coleta de dados será realizada em momento, condição e local adequados, dentro do local de coleta de dados que será a Casa da Acolhida para pessoas em situação de rua, localizado na Rua Capitão José Pessoa, número 65, no bairro de Jaguaribe e no Centro de Referência Especializado para População em Situação de Rua (Creas Pop), localizado na Rua Treze de Maio, 508 – Centro, João Pessoa,Paraíba, Brasil.

Buscaremos com essa pesquisa levar, através de uma produção científica, mais conhecimento teórico e prático sobre as condições de saúde de pessoas em situação de rua.

Salienta-se que segundo a Resolução 466/12 toda pesquisa que envolve seres humanos oferece riscos. Não obstante os riscos potenciais, as pesquisas envolvendo seres humanos serão admissíveis quando oferecerem elevada possibilidade de gerar conhecimento para entender, prevenir ou aliviar um problema que afete o bem-estar dos sujeitos da pesquisa e dos outros indivíduos.

Nesse sentido, o referido estudo pode oferecer desconforto de origem psicológica, emocional uma vez que o sujeito da pesquisa será submetido a questionários contendo informações sociodemográficas, dados clínicos e práticas/comportamentos vulneráveis ao HIV/aids, sífilis e tuberculose, ausculta respiratória através de técnicas de exame físico, testes rápidos para HIV, sífilis, Hepatite B e Hepatite C. Para minimizar esse risco, a coleta de dados será realizada individualmente e em um local reservado.

Ressalta-se durante o exame físico (ausculta respiratória) e testes rápidos pode ocorrer desconforto e incômodo com o posicionamento ou o próprio exame. Para minimizar esse risco, a coleta de dados será realizada individualmente e em um local reservado adequado para avaliação clínica: ambiente arejado e iluminado, cadeira. Além disso, há o risco biológico para aqueles envolvendo sangue ou fluídos (testes rápidos), mas para minimizar estes riscos todos os exames serão realizados com o uso de Equipamentos de Proteção Individual e será realizada a higienização das mãos ao atender cada participante da pesquisa. As lancetas e kits de avaliação são descartáveis e serão utilizados de forma individual, descartados em local adequado, não havendo risco de contaminação.

Quanto aos testes rápidos os sujeitos da pesquisa recebrão informações/orientações/aconselhamentos antes do exame e após o resultado. Este resultado será revelado de forma sigilosa e individual em ambiente fechado dentro do próprio local de coleta de dados. Para os resultados positivos além de orientações fornecidas pelos pesquisadores, o sujeito da pesquisa será oaconselhado a procurar o Hospital de Referência para Doenças Infectocontagiosas – Clementino Fraga, local destinado especificamente a tratar estas doenças no município de João Pessoa/PB. Além disso, ressalta-se a presença de psicólogo e assistente social do local de coleta de dados.

Caso o sujeito da pesquisa sofra algum dano comprovadamente em decorrência deste estudo assume-se o compromisso de interromper imediatamente a pesquisa, e se necessário, será garantido ao participante do estudo acompanhamento e assistência imediata e/ou integral a ser fornecida pela equipe de saúde da instituição que os coordenadores estão vinculados. Além disso, salienta-se o direito do participante, quando preciso, ter acompanhamento posterior e plena liberdade de recusar-se a participar ou retirar o consentimento em qualquer fase da pesquisa, sem penalização alguma, sendo garantida a manutenção do sigilo e da sua privacidade durante todas as fases da mesma.

Informa-se ainda que o risco se justifica pela importância do benefício esperado, haja vista que pesquisas como estas podem oferecer grandes contribuições à sociedade e a comunidade científica de um modo geral na medida em que seus resultados poderão contribuir para a construção de estratégias que possibilitem uma melhor assistência as pessoas em situação de rua.

Ressalta-se que a participação neste estudo é estritamente voluntária. Portanto não será realizado o pagamento de nenhum valor a título de reembolsado ou o pagamento de qualquer compensação financeira.

Durante todo o período da pesquisa você poderá tirar suas dúvidas ligando para a Anna Cláudia Freire de Araújo Patrício no celular (83)993111814 ou no Programa de Pós Graduação em Enfermagem da Universidade Federal do Rio Grande do Norte, localizado na Av Salgado Filho, Lagoa Nova, Natal/RN. Fone: (84) 3215-3196.

Garantimos que você receberá uma via do Termo de Consentimento Livre e Esclarecido e, em caso de danos ou desistência em participar da pesquisa, garantimos o ressarcimento e cobertura das despesas tidas por você, bem como indenização diante de eventuais danos decorrentes da pesquisa.

Solicito sua autorização para apresentar os resultados deste estudo em eventos científicos e publicar em revista científica. Será garantida a privacidade dos dados e informações fornecidas, que se manterão em caráter confidencial. Por ocasião da publicação dos resultados, seu nome será mantido em completo sigilo. Os dados serão guardados pelo pesquisador responsável por essa pesquisa em local seguro e por um período de 05 anos.

A sua participação na pesquisa é voluntária, e, portanto o (a) senhor (a) não é obrigado (a) a fornecer as informações e/ou colaborar com as atividades solicitadas pela pesquisadora.

A pesquisadora responsável estará a sua disposição para qualquer esclarecimento que considere necessário, em qualquer etapa da pesquisa.

Eu ____, declaro que fui devidamente esclarecido (a) e dou o meu consentimento para participar da pesquisa e para publicação dos resultados. Estou ciente que receberei uma via desse documento. Fica registrado também que tenho conhecimento de que essas informações, dados ou materiais serão usados pela responsável da pesquisa com propósitos científicos.

João Pessoa, _______ / _________________/201__

____

Assinatura do Participante da Pesquisa

____

Assinatura de Testemunha

Como pesquisador responsável pelo estudo Anna Cláudia Freire de Araújo Patrício declaro que assumo a inteira responsabilidade de cumprir fielmente os procedimentos metodologicamente e direitos que foram esclarecidos e assegurados ao participante desse estudo, assim como manter sigilo e confidencialidade sobre a identidade do mesmo.

Declaro ainda estar ciente que, na inobservância do compromisso ora assumido, estarei infringindo as normas e diretrizes propostas pela Resolução 466/12 do Conselho Nacional de Saúde – CNS, que regulamenta as pesquisas envolvendo o ser humano.

_____________________________________ ____________________________

Assinatura da Pesquisadora Responsável Assinatura do(a) aluno participante da pesquisa

Pesquisadora Responsável: Anna Cláudia Freire de Araújo Patrício no celular (83)993111814 ou no Programa de Pós Graduação em Enfermagem da Universidade Federal do Rio Grande do Norte, localizado na Av Salgado Filho, Lagoa Nova, Natal/RN. Fone: (84) 3215-3196. E-mail: claudia.freirearaujo@gmail.com. Comitê de Ética em Pesquisa/Unipê. Endereço: Campus do UNIPÊ: BR 230, Km 22, Água Fria, João Pessoa–PB. Reitoria, sala 401. Fone: (83) 2106-9266. E-mail: cep@unipe.br.

APÊNDICE B – DADOS SOCIODEMOGRÁFICOS

**DADOS SOCIODEMOGRÁFICOS**	
GÊNERO<br>F ( )<br>M ( )	IDADE
TEMPO QUE É PESSOA EM SITUAÇÃO DE RUA:	MOTIVO PELO QUAL DECIDIU SER PESSOA EM SITUAÇÃO DE RUA:<br><br>CONFRONTO FAMILIAR ( )<br>USO DE DROGAS ( )<br>DESEJO DE LIBERDADE ( )<br>OUTROS:
RELIGIÃO<br>EVANGÉLICA ( )<br>CATÓLICA ( )<br>ESPÍRITA ( )<br>OUTRO:	ATIVIDADE REMUNERADA:
NÚMERO DE FILHOS<br>0 ( ) 1 ( ) 2 ( ) 3 ( ) 4 ( ) 5( ) MAIS DE 5 ( )	ESTADO CIVIL/COMPANHEIRO<br><br>SOLTEIRO ( )<br>CASADO ( )<br>DIVORCIADO ( )<br>VIÚVO ( )
PROCURA PERIÓDICA POR SERVIÇO DE SAÚDE<br><br>A CADA 3 MESES ( )<br>A CADA 6 MESES ( )<br>ANUALMENTE ( )<br>2 EM 2 ANOS ( )<br>OUTROS	ACESSO AO SERVIÇO DE SAÚDE.<br>ÓTIMO ( )<br>BOM ( )<br>REGULAR ( )<br>NÃO TENHO ACESSO ( )<br>OUTROS:

APÊNDICE C - DADOS CLÍNICOS E DE VULNERABILIDADE AO HIV, SÍFILIS E TUBERCULOSE . TESTES RÁPIDOS PARA HIV, SÍFILIS E HEPATITE B E C.

**VARIÁVEIS PARA VULNERABILIDADE AO HIV E A SÍFILIS**	
UTILIZA MEDICAMENTOS<br>SIM ( )<br>NÃO ( )<br>QUAL?	SINTOMATOLOGIA:<br>Taquicardia ( )<br>Dor ( )<br>Tristeza ( )<br>Tosse ( )<br>Outros ( )
	ETILISMO<br>SIM ( )<br>NÃO ( )
UTILIZA DROGA<br>SIM ( )	QUAL DROGA<br>MACONHA ( )

NÃO ( )	CRACK ( )<br>OUTRA? QUAL?
ANTECEDENTES FAMILIARES:<br>Diabetes mellitus ( )<br>Acidente vascular encefálico (AVE ( )<br>Hipertensão arterial ( )<br>Infarto Agudo do Miocardio ( )<br>OUTRO:	ANTECEDENTES PESSOAIS<br>Diabetes mellitus ( )<br>Acidente vascular encefálico (AVE ( )<br>Hipertensão arterial ( )<br>Infarto Agudo do Miocardio ( )<br>Tuberculose ( )<br>Pneumonia ( )<br><br>OUTRO:
**TESTES RÁPIDOS**	
HIV<br>POSITIVO ( )<br>NEGATIVO ( )	SÍFILIS<br>POSITIVO ( )<br>NEGATIVO ( )
HEPATITE B<br>POSITIVO ( )<br>NEGATIVO ( )	HEPATITE C<br>POSITIVO ( )<br>NEGATIVO ( )
IDADE DA PRIMEIRA RELAÇÃO SEXUAL:	
DIAGNÓSTICO E TRATAMENTO PRÉVIO DE ALGUMA DOENÇA SEXUALMENTE TRANSMISSÍVEL<br>Candidíase ( )<br>Tricomoníase ( )<br>Gonorreia ( )<br>Vaginose ( )<br>Sífilis ( )<br>Outros ( ) Quais:	UTILIZA PRESERVATIVO NO SEXO:<br>ANAL<br>SIM ( ) NÃO ( ) AS VEZES ( )<br>VAGINAL<br>SIM ( ) NÃO ( ) AS VEZES ( )<br>ORAL<br>SIM ( ) NÃO ( ) AS VEZES ( )
RAZÃO PARA NÃO USAR PRESERVATIVO<br>PREFERE PELE NA PELE ( )<br>CONFIANÇA ( )<br>PODEM PENSAR QUE SOU HIV POSITIVO ( )<br>PRESERVATIVO NÃO ESTÁ DISPONÍVEL ( )	TIPO DE PARCEIROS SEXUAIS:<br>TRABALHADORES DO SEXO ( )<br>NAMORADA ( )<br>ESPOSA ( )<br>PARCEIRO CASUAL ( )
TEM ATIVIDADE SEXUAL COM USUÁRIO DE DROGA<br>SIM ( )<br>NÃO ( )	TEM RELAÇÃO SEXUAL EM TROCA DE DINHEIRO<br>SIM ( )<br>NÃO ( )
JÁ APRESENTOU ALGUM SINTOMA DE INFECÇÃO SEXUALMENTE TRANSMISSÍVEL (corrimento, ardor, coceira)?<br>SIM ( )<br>NÃO ( )<br>QUAL?	QUAIS OS TIPOS DE SEXO VOCÊ REALIZA?<br>ORAL ( )<br>ANAL ( )<br>VAGINAL ( )
POSSUI QUANTOS PARCEIROS<br>1 ( )<br>2 ( )<br>3 ( )<br>MAIS DE 3 ( )	SENTE DOR DURANTE O ATO SEXUAL<br>SIM ( )<br>NÃO ( )
JÁ REALIZOU SEXO COM PARCEIRO COM ALGUM FERIMENTO NA REGIÃO GENITAL OU ANAL?<br>SIM ( )<br>NÃO ( )	SEU PARCEIRO JÁ APRESENTOU ALGUM SINTOMA DE INFECÇÃO SEXUALMENTE TRANSMISSÍVEL?<br>SIM ( ) NÃO ( ) QUAL?

COMPARTILHA PERFUROCORTANTES?<br>SIM ( )<br>NÃO ( )	REUTILIZA SERINGAS/AGULHAS?<br>SIM ( )<br>NÃO ( )
JÁ SOFREU VIOLÊNCIA FÍSICA?<br>SIM ( )<br>NÃO ( )	JÁ SOFREU VIOLÊNCIA SEXUAL?<br>SIM ( )<br>NÃO ( )
**VARIÁVEIS DE VULNERABILIDADE A TUBERCULOSE**	
TOMA BANHO QUANTAS VEZES AO DIA<br>0 ( )<br>1 ( )<br>2 ( )<br>3 ( )<br>MENSALMENTE ( )<br>QUINZENALMENTE ( )<br>SEMANALMENTE ( )	ASPECTO SUJO<br>SIM ( )<br>NÃO ( )
ESCOVA OS DENTES QUANTAS VEZES AO DIA<br>0 ( ) 1( ) 2( ) 3( )<br>MENSALMENTE ( )<br>QUINZENALMENTE ( )<br>SEMANALMENTE ( )	COSTUMA LEVAR CHUVA?<br>SIM ( )<br>NÃO ( )
TOSSE ( )<br>SECREÇÃO ( )<br>CHIADO NO PEITO ( )	FEBRE PERIODICAMENTE?<br>SIM ( )<br>NÃO ( )
TABAGISMO:<br>SIM ( )<br>NÃO ( )<br>QUANTOS CIGARROS POR DIA?	

**RESPIRATÓRIO**	
**Ritmo**<br>Cheyne-Stokes ( ) Biot ( ) Dispneico ( ) Kussmaul ( ) Eupneia ( )	
**Sons respiratórios**<br>Murmúros vesiculares<br>Audíveis ( )<br>Diminuídos ( )	Ruídos adventícios<br>Creptações ( )<br>Roncos ( )<br>Sibilos ( )<br>Local:
**VARIÁVEIS SOBRE SAÚDE MENTAL**	TONTURA ( )<br>DESMAIOS ( )<br>ESQUECIMENTO ( )<br>VISÃO TURVA ( )<br>CEFALEIA ( )

ANEXO A - ESCALA SRQ 20

TESTE 3: SRQ 20 - Self Report Questionnaire.

Teste que avalia o sofrimento mental. Por favor, leia estas instruções antes de preencher as questões abaixo. É muito importante que todos que estão preenchendo o questionário sigam as mesmas instruções.

Instruções

Estas questões são relacionadas a certas dores e problemas que podem ter lhe incomodado nos últimos 30 dias. Se você acha que a questão se aplica a você e você teve o problema descrito nos últimos 30 dias responda SIM. Por outro lado, se a questão não se aplica a você e você não teve o problema nos últimos 30 dias, responda NÃO.

OBS: Lembre-se que o diagnóstico definitivo só pode ser fornecido por um profissional.

PERGUNTAS	RESPOSTAS	
1- Você tem dores de cabeça freqüente?	SIM	NÃO
2- Tem falta de apetite?	SIM	NÃO
3- Dorme mal?	SIM	NÃO
4- Assusta-se com facilidade?	SIM	NÃO
5- Tem tremores nas mãos?	SIM	NÃO
6- Sente-se nervoso (a), tenso (a) ou preocupado (a)?	SIM	NÃO
7- Tem má digestão?	SIM	NÃO
8- Tem dificuldades de pensar com clareza?	SIM	NÃO
9- Tem se sentido triste ultimamente?	SIM	NÃO
10- Tem chorado mais do que costume?	SIM	NÃO
11- Encontra dificuldades para realizar com satisfação Suas atividades diárias?	SIM	NÃO
12- Tem dificuldades para tomar decisões?	SIM	NÃO
13- Tem dificuldades no serviço (seu trabalho é penoso, lhe causa- sofrimento?)	SIM	NÃO
14- É incapaz de desempenhar um papel útil em sua vida?	SIM	NÃO
15- Tem perdido o interesse pelas coisas?	SIM	NÃO
[illegible]	SIM	NÃO
17- Tem tido idéia de acabar com a vida?	SIM	NÃO
18- Sente-se cansado (a) o tempo todo?	SIM	NÃO
19- Você se cansa com facilidade?	SIM	NÃO
20- Têm sensações desagradáveis no estomago?	SIM	NÃO

RESULTADO

Se o resultado for ≥ 7 (maior ou igual a sete respostas SIM) está comprovado sofrimento mental

ANEXO B – ANSIEDADE

Abaixo está uma lista de sintomas comuns de ansiedade. Por favor, leia cuidadosamente cada item da lista. Identifique o quanto você tem sido incomodado por cada sintoma durante a **última semana, incluindo hoje**, colocando um "x" no espaço correspondente, na mesma linha de cada sintoma.

	**Absolutamente não**	**Levemente** Não me incomodou muito	**Moderadamente** Foi muito desagradável mas pude suportar	**Gravemente** Dificilmente pude suportar
1. Dormência ou formigamento.				
2. Sensação de calor.				
3. Tremores nas pernas.				
4. Incapaz de relaxar.				
5. Medo que aconteça o pior.				
6. Atordoado ou tonto.				
7. Palpitação ou aceleração do coração.				
8. Sem equilíbrio.				
9. Aterrorizado.				
10. Nervoso.				
11. Sensação de sufocação.				
12. Tremores nas mãos.				
13. Trêmulo.				
14. Medo de perder o controle.				
15. Dificuldade de respirar.				
16. Medo de morrer.				
17. Assustado.				
18. Indigestão ou desconforto no abdômen.				
19. Sensação de desmaio.				
20. Rosto afogueado.				
21. Suor (não devido ao calor).				

"Traduzido e adaptado por permissão de The Psychological Corporation, U.S.A. Direitos reservados ©1991, a Aaron T. Beck.
Tradução para a língua portuguesa. Direitos reservados ©1993 a Aaron T. Beck. Todos os direitos reservados."
Tradução e adaptação brasileira, 2001, Casa do Psicólogo Livraria e Editora Ltda. BAI é um logotipo da Psychological Corporation.

ANEXO C – DEPRESSÃO DE BECK

Este questionário consiste em 21 grupos de afirmações. Depois de ler cuidadosamente cada grupo, faça um círculo em torno do número (0, 1, 2 ou 3) próximo à afirmação, em cada grupo, que descreve **melhor** a maneira que você tem se sentido na **última semana, incluindo hoje**. Se várias afirmações num grupo parecerem se aplicar igualmente bem, faça um círculo em cada uma. **Tome o cuidado de ler todas as afirmações, em cada grupo, antes de fazer a sua escolha.**

1
0 Não me sinto triste.
1 Eu me sinto triste.
2 Estou sempre triste e não consigo sair disto.
3 Estou tão triste ou infeliz que não consigo suportar.

2
0 Não estou especialmente desanimado quanto ao futuro.
1 Eu me sinto desanimado quanto ao futuro.
2 Acho que nada tenho a esperar.
3 Acho o futuro sem esperança e tenho a impressão de que as coisas não podem melhorar.

3
0 Não me sinto um fracasso.
1 Acho que fracassei mais do que uma pessoa comum.
2 Quando olho para trás, na minha vida, tudo o que posso ver é um monte de fracassos.
3 Acho que, como pessoa, sou um completo fracasso.

4
0 Tenho tanto prazer em tudo como antes.
1 Não sinto mais prazer nas coisas como antes.
2 Não encontro um prazer real em mais nada.
3 Estou insatisfeito ou aborrecido com tudo.

5
0 Não me sinto especialmente culpado.
1 Eu me sinto culpado grande parte do tempo.
2 Eu me sinto culpado na maior parte do tempo.
3 Eu me sinto sempre culpado.

6
0 Não acho que esteja sendo punido.
1 Acho que posso ser punido.
2 Creio que vou ser punido.
3 Acho que estou sendo punido.

7
0 Não me sinto decepcionado comigo mesmo.
1 Estou decepcionado comigo mesmo.
2 Estou enojado de mim.
3 Eu me odeio.

8
0 Não me sinto de qualquer modo pior que os outros.
1 Sou crítico em relação a mim por minhas fraquezas ou erros.
2 Eu me culpo sempre por minhas falhas.
3 Eu me culpo por tudo de mal que acontece.

9
0 Não tenho quaisquer idéias de me matar.
1 Tenho idéias de me matar, mas não as executaria.
2 Gostaria de me matar.
3 Eu me mataria se tivesse oportunidade.

10
0 Não choro mais que o habitual.
1 Choro mais agora do que costumava.
2 Agora, choro o tempo todo.
3 Costumava ser capaz de chorar, mas agora não consigo, mesmo que o queira.

11
0 Não sou mais irritado agora do que já fui.
1 Fico aborrecido ou irritado mais facilmente do que costumava.
2 Agora, eu me sinto irritado o tempo todo.
3 Não me irrito mais com coisas que costumavam me irritar.

12
0 Não perdi o interesse pelas outras pessoas.
1 Estou menos interessado pelas outras pessoas do que costumava estar.
2 Perdi a maior parte do meu interesse pelas outras pessoas.
3 Perdi todo o interesse pelas outras pessoas.

13
0 Tomo decisões tão bem quanto antes.
1 Adio as tomadas de decisões mais do que costumava.
2 Tenho mais dificuldades de tomar decisões do que antes.
3 Absolutamente não consigo mais tomar decisões.

14
0 Não acho que de qualquer modo pareço pior do que antes.
1 Estou preocupado em estar parecendo velho ou sem atrativo.
2 Acho que há mudanças permanentes na minha aparência, que me fazem parecer sem atrativo.
3 Acredito que pareço feio.

15
0 Posso trabalhar tão bem quanto antes.
1 É preciso algum esforço extra para fazer alguma coisa.
2 Tenho que me esforçar muito para fazer alguma coisa.
3 Não consigo mais fazer qualquer trabalho.

16
0 Consigo dormir tão bem como o habitual.
1 Não durmo tão bem como costumava.
2 Acordo 1 a 2 horas mais cedo do que habitualmente e acho difícil voltar a dormir.
3 Acordo várias horas mais cedo do que costumava e não consigo voltar a dormir.

17
0 Não fico mais cansado do que o habitual.
1 Fico cansado mais facilmente do que costumava.
2 Fico cansado em fazer qualquer coisa.
3 Estou cansado demais para fazer qualquer coisa.

18
0 O meu apetite não está pior do que o habitual.
1 Meu apetite não é tão bom como costumava ser.
2 Meu apetite é muito pior agora.
3 Absolutamente não tenho mais apetite.

19
0 Não tenho perdido muito peso se é que perdi algum recentemente.
1 Perdi mais do que 2 quilos e meio.
2 Perdi mais do que 5 quilos.
3 Perdi mais do que 7 quilos.

Estou tentando perder peso de propósito, comendo menos: Sim______Não______

20
0 Não estou mais preocupado com a minha saúde do que o habitual.
1 Estou preocupado com problemas físicos, tais como dores, indisposição do estômago ou constipação.
2 Estou muito preocupado com problemas físicos e é difícil pensar em outra coisa.
3 Estou tão preocupado com meus problemas físicos que não consigo pensar em qualquer outra coisa.

21
0 Não notei qualquer mudança recente no meu interesse por sexo.
1 Estou menos interessado por sexo do que costumava.
2 Estou muito menos interessado por sexo agora.
3 Perdi completamente o interesse por sexo.

ANEXO D – ESCALA DE DESESPERANÇA

Este questionário consiste em 20 afirmações. Por favor, leia as afirmações cuidadosamente, uma por uma. Se a afirmação descreve a sua atitude na **última semana, incluindo hoje**, escureça o círculo com "C", indicando CERTO, na mesma linha da afirmação. Se a afirmação não descreve a sua atitude, escureça o círculo com "E", indicando ERRADO, na mesma linha da afirmação. **Por favor, procure ler cuidadosamente cada afirmação.**

1. Penso no futuro com esperança e entusiasmo.	Ⓒ	Ⓔ
2. Seria melhor desistir, porque nada há que eu possa fazer para tornar as coisas melhores para mim.	Ⓒ	Ⓔ
3. Quando as coisas vão mal, me ajuda saber que elas não podem continuar assim para sempre.	Ⓒ	Ⓔ
4. Não consigo imaginar que espécie de vida será a minha em dez anos.	Ⓒ	Ⓔ
5. Tenho tempo suficiente para realizar as coisas que quero fazer.	Ⓒ	Ⓔ
6. No futuro, eu espero ter sucesso no que mais me interessa.	Ⓒ	Ⓔ
7. Meu futuro me parece negro.	Ⓒ	Ⓔ
8. Acontece que tenho uma sorte especial e espero conseguir mais coisas boas da vida do que uma pessoa comum.	Ⓒ	Ⓔ
9. Simplesmente não consigo aproveitar as oportunidades e não há razão para que consiga, no futuro.	Ⓒ	Ⓔ
10. Minhas experiências passadas me prepararam bem para o futuro.	Ⓒ	Ⓔ
11. Tudo o que posso ver à minha frente é mais desprazer do que prazer.	Ⓒ	Ⓔ
12. Não espero conseguir o que realmente quero.	Ⓒ	Ⓔ
13. Quando penso no futuro, espero ser mais feliz do que sou agora.	Ⓒ	Ⓔ
14. As coisas simplesmente não se resolvem da maneira que eu quero.	Ⓒ	Ⓔ
15. Tenho uma grande fé no futuro.	Ⓒ	Ⓔ
16. Nunca consigo o que quero. Assim, é tolice querer qualquer coisa.	Ⓒ	Ⓔ
17. É pouco provável que eu vá obter qualquer satisfação real, no futuro.	Ⓒ	Ⓔ
18. O futuro me parece vago e incerto.	Ⓒ	Ⓔ
19. Posso esperar mais tempos bons do que maus.	Ⓒ	Ⓔ
20. Não adianta tentar realmente obter algo que quero, porque provavelmente não vou conseguir.	Ⓒ	Ⓔ

ANEXO E - ESCALA DE RESILIÊNCIA

Marque o quanto você concorda ou discorda com as seguintes afirmações:

	DISCORDO			NEM CONCORDO NEM DISCORDO	CONCORDO		
	Totalmente	Muito	Pouco		Pouco	Muito	Totalmente
1. Quando eu faço planos, eu levo eles até o fim.	1	2	3	4	5	6	7
2. Eu costumo lidar com os problemas de uma forma ou de outra.	1	2	3	4	5	6	7
3. Eu sou capaz de depender de mim mais do que qualquer outra pessoa.	1	2	3	4	5	6	7
4. Manter interesse nas coisas é importante para mim.	1	2	3	4	5	6	7
5. Eu posso estar por minha conta se eu precisar.	1	2	3	4	5	6	7
6. Eu sinto orgulho de ter realizado coisas em minha vida.	1	2	3	4	5	6	7
7. Eu costumo aceitar as coisas sem muita preocupação.	1	2	3	4	5	6	7
8. Eu sou amigo de mim mesmo.	1	2	3	4	5	6	7
9. Eu sinto que posso lidar com várias coisas ao mesmo tempo.	1	2	3	4	5	6	7
10. Eu sou determinado.	1	2	3	4	5	6	7
11. Eu raramente penso sobre o objetivo das coisas.	1	2	3	4	5	6	7
12. Eu faço as coisas um dia de cada vez.	1	2	3	4	5	6	7
13. Eu posso enfrentar tempos difíceis porque já experimentei dificuldades antes.	1	2	3	4	5	6	7
14. Eu sou disciplinado.	1	2	3	4	5	6	7
15. Eu mantenho interesse nas coisas.	1	2	3	4	5	6	7
16. Eu normalmente posso achar motivo para rir.	1	2	3	4	5	6	7
17. Minha crença em mim mesmo me leva a atravessar tempos difíceis.	1	2	3	4	5	6	7
18. Em uma emergência, eu sou uma pessoa em quem as pessoas podem contar.	1	2	3	4	5	6	7
19. Eu posso geralmente olhar uma situação de diversas maneiras.	1	2	3	4	5	6	7
20. Às vezes eu me obrigo a fazer coisas querendo ou não.	1	2	3	4	5	6	7
21. Minha vida tem sentido.	1	2	3	4	5	6	7
22. Eu não insisto em coisas as quais eu não posso fazer nada sobre elas.	1	2	3	4	5	6	7
23. Quando eu estou numa situação difícil, eu normalmente acho uma saída.	1	2	3	4	5	6	7
24. Eu tenho energia suficiente para fazer o que eu tenho que fazer.	1	2	3	4	5	6	7
25. Tudo bem se há pessoas que não gostam de mim.	1	2	3	4	5	6	7

Escala de Resiliência de Wagnild e Young (1993), versão adaptada para o português por Pesce, Assis, Avanci, Santos, Malaquias, & Carvalhaes (2005).

ANEXO F - ESCALA DE DEPRESSÃO DE HAMILTON

Introdução

Gostaria de lhe fazer algumas perguntas sobre a última semana. Como você tem se sentido desde a última (dia da semana)? Se paciente ambulatorial: você tem trabalhado? Se não: especifique por que não?

1 - Como tem estado seu humor na última semana?
Você tem se sentido para baixo ou deprimido?
Triste? Sem esperança?
Na última semana, com que freqüência você se sentiu (utilize a palavra referida pelo paciente)? Todos os dias? O dia inteiro?
Você tem chorado?

HUMOR DEPRESSIVO (tristeza, desesperança, desamparo, inutilidade)

0 ( ) Ausente.
1 ( ) Sentimentos relatados somente se perguntados.
2 ( ) Sentimentos relatados espontaneamente, com palavras.
3 ( ) Comunica os sentimentos não com palavras, isto é, com a expressão facial; a postura, a voz e a tendência ao choro.
4 ( ) O paciente comunica quase exclusivamente esses sentimentos, tanto em seu relato verbal como na comunicação não-verbal.

Se pontuou de 1-4, pergunte: há quanto tempo você tem se sentido dessa maneira?

2 - Você tem se sentido especialmente autocrítico nesta última semana, sentindo que fez coisas erradas ou decepcionou outras pessoas?
SE SIM: Quais foram esses pensamentos?
Você tem se sentido culpado em relação a coisas que fez ou não fez?
Você tem pensado que, de alguma forma, você é responsável pela sua depressão?
Você sente que está sendo punido ficando doente?

SENTIMENTOS DE CULPA

0 ( ) Ausente.
1 ( ) Auto-recriminação, acha que decepcionou outras pessoas.
2 ( ) Idéias de culpa ou ruminações de erros ou ações pecaminosas (más) no passado.
3 ( ) Paciente acha que a doença atual é uma punição (castigo). Delírio de culpa.
4 ( ) Ouve vozes que o acusam ou denunciam e/ou tem alucinações visuais ameaçadoras.

3 - Nesta última semana, você teve pensamentos de que não vale a pena, viver ou que você estaria melhor morto? Ou pensamentos de se machucar ou até de se matar?
SE SIM: O que você tem pensado sobre isso? Você já se machucou?

SUICÍDIO

0 ( ) Ausente.
1 ( ) Acha que não vale a pena viver.
2 ( ) Deseja estar morto ou pensa em um..
3 ( ) Idéias ou atitudes suicidas.
4 ( ) Tentativas de suicídio.

4 - Como tem sido seu sono na última semana?
Você teve alguma dificuldade em iniciar o sono? Após se deitar, quanto tempo levava para conseguir dormir?
Em quantas noites nesta última semana você teve problemas para iniciar o sono?

INSÔNIA INICIAL

0 ( ) Sem dificuldades para iniciar o sono.
1 ( ) Queixa de dificuldade ocasional para iniciar o sono, ou seja, mais que meia hora.
2 ( ) Queixa-se de dificuldade para iniciar o sono todas as noites.

5 - Durante esta última semana, você tem acordado no meio da noite?
SE SIM: Você sai da cama? O que você faz? (somente vai ao banheiro?)
Quando volta para a cama, você volta a dormir logo?
Você sente que seu sono é agitado ou perturbado em algumas noites?

INSÔNIA INTERMEDIÁRIA

0 ( ) Sem dificuldade.
1 ( ) Queixa-se de agitação e perturbação durante a noite.
2( ) Acorda durante a noite - qualquer saída da cama (exceto por motivos de necessidade fisiológica).

6 - A que horas você tem acordado pela manhã na última semana?
Se cedo: Acorda com despertador ou sozinho? A que horas você normalmente acorda (ou seja, antes de ficar deprimido)?

INSÔNIA TARDIA

0 ( ) Sem dificuldade.
1 ( ) Acorda durante a madrugada, mas volta a dormir.
2 ( ) Não consegue voltar a dormir se levantar da cama durante a noite.

7 - Como você tem passado seu tempo na última semana (quando não está no trabalho)?
Você se sente interessado em fazer (essas atividades) ou você tem de se forçar?
Você parou de fazer atividades que costumava fazer? SE SIM: Por quê?

Há alguma coisa que você aguarda ansiosamente?
(no seguimento): Seu interesse voltou ao normal?

TRABALHO E ATIVIDADES

0 ( ) Sem dificuldades.
1 ( ) Pensamentos e sentimentos de incapacidade, fadiga ou fraqueza, relacionado a atividades, trabalho ou passatempos.
2 ( ) Perda de interesse em atividades, passatempos ou trabalho, quer relatado diretamente pelo paciente, quer indiretamente por desatenção, indecisão ou vacilação (sente que precisa se esforçar para o trabalho ou atividades).
3 ( ) Diminuição no tempo gasto em atividades ou queda de produtividade. No hospital, o paciente se ocupa por menos de três horas por dia em atividades (trabalho hospitalar ou passatempos), com exceção das tarefas rotineiras da enfermaria.
4 ( ) Parou de trabalhar devido à doença atual. No hospital, sem atividades, com exceção das tarefas rotineiras da enfermaria, ou se não consegue realizá-las sem ajuda.

8 - Avaliação baseada na observação durante a entrevista.

RETARDO (lentificação do pensamento e da fala, dificuldade de concentração, diminuição da atividade motora)

0 ( ) Pensamento e fala normal.
1 ( ) Lentificação discreta à entrevista.
2 ( ) Lentificação óbvia durante à entrevista.
3 ( ) Entrevista difícil.
4 ( ) Estupor completo.

9 - Avaliação baseada na observação durante a entrevista.

AGITAÇÃO

0 ( ) Nenhuma.
1 ( ) Inquietação.
2 ( ) Mexe as mãos, cabelos, etc.
3 ( ) Movimenta-se bastante, não consegue permanecer sentado durante a entrevista.
4 ( ) Retorce as mãos rói as unhas, puxa os cabelos, morde os lábios.

10 - Você tem se sentido especialmente tenso ou irritado nesta última semana?
Você tem estado preocupado com coisas pouco importantes, com as quais normalmente não se preocuparia? SE SIM: Com o que, por exemplo?

ANSIEDADE PSÍQUICA

0 ( ) Sem dificuldade.
1 ( ) Tensão e irritabilidade subjetivas.
2 ( ) Preocupa-se com trivialidades.
3 ( ) Atitude apreensiva aparente no rosto ou na fala.
4 ( ) Paciente expressa medo sem ser perguntado.

11 - Na última semana, você sofreu de alguns dos seguintes sintomas físicos?
Leia a lista, parando após cada sintoma para resposta.
O quanto esses sintomas o incomodaram na última semana? Quão intensos foram?
Quanto tempo ou com que freqüência os teve?
Nota: não considerar se claramente relacionados à medicação (por exemplo, boca seca e imipramina)

ANSIEDADE-SOMÁTICA

Concomitantes fisiológicos da ansiedade, como: .
( ) GI: boca seca, flatulência, indigestão, diarréias, cólicas, eructações.
( ) CV: palpitação, cefaléias.
( ) Respiratórios: hiperventilação, suspiros.
( ) Ter de urinar freqüentemente.
( ) Sudorese.
0 ( ) Ausente.
1 ( ) Duvidosos ou trivial: sintomas menores, relatados quando questionados.
2 ( ) Leve: paciente descreve espontaneamente os sintomas, que não são acentuados ou incapacitantes.
3 ( ) Moderada: mais que 2 sintomas e com maior freqüência. São acompanhados de estresse subjetivo e prejudicam o funcionamento normal.
4 ( ) Grave: numerosos sintomas, persistentes e incapacitantes na maior parte do tempo, ou ataques de pânico quase diariamente.

12 - Como tem estado seu apetite nesta última semana? (Como se compara ao seu apetite habitual?) Você tem tido de se forçar a comer?
As outras pessoas têm de insistir para você comer?

SINTOMAS SOMÁTICOS - GASTRINTESTINAIS

0 ( ) Nenhum.
1 ( ) Perda de apetite, mas come sem necessidade de insistência.
2 ( ) Dificuldade para comer se não insistirem.

13 - Como tem estado sua "energia" nesta última semana? Você se sente cansado o tempo todo?
Nesta última semana, você teve dor nas costas, dor de cabeça ou dor muscular?

Nesta última semana, você tem sentido um peso nos membros, nas costas ou na cabeça?

SINTOMAS SOMÁTICOS - GERAIS

0 ( ) Nenhum.
1 ( ) Peso em membros, costas ou cabeça; dor nas costas, cabeça ou muscular. Perda de energia e fatigabilidade.
2 ( ) Qualquer sintoma bem caracterizado e nítido.

14 - Como tem estado seu interesse por sexo nesta semana? (não estou lhe perguntando sobre seu desempenho sexual, mas sobre seu interesse por sexo, isto é, o quanto você tem pensado nisso?)
Houve alguma mudança em seu interesse por sexo (relação à época em que você não estava deprimido)?
Isso é algo em que você tem pensado muito? Se não: isso é pouco habitual para você?

SINTOMAS GENITAIS (como perda de libido, distúrbios menstruais)

0 ( ) Ausentes.
1 ( ) Leves ou infreqüentes: perda de libido, desempenho sexual prejudicado.
2 ( ) Óbvio e graves: perda completa do interesse sexual.

15 - Na última semana, quanto seus pensamentos têm focalizado na sua saúde física ou no funcionamento de seu corpo (comparado ao seu pensamento habitual)
Você se queixa muito de sintomas físicos?
Você tem-se deparado com situações em que pede ajuda para fazer coisas que poderia fazer sozinho?
SE SIM: Como o que, por exemplo? Com que freqüência isso tem ocorrido?

16 - Você perdeu algum peso desde que essa (DEPRESSÃO) começou? SE SIM: Quanto?
SE INCERTO: Você acha que suas roupas estão mais folgadas?
NO SEGUIMENTO: Você voltou a ganhar peso?

PERDA DE PESO (desde o início da doença ou última avaliação):

0 ( ) Sem Perda de peso ou perda de peso NÃO causada pela doença atual.
[illegible]

17 - Avaliação baseada na observação

CRÍTICA (CONSCIÊNCIA DA DOENÇA)

0 ( ) Reconhece estar deprimido e doente OU não estar deprimido no momento.
1 ( ) Reconhece estar, mas atribui a causa a má alimentação, ao clima, ao excesso de trabalho, a um vírus, à necessidade de descanso, etc.
2 ( ) Nega estar doente.

ESCORE TOTAL HAM-17 ITENS

TOTAL:
DATA:
NÚMERO:

ANEXO G

INSTRUMENTO REFERENTE A VULNERABILIDADE AO HIV/AIDS

Siga as instruções abaixo:

1. Leia atentamente as alternativas e marque a que melhor condiz com a sua realidade;
2. Caso haja alguma dúvida, solicite a ajuda do facilitador (a) / pesquisador (a);

1. Em meus relacionamentos ...

- Já tive relação sexual e usei- ou meu parceiro usou-camisinha, todas as vezes que eu transei;
- Nunca tive relação sexual;
- Já tive pelo menos uma relação sexual sem usar camisinha;
- Transo quando tenho alguém, mas nunca uso camisinha;

2. Recentemente conheci alguém interessante, rolou um clima...e deu uma vontade enorme de transar. Aí...

- Transamos usando uma camisinha que a gente tinha á mão;
- Quase transamos sem camisinha, mas desistimos;
- Acabamos transando sem camisinha;
- Não passei por essa situação;

3. Já tive relações que podem ter aumentado minha exposição ao HIV?

- Tive relações sexuais com pessoas diferentes. Com algumas eu usei camisinha, com outras não usei;
- Só transei com namorada-com namorado- e não usamos camisinha porque fizemos um pcto de fidelidade;
- Nunca vivi nenhuma dessas situações;

4. A gravidez só acontece quando a gente não faz sexo seguro... Já passei por isso?

- Nunca fiquei grávida-ou engravidei alguém- porque usei camisinha em todas as relações sexuais que já tive;
- Nunca fiquei grávida-ou engravidei alguém- porque uso (ou minha parceira usa) pílula anticoncepcional;
- Já fiquei grávida -ou engravidei alguém – mas nunca fizemos o teste de HIV;

Estragou tudo e a gente embora sem lavar nem fazer mais nada;

Essa situação nunca aconteceu comigo;

8. Acho que a camisinha...

É fácil de colocar;

[illegible]

transando sem camisinha;

Já usei algum (s) dessas drogas, mas nunca cheguei a perder o controle por causa disso;

Já usei droga injetável, utilizando a mesma seringa que as outras pessoas;

Já usei droga injetável com seringa e agulha descartável e ninguém utilizou a mesma seringa;

Nunca vivi nenhuma dessas situações;

6. Uso os serviços de saúde para prevenir (ou tratar) as doenças sexualmente transmissíveis, o HIV e a Aids?

Não. Nunca fui ao médico por causa de questões relacionadas á saúde sexual;

Não. Já tive doença com corrimento, ferida ou verrugas nos órgãos sexuais, mas não procurei o médico e tratei na farmácia;

Sim. Já tive corrimentos e verruga nos órgãos sexuais e tratei depois de fazer consulta médica;

Sim. Faço acompanhamento em um serviço de saúde – de tempos em tempos ou quando tenho algum problema de saúde;

Já tentei, mas não consegui atendimento quando precisei;

7. Durante a relação sexual, a camisinha rompeu-se e...

Não deu para parar e continuamos a transa mesmo assim;

Substituímos a camisinha rompida por uma nova e continuamos;

Nós lavamos os órgãos genitais, pegamos uma camisinha nova e continuamos;

Estragou tudo e a gente embora sem lavar nem fazer mais nada;

- Pode ser usado com prazer;
- Não precisa ser usada com a pessoa que a gente ama e em quem a gente confia;
- Não dá pra usar pois diminui o prazer;
- É bom para evitar filhos, HIV e doenças sexualmente transmissíveis;

9. Eu faria o teste de HIV...

- Só pra saber, por curiosidade;
- Por preocupação com uma situação vivida;
- Não faria o teste porque não quero saber o resultado;
- Eu faria para descobrir, prevenir e tratar logo;

10. Posso contar com outras pessoas pra conversar sobre sexualidade e prevenção?

- Posso. Converso sobre sexualidade e prevenção com pessoas da minha idade;
- Poso contar com um medico ou um profissional da saúde para conversar abertamente sobre assuntos relacionados a sexualidade e prevenção;
- Não tem nenhum adulto com quem eu me sinta á vontade para conversar sobre esse tema;
- Posso contar com algum adulto com quem eu convivo porque sei que ele/ela me dá apoio , mesmo quando a gente tem ideias diferentes;

11. Tenho facilidades ou dificuldades para conseguir camisinha?

- Nunca tenho onde conseguir na hora que eu preciso mas não perco a transa por isso;
- Não tenho onde conseguir;
- Sempre tenho porque compro na farmácia ou no supermercado;
- Ás vezes tenho, as vezes não
- Consigo nos serviços de saúde, escola ou em casa;
- Só meu parceiro-parceira- cuida disso;
- Não procuro porque tenho vergonha ou acho que vão pensar mal de mim

ANEXO H – COMITÊ DE ÉTICA EM PESQUISA

CENTRO UNIVERSITÁRIO DE JOÃO PESSOA - UNIPÊ/PB

Plataforma Brasil

PARECER CONSUBSTANCIADO DO CEP

DADOS DO PROJETO DE PESQUISA

Título da Pesquisa: PROMOVENDO SAÚDE E PREVENINDO DOENÇAS EM CASAS DE APOIO PARA PESSOAS EM SITUAÇÃO DE RUA

Pesquisador: ANNA CLAUDIA FREIRE DE ARAUJO PATRICIO

Área Temática:

Versão: 2

CAAE: 79486517.0.0000.5176

Instituição Proponente: Centro Universitário de João Pessoa - UNIPÊ/PB

Patrocinador Principal: Financiamento Próprio

DADOS DO PARECER

Número do Parecer: 2.456.847

Apresentação do Projeto:

O estudo tem como objetivo analisar aspectos relacionados a saúde e prevenir doenças de pessoas em situações de rua. Trata-se de um estudo transversal, quanti-qualitativo em que participarão 341 pessoas em situação de rua que recebem auxilio da Casa da Acolhida para pessoas em situação de rua, localizado na Rua Capitão José Pessoa, número 65, no bairro de Jaguaribe e no Centro de Referência Especializado para População em Situação de Rua (Creas Pop), localizado na Rua Treze de Maio, 508 – Centro, João Pessoa,Paraíba, Brasil. Serão utilizados instrumentos referentes a dados sociodemográficos, manifestações clínicas, aspectos nutricionais, avaliação odontológica, testes rápidos, comportamento sexual, higiene pessoal e exame físico. Os dados serão coletados em forma de entrevista e exame clínicos, sendo o tempo previsto para cada sujeito do estudo de 40 minutos. Os dados serão processados pelo programa Statistical Package for the Social Sciences versão 19.0. Os dados serão submetidos à estatística descritiva com frequência absoluta e relativa, média, desvio padrão da média, máximo e mínimo. Para testar a associação entre as variáveis serão utilizados testes estatísticos considerando significativo quando o valor p0,05, como o Test t, Qui Quadrado, ANOVA. Os dados qualitativos serão analisados pela Análise de Conteúdo de Bardin.

Endereço: Campus Universitário BR 230 Km 22, Reitoria, sala 401.
Bairro: Água Fria CEP: 58.053-000
UF: PB Município: JOAO PESSOA
Telefone: (83)2106-9266 E-mail: cep@unipe.br

Objetivo da Pesquisa:

Objetivo Primário:

Analisar aspectos relacionados a saúde e prevenir doenças de pessoas em situações de rua.

Objetivo Secundário:

• Identificar parâmetros de saúde bucal, cardiovasculares, comportamentos sexuais, uso de drogas e hábitos de higiene de pessoas em situações de
rua;

• Realizar teste rápido para HIV, sífilis e hepatites de pessoas em situações de rua;

• Orientar pessoas em situações de rua quanto a comportamentos sexuais, higiene pessoal, doenças parasitárias e infectocontagiosas;

• Auxiliar pessoas em situações de rua nos aspectos alimentares, doação de preservativo e materiais de higiene pessoal;

• Intervir nas condições de saúde investigadas neste estudo de pessoas em situações de rua;

• Contribuir para a formação de estudantes de enfermagem e odontologia proporcionando uma visão integral do indivíduo, o respeito às diferenças e
a leitura do contexto sociocultural para uma melhor e eficaz assistência.

Avaliação dos Riscos e Benefícios:

Os riscos e benefícios estão expostos de forma satisfatória, e atendem ao exigido pela Resolução nº 466/12 do Conselho Nacional de Saúde do Ministério da Saúde.

Comentários e Considerações sobre a Pesquisa:

O projeto se apresenta bem estruturado e coerente em relação à ética na pesquisa envolvendo seres humanos.

Considerações sobre os Termos de apresentação obrigatória:

O projeto de pesquisa apresenta Folha de Rosto, Termo de Compromisso do Pesquisador Responsável, Termo de Anuência, instrumento de coleta de dados e Justificativa de não utilização do TCLE adequados ao estudo proposto e de acordo com a Resolução 466/12 do CNS/MS.

Recomendações:

Recomendamos que toda e qualquer alteração seja informada ao devido CEP, sob pena de não aprovação final, bem como a assinatura de todos as vias do TCLE nos termos da Resolução 466/12 item IV.5, alínea d. Ressaltamos que o item V.3 expressa que o pesquisador responsável, ao perceber qualquer risco ou dano significativo ao participante da pesquisa, previstos ou não no

Endereço: Campus Universitário BR 230 Km 22, Reitoria, sala 401.
Bairro: Água Fria **CEP:** 58.053-000
UF: PB **Município:** JOAO PESSOA
Telefone: (83)2106-9286 **E-mail:** cep@unipe.br

Termo de Consentimento Livre e Esclarecido, deve comunicar o fato, imediatamente, ao sistema CEP/CONEP, e avaliar em caráter emergencial a necessidade de adequar ou suspender o estudo.

Conclusões ou Pendências e Lista de Inadequações:

O presente projeto encontra-se devidamente instruído conforme o que preconiza a Resolução nº 466/12, do Conselho Nacional de Saúde - Ministério da Saúde, não apresentando quaisquer pendências ou inadequações. Desse modo somos favoráveis à APROVAÇÃO do mesmo, salvo melhor juízo.

Considerações Finais a critério do CEP:

Este parecer é para fins de execução da referida pesquisa, ficando o pesquisador responsável obrigado a enviar ao Comitê de Ética em Pesquisa (CEP) o RELATÓRIO FINAL da mesma, nos termos das atribuições conferidas ao CEP pela Resolução Nº 466/12 do CNS/MS.

Este parecer foi elaborado baseado nos documentos abaixo relacionados:

Tipo Documento	Arquivo	Postagem	Autor	Situação
Informações Básicas do Projeto	PB_INFORMAÇÕES_BÁSICAS_DO_PROJETO_1006222.pdf	11/12/2017 17:10:37		Aceito
Projeto Detalhado / Brochura Investigador	PROJETOCOMPLETO.doc	11/12/2017 17:01:46	ANNA CLAUDIA FREIRE DE ARAUJO PATRICIO	Aceito
TCLE / Termos de Assentimento / Justificativa de Ausência	APENDICEAtcle.doc	11/12/2017 17:01:08	ANNA CLAUDIA FREIRE DE ARAUJO PATRICIO	Aceito
Outros	ANUENCIA.pdf	24/10/2017 11:30:32	ANNA CLAUDIA FREIRE DE ARAUJO PATRICIO	Aceito
Folha de Rosto	folharostoassinada.pdf	24/10/2017 11:29:14	ANNA CLAUDIA FREIRE DE ARAUJO PATRICIO	Aceito
Outros	APENDICEBinstrumentos.doc	23/10/2017 13:19:52	ANNA CLAUDIA FREIRE DE ARAUJO PATRICIO	Aceito
Outros	TERMOCOMRPOMISSO.pdf	23/10/2017 13:19:29	ANNA CLAUDIA FREIRE DE ARAUJO PATRICIO	Aceito

Situação do Parecer:

Aprovado

CENTRO UNIVERSITÁRIO DE JOÃO PESSOA - UNIPÊ/PB

Plataforma Brasil

Necessita Apreciação da CONEP:
Não

JOAO PESSOA, 26 de Dezembro de 2017

Assinado por:
Dostoievsky Ernesto de Melo Andrade
(Coordenador)

APÊNDICE D

TERMO DE COMPROMISSO DE UTILIZAÇÃO E DIVULGAÇÃO DE DADOS

Eu, Anna Cláudia Freire de Araújo Patrício, doutoranda em Enfermagem pela Universidade Federal do Rio Grande do Norte, pesquisadora responsável do projeto intitulado **PROMOVENDO SAÚDE E PREVENINDO DOENÇAS EM CASAS DE APOIO PARA PESSOAS EM SITUAÇÃO DE RUA** , declaro que conheço e cumprirei as normas vigentes expressas na **Resolução Nº466/12 do Conselho Nacional de Saúde/Ministério da Saúde,** e em suas complementares**,** e assumo, neste Termo, o compromisso de, ao utilizar dados e/ou informações coletados assegurar a confidencialidade e a privacidade dos mesmos. Assumimos ainda neste Termo o compromisso de destinar os dados coletados somente para o projeto ao qual se vinculam.

João Pessoa, ______ de ___________________ de 20__.

____
Pesquisador Responsável

____
Pesquisador Participante

____
Pesquisador Participante

____
Pesquisador Participante

____
Pesquisador Participante

____
Pesquisador Participante

ANEXO I – ANUÊNCIA

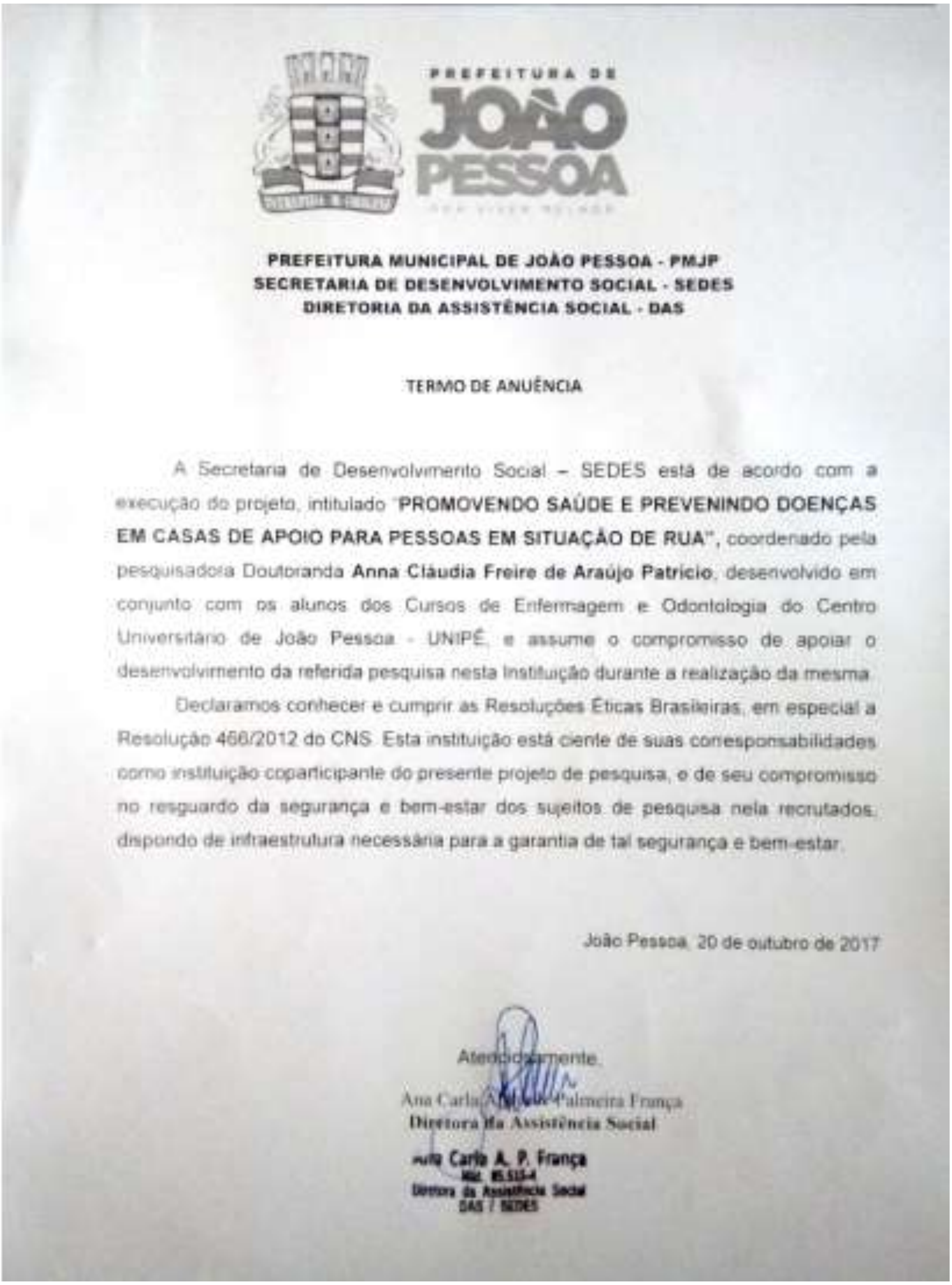

PREFEITURA DE JOÃO PESSOA

PREFEITURA MUNICIPAL DE JOÃO PESSOA - PMJP
SECRETARIA DE DESENVOLVIMENTO SOCIAL - SEDES
DIRETORIA DA ASSISTÊNCIA SOCIAL - DAS

TERMO DE ANUÊNCIA

A Secretaria de Desenvolvimento Social – SEDES está de acordo com a execução do projeto, intitulado "**PROMOVENDO SAÚDE E PREVENINDO DOENÇAS EM CASAS DE APOIO PARA PESSOAS EM SITUAÇÃO DE RUA**", coordenado pela pesquisadora Doutoranda **Anna Cláudia Freire de Araújo Patrício**, desenvolvido em conjunto com os alunos dos Cursos de Enfermagem e Odontologia do Centro Universitário de João Pessoa - UNIPÊ, e assume o compromisso de apoiar o desenvolvimento da referida pesquisa nesta Instituição durante a realização da mesma.

Declaramos conhecer e cumprir as Resoluções Éticas Brasileiras, em especial a Resolução 466/2012 do CNS. Esta instituição está ciente de suas corresponsabilidades como instituição coparticipante do presente projeto de pesquisa, e de seu compromisso no resguardo da segurança e bem-estar dos sujeitos de pesquisa nela recrutados, dispondo de infraestrutura necessária para a garantia de tal segurança e bem-estar.

João Pessoa, 20 de outubro de 2017

Atenciosamente,

Ana Carla [illegible] Palmeira França
Diretora da Assistência Social

Ana Carla A. P. França
Mat. 95.515-4
Diretora da Assistência Social
DAS / SEDES

FUNÇÕES E TESTES NO SOFTWARE R

```
library(poLCA)
B<-read.csv2("H:/SOCIODM.csv", header=T)
f <- cbind(IDADE, GENERO,COMPANHEIRO, RESILIENCIA, DEPRESSAO,
TRANST.MENTAIS, TEMPOMORADIA, ORAL, ANAL, VAGINAL,ANSIEDADE,
DESESPERANÇA)~1
```

```
lca2 <- poLCA(f,B,nclass=2)
lca3 <- poLCA(f,B,nclass=3)
lca4 <- poLCA(f,B,nclass=4,nrep=10,maxiter=5000)
```

ENTROPIA RELATIVA

```
#DUAS CLASSES
k=2
p.hat2<-lca2$predcell$observed/lca2$N
H.hat2<-   -sum(p.hat2*log(p.hat2))
H.hat2
EntropiaRelativa2 = (1 - H.hat2/(nrow(B)*log(k)))*100
EntropiaRelativa2

#TRES CLASSES
k=3
p.hat3<-lca3$predcell$observed/lca3$N
H.hat3<-   -sum(p.hat3*log(p.hat3))
H.hat3
EntropiaRelativa3 = (1 - H.hat3/(nrow(B)*log(k)))*100
EntropiaRelativa3

#QUATRO CLASSES
k=4
p.hat4<-lca4$predcell$observed/lca4$N
H.hat4<-   -sum(p.hat4*log(p.hat4))
H.hat4
EntropiaRelativa4 = (1 - H.hat4/(nrow(B)*log(k)))*100
EntropiaRelativa4
```

Modelo de classe latente (Latent Class model = LCA)

```
R version 3.4.1 (2017-06-30) -- "Single Candle"
Copyright (C) 2017 The R Foundation for Statistical Computing
Platform: x86_64-w64-mingw32/x64 (64-bit)

R é um software livre e vem sem GARANTIA ALGUMA.
Você pode redistribuí-lo sob certas circunstâncias.
Digite 'license()' ou 'licence()' para detalhes de distribuição.

R é um projeto colaborativo com muitos contribuidores.
Digite 'contributors()' para obter mais informações e
'citation()' para saber como citar o R ou pacotes do R em
publicações.

Digite 'demo()' para demonstrações, 'help()' para o sistema on-line
de ajuda,
ou 'help.start()' para abrir o sistema de ajuda em HTML no seu
navegador.
Digite 'q()' para sair do R.

[Área de trabalho anterior carregada]

> library(poLCA)
Carregando pacotes exigidos: scatterplot3d
Carregando pacotes exigidos: MASS
Warning messages:
1: package ‘poLCA’ was built under R version 3.4.4
2: package ‘scatterplot3d’ was built under R version 3.4.4
> B<-read.csv2("H:/SOCIODM.csv", header=T)
> f <- cbind(IDADE, GENERO,COMPANHEIRO, RESILIENCIA, DEPRESSAO,
TRANST.MENTAIS, TEMPOMORADIA, ORAL, ANAL, VAGINAL,ANSIEDADE,
DESESPERANÇA)~1
>
>
>
>
> lca2 <- poLCA(f,B,nclass=2)
Conditional item response (column) probabilities,
 by outcome variable, for each class (row)

$IDADE
           Pr(1)  Pr(2)  Pr(3)
class 1:  0.4333 0.2647 0.3020
class 2:  0.2875 0.4612 0.2513

$GENERO
           Pr(1)  Pr(2)
class 1:  0.1271 0.8729
class 2:  0.2029 0.7971

$COMPANHEIRO
           Pr(1)  Pr(2)  Pr(3)
```

```
class 1:  0.7752 0.1285 0.0964
class 2:  0.5330 0.3392 0.1278

$RESILIENCIA
           Pr(1)  Pr(2)  Pr(3)
class 1:  0.4091 0.3110 0.2799
class 2:  0.5724 0.3317 0.0959

$DEPRESSAO
           Pr(1)  Pr(2)
class 1:  0.5399 0.4601
class 2:  0.8856 0.1144

$TRANST.MENTAIS
           Pr(1)  Pr(2)
class 1:  0.6529 0.3471
class 2:  0.9687 0.0313

$TEMPOMORADIA
           Pr(1)  Pr(2)  Pr(3)
class 1:  0.2754 0.3740 0.3507
class 2:  0.3321 0.0424 0.6255

$ORAL
           Pr(1)  Pr(2)
class 1:  0.7702 0.2298
class 2:  1.0000 0.0000

$ANAL
           Pr(1)  Pr(2)
class 1:  0.6642 0.3358
class 2:  1.0000 0.0000

$VAGINAL
           Pr(1)  Pr(2)
class 1:  0.9823 0.0177
class 2:  1.0000 0.0000

$ANSIEDADE
           Pr(1)  Pr(2)  Pr(3)  Pr(4)
class 1:  0.4951 0.3759 0.0582 0.0707
class 2:  0.2070 0.3623 0.4307 0.0000

$DESESPERANÇA
           Pr(1)  Pr(2)  Pr(3)  Pr(4)
class 1:  0.4755 0.2125 0.2336 0.0784
class 2:  0.0483 0.3910 0.2944 0.2663

Estimated class population shares
 0.5658 0.4342

Predicted class memberships (by modal posterior prob.)
 0.57 0.43

=========================================================
Fit for 2 latent classes:
```

```
========================================================
number of observations: 100
number of estimated parameters: 41
residual degrees of freedom: 59
maximum log-likelihood: -874.5801

AIC(2): 1831.16
BIC(2): 1937.972
G^2(2): 847.5343 (Likelihood ratio/deviance statistic)
X^2(2): 54949.66 (Chi-square goodness of fit)

> #DUAS CLASSES
> k=2
> p.hat2<-lca2$predcell$observed/lca2$N
> H.hat2<-   -sum(p.hat2*log(p.hat2))
> H.hat2
[1] 4.50813
> EntropiaRelativa2 = (1 - H.hat2/(nrow(B)*log(k)))*100
> EntropiaRelativa2
[1] 93.49614
>
>
>
>
>
> lca3 <- poLCA(f,B,nclass=3)
Conditional item response (column) probabilities,
 by outcome variable, for each class (row)

$IDADE
           Pr(1)  Pr(2)  Pr(3)
class 1:  0.1544 0.5053 0.3403
class 2:  0.3889 0.2383 0.3728
class 3:  0.6227 0.2095 0.1678

$GENERO
           Pr(1)  Pr(2)
class 1:  0.1109 0.8891
class 2:  0.1909 0.8091
class 3:  0.2062 0.7938

$COMPANHEIRO
           Pr(1)  Pr(2)  Pr(3)
class 1:  0.6330 0.2168 0.1501
class 2:  0.6272 0.2486 0.1243
class 3:  0.7328 0.2118 0.0554

$RESILIENCIA
           Pr(1)  Pr(2)  Pr(3)
class 1:  0.4398 0.4519 0.1083
class 2:  0.3889 0.2486 0.3625
class 3:  0.5672 0.1908 0.2420

$DEPRESSAO
           Pr(1)  Pr(2)
class 1:  0.7696 0.2304
```

```
class 2:  0.6272 0.3728
class 3:  0.6204 0.3796

$TRANST.MENTAIS
           Pr(1)  Pr(2)
class 1:  0.8156 0.1844
class 2:  0.5753 0.4247
class 3:  0.8501 0.1499

$TEMPOMORADIA
           Pr(1)  Pr(2)  Pr(3)
class 1:  0.0000 0.1725 0.8275
class 2:  0.3095 0.1313 0.5593
class 3:  0.6587 0.3413 0.0000

$ORAL
           Pr(1)  Pr(2)
class 1:  1.0000 0.0000
class 2:  0.1922 0.8078
class 3:  1.0000 0.0000

$ANAL
           Pr(1)  Pr(2)
class 1:  1.0000 0.0000
class 2:  0.0000 1.0000
class 3:  0.9235 0.0765

$VAGINAL
           Pr(1)  Pr(2)
class 1:  1.0000 0.0000
class 2:  1.0000 0.0000
class 3:  0.9737 0.0263

$ANSIEDADE
           Pr(1)  Pr(2)  Pr(3)  Pr(4)
class 1:  0.3058 0.3971 0.2776 0.0195
class 2:  0.6111 0.2621 0.0646 0.0621
class 3:  0.3455 0.3829 0.2161 0.0554

$DESESPERANÇA
           Pr(1)  Pr(2)  Pr(3)  Pr(4)
class 1:  0.1853 0.2123 0.3902 0.2122
class 2:  0.5683 0.1243 0.1786 0.1288
class 3:  0.2987 0.4542 0.1370 0.1101

Estimated class population shares
 0.4592 0.1609 0.3798

Predicted class memberships (by modal posterior prob.)
 0.47 0.16 0.37

=========================================================
Fit for 3 latent classes:
=========================================================
number of observations: 100
number of estimated parameters: 62
```

```
residual degrees of freedom: 38
maximum log-likelihood: -852.1212

AIC(3): 1828.242
BIC(3): 1989.763
G^2(3): 802.6166 (Likelihood ratio/deviance statistic)
X^2(3): 20878.59 (Chi-square goodness of fit)

>
>
>
> k=3
> p.hat3<-lca3$predcell$observed/lca3$N
> H.hat3<-   -sum(p.hat3*log(p.hat3))
> H.hat3
[1] 4.50813
> EntropiaRelativa3 = (1 - H.hat3/(nrow(B)*log(k)))*100
> EntropiaRelativa3
[1] 95.89652
>
>
>
> lca4 <- poLCA(f,B,nclass=4,nrep=10,maxiter=5000)
Model 1: llik = -823.2118 ... best llik = -823.2118
Model 2: llik = -828.2731 ... best llik = -823.2118
Model 3: llik = -816.6712 ... best llik = -816.6712
Model 4: llik = -822.536 ... best llik = -816.6712
Model 5: llik = -827.6522 ... best llik = -816.6712
Model 6: llik = -823.856 ... best llik = -816.6712
Model 7: llik = -833.8786 ... best llik = -816.6712
Model 8: llik = -822.6145 ... best llik = -816.6712
Model 9: llik = -823.2693 ... best llik = -816.6712
Model 10: llik = -832.3235 ... best llik = -816.6712
Conditional item response (column) probabilities,
 by outcome variable, for each class (row)

$IDADE
           Pr(1)  Pr(2)  Pr(3)
class 1:  0.4357 0.2930 0.2712
class 2:  0.2739 0.3239 0.4021
class 3:  0.0000 0.8801 0.1199
class 4:  0.5589 0.1014 0.3397

$GENERO
           Pr(1)  Pr(2)
class 1:  0.0909 0.9091
class 2:  0.1620 0.8380
class 3:  0.1435 0.8565
class 4:  0.2420 0.7580

$COMPANHEIRO
           Pr(1)  Pr(2)  Pr(3)
class 1:  0.8045 0.0726 0.1230
class 2:  0.5979 0.3212 0.0810
class 3:  0.6146 0.3854 0.0000
class 4:  0.5885 0.2383 0.1733
```

```
$RESILIENCIA
           Pr(1)  Pr(2)  Pr(3)
class 1:  0.4029 0.3158 0.2813
class 2:  0.2480 0.3212 0.4309
class 3:  0.4068 0.5376 0.0555
class 4:  0.6921 0.1932 0.1147

$DEPRESSAO
           Pr(1)  Pr(2)
class 1:  0.3714 0.6286
class 2:  0.5141 0.4859
class 3:  0.8428 0.1572
class 4:  1.0000 0.0000

$TRANST.MENTAIS
           Pr(1)  Pr(2)
class 1:  0.6572 0.3428
class 2:  0.4332 0.5668
class 3:  0.8957 0.1043
class 4:  1.0000 0.0000

$TEMPOMORADIA
           Pr(1)  Pr(2)  Pr(3)
class 1:  0.2814 0.4504 0.2682
class 2:  0.4070 0.0839 0.5091
class 3:  0.0000 0.0000 1.0000
class 4:  0.4598 0.1904 0.3498

$ORAL
           Pr(1)  Pr(2)
class 1:  1.0000 0.0000
class 2:  0.1901 0.8099
class 3:  1.0000 0.0000
class 4:  0.9092 0.0908

$ANAL
           Pr(1)  Pr(2)
class 1:  1.0000 0.0000
class 2:  0.0000 1.0000
class 3:  1.0000 0.0000
class 4:  0.7985 0.2015

$VAGINAL
           Pr(1)  Pr(2)
class 1:  0.9713 0.0287
class 2:  1.0000 0.0000
class 3:  1.0000 0.0000
class 4:  1.0000 0.0000

$ANSIEDADE
           Pr(1)  Pr(2)  Pr(3)  Pr(4)
class 1:  0.4884 0.4322 0.0000 0.0794
class 2:  0.8098 0.1902 0.0000 0.0000
class 3:  0.5042 0.1218 0.3740 0.0000
class 4:  0.0000 0.5207 0.4418 0.0375
```

```
$DESESPERANÇA
           Pr(1)  Pr(2)  Pr(3)  Pr(4)
class 1:  0.4130 0.3469 0.2402 0.0000
class 2:  0.6732 0.0810 0.1620 0.0839
class 3:  0.0925 0.0556 0.5462 0.3057
class 4:  0.1356 0.4490 0.1457 0.2697

Estimated class population shares
 0.3481 0.1235 0.1983 0.3301

Predicted class memberships (by modal posterior prob.)
 0.34 0.12 0.2 0.34

=========================================================
Fit for 4 latent classes:
=========================================================
number of observations: 100
number of estimated parameters: 83
residual degrees of freedom: 17
maximum log-likelihood: -816.6712

AIC(4): 1799.342
BIC(4): 2015.571
G^2(4): 731.7164 (Likelihood ratio/deviance statistic)
X^2(4): 17404.92 (Chi-square goodness of fit)

> k=4
> p.hat4<-lca4$predcell$observed/lca4$N
> H.hat4<-   -sum(p.hat4*log(p.hat4))
> H.hat4
[1] 4.50813
> EntropiaRelativa4 = (1 - H.hat4/(nrow(B)*log(k)))*100
> EntropiaRelativa4
[1] 96.74807
>
>
```

CONFIRMAÇÃO MODELO CLASSE LATENTE: 3 CLASSES

```
library(lavaan)
FREIRE<-read.csv2("F:/SEMFREIRE.csv", header= T)
HS.model<-  'Comportamental=~ORAL+ANAL+VAGINAL
            Psiquica =~ ANSIEDADE+DESESPERANÇA+DEPRESSAO+
RESILIENCIA+ TRANSMENTAL
            Sociodemografica =~IDADE+GENERO + GENERO+ COMPANHEIRO +
TMORADIA'
fit<- cfa(HS.model,data=FREIRE)
summary(fit, fit.measures=TRUE)
```

```
lavaan (0.5-20) converged normally after 125 iterations

  Number of observations                           100

  Estimator                                         ML
  Minimum Function Test Statistic               53.373
  Degrees of freedom                                51
  P-value (Chi-square)                           0.383

Model test baseline model:

  Minimum Function Test Statistic              235.701
  Degrees of freedom                                66
  P-value                                        0.000

User model versus baseline model:

  Comparative Fit Index (CFI)                    0.986
  Tucker-Lewis Index (TLI)                       0.982

Loglikelihood and Information Criteria:

  Loglikelihood user model (H0)              -1286.480
  Loglikelihood unrestricted model (H1)      -1259.793

  Number of free parameters                         27
  Akaike (AIC)                                2626.959
  Bayesian (BIC)                              2697.299
  Sample-size adjusted Bayesian (BIC)         2612.026

Root Mean Square Error of Approximation:

  RMSEA                                          0.022
  90 Percent Confidence Interval          0.000  0.069
  P-value RMSEA <= 0.05                          0.795

Standardized Root Mean Square Residual:

  SRMR                                           0.075

Parameter Estimates:
```

```
  Information                                   Expected
  Standard Errors                               Standard

Latent Variables:
                      Estimate  Std.Err  Z-value  P(>|z|)
  Comportamental =~
    ORAL                 1.000
    ANAL                 0.943    0.281    3.357    0.001
    VAGINAL             -0.012    0.030   -0.388    0.698
  Psiquica =~
    ANSIEDADE            1.000
    DESESPERANÇA         1.101    0.345    3.193    0.001
    DEPRESSAO           -0.566    0.165   -3.421    0.001
    RESILIENCIA         -0.251    0.205   -1.222    0.222
    TRANSMENTAL         -0.500    0.146   -3.424    0.001
  Sociodemografica =~
    IDADE                1.000
    GENERO              -0.003    0.007   -0.382    0.702
    COMPANHEIRO          0.022    0.015    1.490    0.136
    TMORADIA             1.095    0.576    1.899    0.058

Covariances:
                    Estimate  Std.Err  Z-value  P(>|z|)
  Comportamental ~~
    Psiquica          -0.037    0.021   -1.722    0.085
    Sociodemografc     0.392    0.289    1.357    0.175
  Psiquica ~~
    Sociodemografc     0.267    0.464    0.577    0.564

Variances:
                     Estimate  Std.Err  Z-value  P(>|z|)
    ORAL                0.001    0.032    0.045    0.964
    ANAL                0.055    0.030    1.840    0.066
    VAGINAL             0.010    0.001    7.071    0.000
    ANSIEDADE           0.516    0.096    5.369    0.000
    DESESPERANÇA        0.828    0.143    5.796    0.000
    DEPRESSAO           0.141    0.028    5.066    0.000
    RESILIENCIA         0.587    0.084    6.981    0.000
    TRANSMENTAL         0.109    0.022    5.048    0.000
    IDADE              55.874   20.956    2.666    0.008
    GENERO              0.134    0.019    7.063    0.000
    COMPANHEIRO         0.449    0.065    6.918    0.000
    TMORADIA           39.546   23.885    1.656    0.098
    Comportamental      0.112    0.036    3.106    0.002
    Psiquica            0.229    0.098    2.325    0.020
    Sociodemografica   37.559   22.110    1.699    0.089
```
